# Comparative effectiveness of non-pharmacological interventions for anxiety, depression, and quality of life in individuals with autism spectrum disorder: A systematic review and network meta-analysis

**DOI:** 10.3389/fpsyt.2025.1660412

**Published:** 2025-12-02

**Authors:** Xianming Ding, Haoran Luo, Junyu Zhang, Hongyu Yang, Yue Fan, Jinyan Wu, Sai Wu

**Affiliations:** 1 School of Sports Science, Jishou University, Jishou, China; 2 Institute of Physical Culture, Sports and Tourism, Peter the Great St. Petersburg Polytechnic University, Saint Petersburg, Russia; 3 School of Competitive Sports, Beijing Sport University, Beijing, China; 4 Football Sports College, Chengdu Sport University, Chengdu, China; 5 College of Physical Education, Southwest University, Chongqing, China

**Keywords:** non-pharmacological intervention, autism spectrum disorder, depressive symptoms, anxiety symptoms, quality of life, network meta-analysis

## Abstract

**Background:**

Individuals with autism spectrum disorder (ASD) commonly experience comorbid depression, anxiety, and impaired quality of life (QoL), significantly affecting daily functioning and social adaptation. Non-pharmacological interventions (NPIs), offering an alternative without drug-related side effects, have gained increasing attention for emotional improvement and health promotion. However, the comparative effectiveness of different NPIs remains unclear, and clinical decisions lack robust evidence.

**Methods:**

This study adhered to the PRISMA-NMA guidelines. Five databases (PubMed, Embase, Cochrane Library, Web of Science, and EBSCOhost) were systematically searched to identify randomized controlled trials (RCTs) published before March 2025. A total of 67 RCTs involving 3,604 ASD participants were included. A frequentist network meta-analysis using a multivariate random-effects model was conducted in Stata, along with pairwise meta-analyses, to compare the relative effects of mindfulness-based interventions (MBI), cognitive behavioral therapy (CBT), behavioral and functional training (BEHAVE), physical activity (PHYS), sensory therapies (SENS), technology- and family-based interventions (TAFI), and other interventions (OTH) on anxiety, depression, and QoL. Standardized mean differences (SMDs) with 95% credible intervals (CIs) were used to estimate effects, and SUCRA rankings were calculated to assess comparative efficacy.

**Results:**

MBI showed the greatest improvement in anxiety symptoms (SMD = –0.84, 95% CI: –1.32 to –0.36; SUCRA = 91.4%), CBT ranked highest for depression reduction (SMD = –0.77, 95% CI: –1.25 to –0.28; SUCRA = 90.1%), and PHYS performed best for enhancing QoL (SMD = 0.59, 95% CI: 0.20 to 0.98; SUCRA = 87.5%). The analyzed population primarily consisted of high-functioning male individuals. Subgroup analyses showed stronger effects in adults and with moderate-duration interventions (9-16weeks). No significant inconsistency or publication bias was detected.

**Limitations:**

Findings mainly apply to high-functioning ASD populations without intellectual disability. Heterogeneity in interventions and assessments should be considered.

**Conclusions:**

Different NPIs exhibit distinct advantages in improving emotional symptoms and QoL among individuals with ASD. MBI, CBT, and PHYS demonstrate relative superiority for anxiety, depression, and QoL respectively, supporting their targeted application in clinical and rehabilitative settings. Future studies should prioritize long-term follow-up, refined intervention designs, and personalized strategies tailored to ASD subgroups to enhance clinical utility and scalability.

**Systematic review registration:**

https://www.crd.york.ac.uk/prospero/, identifier CRD420251021423.

## 1 Introduction

Autism Spectrum Disorder (ASD) is a heterogeneous neurodevelopmental condition characterized by core social communication deficits and restricted, repetitive behaviors, which pose lifelong challenges to individual development (1). Globally, the prevalence of ASD has risen significantly, making it a major public health concern. According to the U.S. Centers for Disease Control and Prevention (2), the estimated prevalence among 8-year-old children in the United States reached 2.8% (approximately 1 in 36) in 2020. Similarly, epidemiological surveys in China indicate a childhood prevalence exceeding 1% (3). Of particular concern is the high co-occurrence of significant anxiety and depressive symptoms in individuals with ASD. A systematic review encompassing 32 countries reported pooled prevalence rates of 39.6% for anxiety and 23% for depression, with rates exceeding 50% in subgroups such as adolescents and adults (4, 5). These emotional comorbidities not only exacerbate social functional impairments but also significantly increase the risks of self-injury and suicide (6), severely compromising patients’ quality of life (QoL) and family well-being. Consequently, identifying safe and effective intervention strategies to improve emotional symptoms and QoL represents a core priority in contemporary ASD clinical practice.

Current clinical management of emotional problems in ASD primarily relies on pharmacological interventions, such as selective serotonin reuptake inhibitors (SSRIs) and second-generation antipsychotics. However, the application of these medications in the ASD population faces notable limitations, including low response rates (approximately 30-40%), frequent metabolic side effects (e.g., weight gain), and suboptimal long-term treatment adherence (7). In contrast, non-pharmacological interventions (NPIs), owing to their superior safety profile and acceptability, are increasingly becoming vital options in clinical practice. For this study, NPIs are defined as structured, evidence-based therapeutic approaches that explicitly exclude the use of pharmaceutical agents and neurostimulation techniques. These interventions primarily operate through psychological, behavioral, physiological, or social mechanisms and are typically delivered by professionals, caregivers, or via digital platforms in clinical, educational, or community settings (8). To enable systematic comparison of NPI effects, this study adopts the intervention classification framework by Hume et al. (9), categorizing NPIs into seven main types based on their core mechanisms and delivery formats, which are compared against a control group (CTRL, including waitlist or treatment-as-usual): (1) Mindfulness-Based Interventions (MBI), e.g., mindfulness-based stress reduction; (2) Cognitive Behavioral Therapy (CBT), including cognitive restructuring and exposure therapy; (3) Behavioral and Functional Training (BEHAVE), focusing on social and adaptive skills; (4) Physical Activity (PHYS); (5) Sensory Therapy (SENS); (6) Technology-Assisted and Family Intervention (TAFI); and (7) Other Therapies (OTH), such as animal-assisted therapy. Although existing evidence supports the efficacy of CBT for alleviating anxiety in adolescents with ASD (10) and mindfulness training for improving emotion regulation in adults with ASD (11), the current evidence base possesses CItical limitations.

First, while a protocol for a network meta-analysis (NMA) of NPIs for ASD has been published, its final results are not yet available (12). Furthermore, that protocol primarily focuses on core ASD symptoms and does not provide comparative effectiveness evidence for key emotional outcomes such as anxiety, depression, and QoL. Second, most existing systematic reviews concentrate on single intervention types or single outcome measures, lacking a comprehensive head-to-head comparison of different NPIs across multidimensional psychological outcomes. Finally, the influence of key moderating variables, such as participant age, functional level, and intervention duration, has not been systematically examined (5). These limitations hinder clinicians’ ability to select the optimal intervention based on individual characteristics (e.g., age, co-morbidities).

To address these gaps, this study systematically integrates evidence from randomized controlled trials (RCTs) and employs a frequentist framework network meta-analysis to compare the relative effectiveness of the seven major categories of NPIs in improving anxiety, depressive symptoms, and QoL in individuals with ASD. We propose the following hypotheses: (1) Different NPIs exhibit superiority effects for specific outcomes, namely MBI for anxiety, CBT for depression, and PHYS for QoL; (2) Intervention effects are moderated by age and intervention duration, showing greater efficacy in adults, high-functioning individuals, and interventions of medium duration (9–16 weeks). This study will construct a mixed-treatment comparison network, calculate the surface under the cumulative ranking curve (SUCRA), and conduct pre-specified subgroup analyses. The aim is to provide high-level evidence for informing precise clinical intervention pathways for ASD and to guide the optimal allocation of public health and rehabilitation service resources.

## 2 Methods

This systematic review and network meta-analysis was conducted and reported in accordance with the Preferred Reporting Items for Systematic Reviews and Meta-Analyses incorporating Network Meta-Analyses (PRISMA-NMA) guidelines to ensure methodological transparency and standardized reporting of results (13). The study protocol was prospectively registered on the International Prospective Register of Systematic Reviews (PROSPERO; Registration No.: CRD420251021423). PROSPERO is an open-access platform developed by the Centre for Reviews and Dissemination (CRD) in the United Kingdom to enhance the traceability and reproducibility of non-Cochrane systematic reviews and to support evidence-based decision-making.

### 2.1 Data sources and searches

A comprehensive literature search was conducted in five electronic databases: PubMed, Embase, Cochrane Library, Web of Science, and EBSCOhost. The search covered all available records from database inception through March 28, 2025. The initial strategy was developed based on prior systematic reviews, with a core query formulated in PubMed and subsequently adapted to meet the syntax and indexing requirements of the remaining databases (14).

Two reviewers (DXM and LHR) independently conducted the search using the following Boolean string:(“autism spectrum disorder” OR ASD OR “autistic disorder”) AND (“cognitive behavioral therapy” OR CBT OR mindfulness OR “physical activity” OR exercise OR yoga OR music OR “sensory integration” OR hippotherapy OR exergaming OR PEERS OR “social skills training”) AND (anxiety OR depression OR “quality of life” OR “mental health”) AND (“randomized controlled trial” OR RCT OR “controlled clinical trial” OR “random allocation”).The complete PubMed search strategy is presented in **Supplementary Methods 1**, with minor adjustments applied for the other databases. To enhance search sensitivity, wildcard characters (e.g., “*”) were used in free-text terms to capture spelling variants. Eligible studies were restricted to English-language randomized controlled trials (RCTs) involving participants diagnosed with ASD according to standardized operational criteria (e.g., DSM-5 or ICD-10), and reporting at least one outcome related to anxiety, depression, or quality of life. Two reviewers (DXM and LHR) independently performed the literature search and title/abstract screening. Discrepancies were resolved by consensus or adjudication by a third reviewer (ZJY). To minimize the risk of publication bias and ensure timely and comprehensive evidence inclusion, we also manually screened the reference lists of recent high-quality systematic reviews and searched trial registries for unpublished or ongoing studies potentially containing relevant and representative data.

### 2.2 Inclusion and exclusion criteria

Prior to formal screening, we systematically reviewed recently published systematic reviews and network meta-analyses on ASD to delineate the practical boundaries of common intervention types and eligibility criteria, thereby informing subsequent intervention classification and quality control (15). By synthesizing intervention approaches, sample characteristics, and outcome measures from existing literature, we preliminarily established the intervention stratification logic and data extraction structure for this study, ensuring the scientific rigor and systematicity of the screening strategy.

To guarantee the rigor and reproducibility of the screening process, all bibliographic records retrieved from databases were imported into EndNote X9 (Clarivate Analytics, Philadelphia, PA, USA) for management and duplicate removal. Subsequently, two investigators (DXM, LHR) independently screened titles and abstracts. Potentially eligible studies were retrieved for full-text review to determine final inclusion. Any discrepancies during the screening process were resolved through consensus or adjudication by a third investigator (ZJY). The entire screening process strictly adhered to the PICOS (Population, Intervention, Comparison, Outcome, Study design) framework to ensure clinical and methodological homogeneity among the included studies.

The inclusion criteria were as follows:

i) Randomized controlled trial (RCT) design;ii) Study population comprising individuals diagnosed with ASD using standardized criteria such as DSM or ICD, irrespective of age, sex, or ethnicity;iii) Intervention involving any structured non-pharmacological, non-invasive approach (e.g., cognitive behavioral therapy, mindfulness-based intervention, physical activity, social skills training, sensory therapy);iv) Control group receiving no intervention, waitlist, treatment-as-usual, or non-specific psychoeducation;v) Reporting of at least one quantitative outcome measure related to anxiety, depression, or quality of life;vi) Full-text publication in English following peer review.

The exclusion criteria were as follows:

i) Study population comprising non-ASD individuals or those with comorbid severe psychotic disorders (e.g., schizophrenia) or neurological diseases (e.g., epilepsy) that could significantly alter core symptoms or response to intervention compared to individuals with ASD alone;ii) Non-randomized or quasi-randomized trial designs (e.g., cohort studies, case-control studies, single-group pre-post designs);iii) Intervention packages including pharmacological agents or neuroregulatory techniques (e.g., repetitive transcranial magnetic stimulation);iv) Publication types such as systematic reviews, overviews, qualitative studies, study protocols, conference abstracts, dissertations, preprints, and other grey literature;v) Duplicate publications from the same clinical trial, in which case only the version with the most complete data or largest sample size was retained;vi) Missing key data (e.g., means, standard deviations) that could not be obtained by contacting the corresponding author.

Note on the Exclusion of Grey Literature: The exclusion criteria for this study were intentionally strict, explicitly precluding all forms of grey literature. This decision was made primarily to prioritize the methodological quality and reporting standardization of the included evidence, as the peer-review process is a crucial safeguard for data completeness and study rigor. We acknowledge that this approach may have led to the omission of the most recent data on emerging interventions or studies with significant results prior to formal journal publication. However, through tracing references of the included published studies and screening citations of relevant systematic reviews, we did not identify any excluded grey literature whose reported findings substantially contradicted the conclusions derived from the ultimately included peer-reviewed studies. This limitation is further elaborated in the Discussion section.

### 2.3 Data extraction and quality assessment

Data extraction and quality assessment were independently conducted by two reviewers (DXM and LHR). Discrepancies were resolved through discussion or adjudicated by a third reviewer (ZJY). The following data were extracted: i) basic study information, including first author, year of publication, and country; ii) participant characteristics, including sample size, gender distribution, age, and diagnostic CIteria; iii) intervention details, including name, type, frequency, duration per session, and total intervention period; and iv) outcome measures related to anxiety, depression, and quality of life (QoL).When essential statistics (e.g., means and standard deviations) were not directly reported, estimates were calculated from medians, interquartile ranges, or p-values following Cochrane Handbook guidelines (16, 17). Calculation formulas are detailed in **Supplementary Methods 2**. Where necessary, GetData software (version 2.20) was used to extract data from graphs. If CItical data were missing, attempts were made to contact the corresponding authors. Studies without sufficient response were excluded from the analysis.

To allow systematic comparison across intervention types, all interventions were re-coded and categorized based on their underlying mechanism, operational form, and delivery modality. Interventions were ultimately classified into eight categories:

i) CTRL – control groups, including no treatment, usual care, or waitlist;ii) MBI – mindfulness-based interventions (e.g., MBSR);iii) CBT – cognitive behavioral therapy, including dialectical behavior therapy;iv) BEHAVE – behavioral and functional training (e.g., PEERS, STEPS, transitional support, social education programs);v) PHYS – physical activity programs (e.g., AllPlay Dance, Auskick Football, LEGO-based therapy, recreational sports, equine therapy);vi) SENS – sensory-based therapies (e.g., hydrotherapy, aromatherapy, visual supports, sensory integration);vii) TAFI – technology-assisted and family-based interventions (e.g., VR-based games, MindLight, parent training, in-home implementation plans);viii) OTH – other interventions, including animal-assisted therapy, drama therapy, sleep training, and growth mindset programs. This categorization of interventions was informed by prior systematic reviews and intervention classification methodologies (9), aiming to balance clinical utility with analytical feasibility. This framework facilitates the control of heterogeneity in the subsequent network meta-analysis and enhances the comparability of effects across multiple interventions. We acknowledge that some intervention categories (e.g., TAFI, OTH) are inherently multimodal in nature. Future applications of more refined methodologies, such as component network meta-analysis, would be valuable for delineating their active components. Detailed definitions of the interventions and control conditions are provided in **Supplementary Table S1**.

The risk of bias for included studies was assessed using the Cochrane Risk of Bias tool 2.0 (RoB 2.0) as recommended in the Cochrane Handbook (18). Two reviewers (FY and WS) independently completed the assessments, with disagreements resolved by a third reviewer (DXM). The RoB 2.0 tool evaluates the following seven domains: i) random sequence generation; ii) allocation concealment; iii) blinding of participants and personnel; iv) blinding of outcome assessors; v) completeness of outcome data; vi) selective outcome reporting; and vii) other potential sources of bias. Each domain was rated as “low risk,” “high risk,” or “unclear risk.” Risk of bias was assessed separately for each outcome domain (anxiety, depression, and quality of life) to ensure specificity and accuracy in quality appraisal.

### 2.4 Data synthesis and statistical analyses

The data analysis process began by extracting all available pairwise comparisons from eligible studies, followed by both pairwise and network meta-analyses. Statistical analyses were conducted using Review Manager 5.3 and Stata 17.0 (StataCorp LLC, College Station, TX), with the network suite module applied for network estimation. All continuous outcomes were standardized, and either mean difference (MD) or standardized mean difference (SMD) was used as the effect size, accompanied by 95% confidence intervals (CIs) and predictive intervals (PIs). For anxiety, depression, and quality of life (QoL) outcomes, we prioritized the most psychometrically robust scales based on a predefined hierarchy. Score directions were harmonized to ensure that higher scores consistently reflected more severe symptoms or better QoL, depending on the construct. A detailed list of instruments, score ranges, and directional alignment is available in **Supplementary Table S2**. Where different scales were used to assess the same outcome (e.g., GAD-7, BAI, and STAI for anxiety), SMD was applied to enhance comparability. For each outcome, post-intervention values were extracted as the primary endpoint. If only change-from-baseline scores were available, they were converted according to Cochrane Handbook guidance. When studies lacked means or standard deviations, estimates were derived from medians, interquartile ranges, or p-values using standard approaches (19). In cases requiring correlation coefficients, a default value of 0.5 was applied, with sensitivity analyses conducted to test robustness. Authors were contacted when CItical data were missing; studies were excluded if data could not be retrieved.

For the pairwise meta-analyses, a random-effects model was employed to estimate intervention effects, with heterogeneity assessed using the I² statistic and Tau². Significant heterogeneity was indicated by an I² value greater than 50% or a p-value less than 0.1. Where necessary, sensitivity analyses were conducted, including the leave-one-out method and analyses restricted to studies at lower risk of bias, to examine the robustness of the primary conclusions. Furthermore, weighted least squares and variance-stabilizing transformations were applied as supplementary analytical techniques to mitigate the potential influence of small sample sizes or extreme values on the pooled estimates.

Subgroup analyses were further performed to explore the potential moderating effects of age, intervention type, and intervention duration on outcomes, aiming to identify how different population characteristics, intervention modalities, and timing might contribute to outcome variability. All subgroup variables were based on pre-specified clinical and theoretical rationales (e.g., age, intervention duration), with no *post hoc* exploratory splits conducted. We recognize that other factors, such as gender, severity of functional impairment, comorbid conditions (e.g., attention-deficit/hyperactivity disorder), and socioeconomic background, may also moderate intervention effects. However, due to the highly heterogeneous or incomplete reporting of these variables in the original studies, systematic subgroup or meta-regression analyses for these factors could not be performed in the present study.

The network meta-analysis was implemented using a frequentist framework and a multivariate random-effects model (20). The mvmeta command in Stata was used to model treatment effects on the log-odds scale, integrating both direct and indirect comparisons (21). A network geometry plot was constructed to illustrate the structure of available comparisons. Each node represented an intervention (with node size proportional to sample size), and edge thickness reflected the number of studies. A contribution plot was generated to quantify the relative influence of each direct comparison on the overall network estimates (22), helping to identify dominant pathways and structurally influential comparisons—particularly important in networks with sparse or uneven connections.

To assess inconsistency within the network, we applied three approaches:i) Loop-specific inconsistency was evaluated using the method of moments to test whether direct and indirect estimates within each closed loop significantly differed (p > 0.05 indicates consistency) (23);ii) Global inconsistency was assessed using the Wald test across the entire network (24);iii) Local inconsistency was examined using the node-splitting method, comparing direct and indirect estimates for each intervention contrast (25). When no significant inconsistency was detected, consistency models were used for inference. If inconsistency was present, results from inconsistency models were reported and subjected to further sensitivity analysis.

Intervention rankings were generated using the Surface Under the Cumulative Ranking Curve (SUCRA) method, which estimates the probability that each intervention is among the most effective (26). SUCRA values range from 0 (least effective) to 1 (most effective). Rankograms were constructed to visualize the probability distribution across all possible ranks for each intervention, with the area under each curve corresponding to the SUCRA value (**Supplementary Figures S1**-**S3**). These visualizations provide intuitive insights into the cumulative performance of interventions for anxiety, depression, and QoL.

To evaluate potential publication bias, we constructed network funnel plots and performed Egger’s test along with visual symmetry assessment. Asymmetry was interpreted in light of sample size and effect distribution to distinguish true bias from heterogeneity or small-study effects. The overall certainty of evidence was assessed using the GRADE approach, considering risk of bias, inconsistency, indirectness, imprecision, and publication bias. The certainty of each outcome was classified as high, moderate, low, or very low. GRADE ratings were assigned separately for direct, indirect, and network-level estimates. All visualizations (e.g., network plots, SUCRA curves, funnel plots) were generated using Stata and Review Manager 5.3. Definitions of key statistical terms and details of analytical methods are provided in **Supplementary Methods 3**.

## 3 Results

### 3.1 Literature search and trial selection

**Figure 1:**
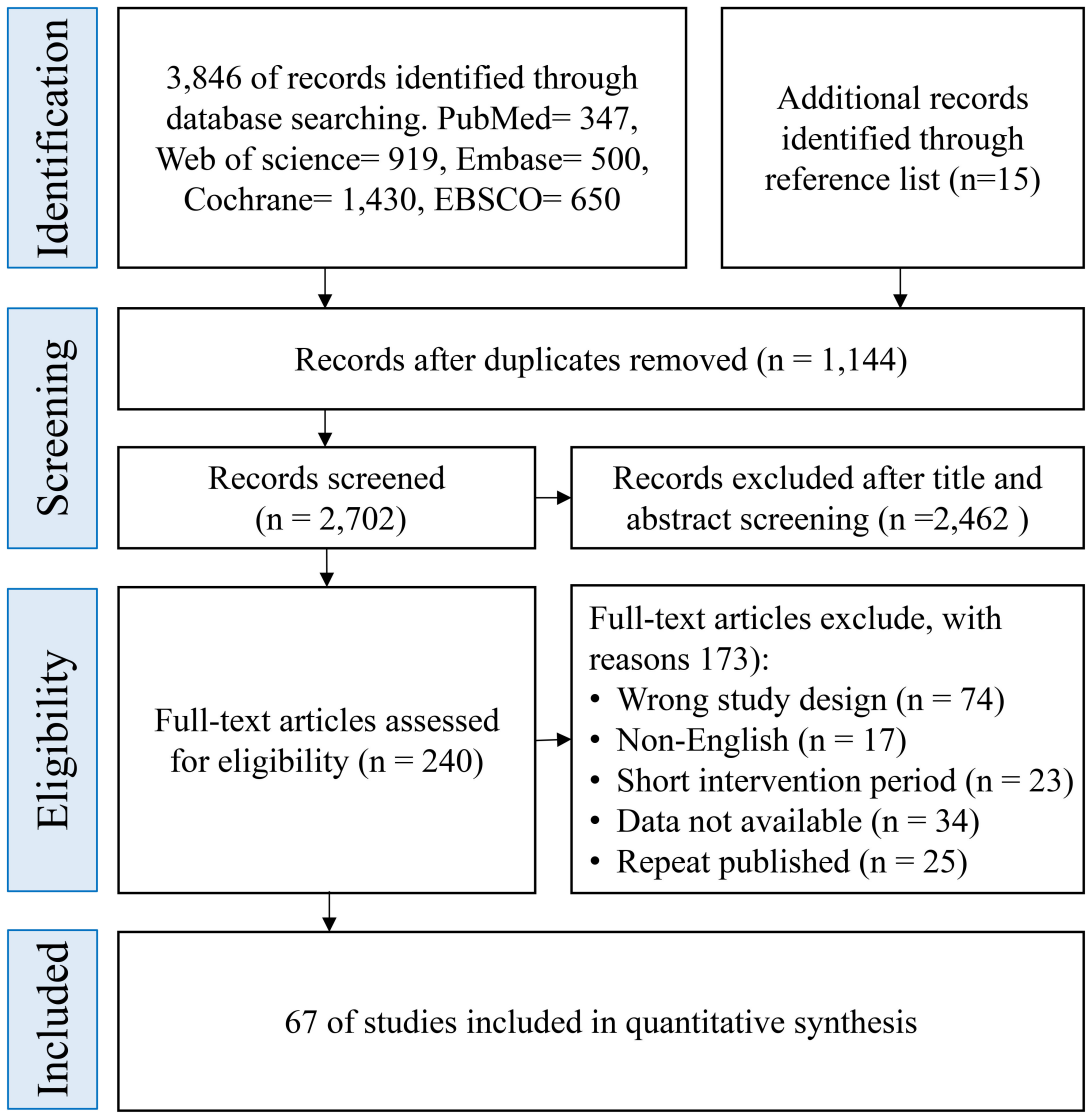
Preferred reporting items for systematic reviews and meta-analyses flow diagram of each stage of the study selection.

A total of 3,846 records were identified through electronic database searches. An additional 15 potentially eligible records were retrieved by screening the reference lists of included studies and relevant systematic reviews. All records were imported into EndNote X9 (Clarivate Analytics) for de-duplication. After removing duplicates, 2,702 unique records remained for title and abstract screening. During the initial screening, 2,462 records were excluded due to irrelevance in topic, intervention type, or study population. The remaining 240 full-text articles were assessed for eligibility. Of these, 173 were excluded based on the following reasons: inappropriate study design (n = 74), non-English publication (n = 17), insufficient or unclear intervention duration (n = 23), insufficient outcome data for extraction (n = 34), and duplicate publication or overlapping data sets (n = 25).Ultimately, 67 randomized controlled trials (RCTs) met all eligibility CIteria and were included in the quantitative synthesis. The complete process of study identification and selection is illustrated in the PRISMA flow diagram (**Figure 1**), which outlines the screening and inclusion pathway in detail.

### 3.2 Description of the included trials

A total of 67 randomized controlled trials (RCTs) were included, reporting 1,909 participants in intervention arms (control group data not included). These studies were geographically diverse: 36.9% were conducted in North America, 32.3% in Europe, 15.4% in Oceania, 13.8% in Asia, and 1.5% in Africa. The mean age of participants was 17.82 ± 10.82 years, covering children, adolescents, and adults, indicating cross-age applicability. Only 7.1% of participants were female, reflecting the male-dominant sampling pattern common in ASD research. Some trials enrolled only male or only female participants, while others included mixed-gender groups. Detailed characteristics of the included studies are provided in (**Supplementary Table S3**). Regarding functional levels, over 70% of the studies explicitly recruited individuals with high-functioning autism, typically defined by normal or near-normal IQ and at least some verbal communication ability. Several studies included individuals with moderate or mixed levels of severity; a few did not specify severity but reported standard diagnostic CIteria (e.g., DSM/ICD) or behavioral screening cutoffs (e.g., SCQ ≥ 11). A small number classified participants using DSM-5 severity levels (e.g., Level 1–2 or Level 2). Overall, the current evidence base is concentrated on higher-functioning ASD populations, indicating a need for further validation among lower-functioning or nonverbal individuals.

The interventions reported across included studies were categorized into eight groups: mindfulness-based interventions (MBI); cognitive behavioral therapy (CBT); behavioral and functional training (BEHAVE), including PEERS, STEPS, and structured social skills programs; physical activity (PHYS), such as dance, equine therapy, and football; sensory-based therapies (SENS), including olfactory stimulation and sensory integration; technology-assisted and family-based interventions (TAFI), including parent training and app-based delivery; other therapies (OTH), including drama therapy, animal-assisted therapy, and sleep management; and control conditions (CTRL), including waitlist, usual care, or psychoeducation. Each study evaluated at least one outcome related to anxiety, depression, or quality of life (QoL). Anxiety was commonly assessed using GAD-7, STAI, BAI, SCAS, ADIS-P, or PARS; depression was measured using instruments such as BDI, PHQ-9, DASS, or CDI; QoL was assessed using WHOQOL-BREF, PedsQL, QOLI, or CHU-9D. Most outcomes were assessed immediately post-intervention, while several studies reported follow-up periods ranging from 1 to 6 months.

Intervention durations ranged from 1 to 52 weeks, with a mean duration of 12.2 ± 7.4 weeks. Session frequency ranged from 1 to 5 times per week, with individual sessions typically lasting 45 to 180 minutes. Some interventions incorporated home-based practice or digital platforms to increase total training exposure. The average dropout rate was 13.6% in intervention groups and 11.2% in control groups. Sensory-based therapies (SENS, 7.8%) and TAFI (9.4%) showed relatively better adherence, whereas CBT (15.9%) and BEHAVE (17.2%) had higher attrition rates, suggesting that structural complexity, cognitive demand, or sustained engagement requirements may affect completion rates.

In summary, the included trials demonstrate substantial variability and representativeness in sample characteristics, intervention formats, and outcome assessments, supporting their inclusion in network-based comparisons and quantitative synthesis (**Supplementary Table S3**).

### 3.3 Results of risk of bias assessment

Detailed risk of bias assessments for all included studies are summarized in (**Supplementary Table S4**). Among the 67 included RCTs, 62 (92.5%) clearly reported their methods for sequence generation, indicating generally low risk of selection bias during the randomization process (**Supplementary Figure S1**). However, only 37 trials (55.2%) provided information on allocation concealment, suggesting a potential risk of predictability in group assignment for a substantial proportion of studies. Regarding blinding, 20 studies were rated as high risk for performance bias due to lack of participant and personnel blinding, especially in behavioral and psychological intervention settings (**Supplementary Figure S2**). For detection bias, 34 studies (50.7%) reported clear outcome assessor blinding. Most studies showed low risk of attrition bias, with 60 studies (89.6%) reporting minimal missing data and appropriate handling of dropouts. Selective reporting was well-controlled, with 58 trials (86.6%) showing no evidence of reporting bias. Other sources of bias were identified in several studies with small sample sizes (<10 participants per group), use of non-validated tools, or absence of intervention fidelity monitoring, leading to high or unclear risk judgments in those domains. Based on the overall assessment across seven ROB domains, 43 studies were classified as low risk, 19 as moderate risk, and 5 as high risk. In summary, the included trials demonstrated generally high methodological quality in randomization, outcome reporting, and data completeness. However, further improvements are warranted in areas such as blinding procedures, allocation concealment, and sample size adequacy.

### 3.4 Pairwise meta-analyses

#### 3.4.1 Anxiety

In the pairwise meta-analysis of anxiety outcomes, several interventions demonstrated statistically significant reductions in anxiety symptoms compared with control groups. These included cognitive behavioral therapy (SMD = –0.67, 95% CI [–0.94, –0.39]; I² = 93%), mindfulness-based interventions (SMD = –0.49, 95% CI [–0.62, –0.37]; I² = 71%), behavioral and functional training (SMD = –0.28, 95% CI [–0.45, –0.10]; I² = 45%), technology-assisted and family interventions (SMD = –0.25, 95% CI [–0.48, –0.02]; I² = 62%), and other therapies (SMD = –0.45, 95% CI [–0.69, –0.21]; I² = 0%).Physical activity (SMD = –0.52, 95% CI [–1.15, 0.11]) and sensory-based therapies (SMD = –0.16, 95% CI [–0.44, 0.12]) showed nonsignificant effects, as their credible intervals crossed zero. Between-study heterogeneity was substantial (I² range: 0% to 93%), potentially attributable to variations in sample characteristics, intervention implementation, or measurement tools. Detailed estimates are provided in (**Supplementary Figure S3**).

#### 3.4.2 Depression

CBT showed a statistically significant reduction in depressive symptoms compared to controls (SMD = –0.35, 95% CI [–0.61, –0.09]; I² = 6%). Other interventions also showed negative effect directions, but none reached statistical significance: mindfulness-based interventions (SMD = –0.10, 95% CI [–0.36, 0.16]; I² = 49%), behavioral and functional training (SMD = –0.21, 95% CI [–0.46, 0.05]; I² = 66%), technology-assisted and family interventions (SMD = –0.10, 95% CI [–0.48, 0.28]; I² = 75%), physical activity (SMD = –0.01, 95% CI [–0.63, 0.61]), and other therapies (SMD = –0.40, 95% CI [–0.81, 0.02]; I² = 0%).Although most interventions showed effects in the expected direction, the results were not statistically significant. Between-study heterogeneity ranged from 0% to 75%. Potential sources of heterogeneity are further discussed in the subgroup analysis. Detailed estimates are presented in (**Supplementary Figure S4**).

#### 3.4.3 Quality of life

Two interventions significantly improved quality of life outcomes: physical activity (SMD = 0.98, 95% CI [0.61, 1.35]; I² = 0%) and technology-assisted and family interventions (SMD = 0.72, 95% CI [0.43, 1.01]; I² = 87%). Other interventions showed positive but nonsignificant effects: mindfulness-based interventions (SMD = 0.24, 95% CI [–0.10, 0.58]; I² = 59%), CBT (SMD = 0.15, 95% CI [–0.06, 0.36]; I² = 41%), behavioral and functional training (SMD = 0.40, 95% CI [–0.26, 1.06]), and sensory-based therapies (SMD = 0.25, 95% CI [–0.25, 0.75]; I² = 65%).The pooled effect across all interventions was statistically significant (SMD = 0.41, 95% CI [0.27, 0.54]; I² = 71%), indicating a general positive trend in improving quality of life in individuals with ASD. Heterogeneity ranged from 0% to 87%, possibly related to differences in participant age, intervention duration, and outcome measures. Full results are shown in (**Supplementary Figure S5**).

### 3.5 Network meta-analysis

#### 3.5.1 Anxiety

**Figure 2:**
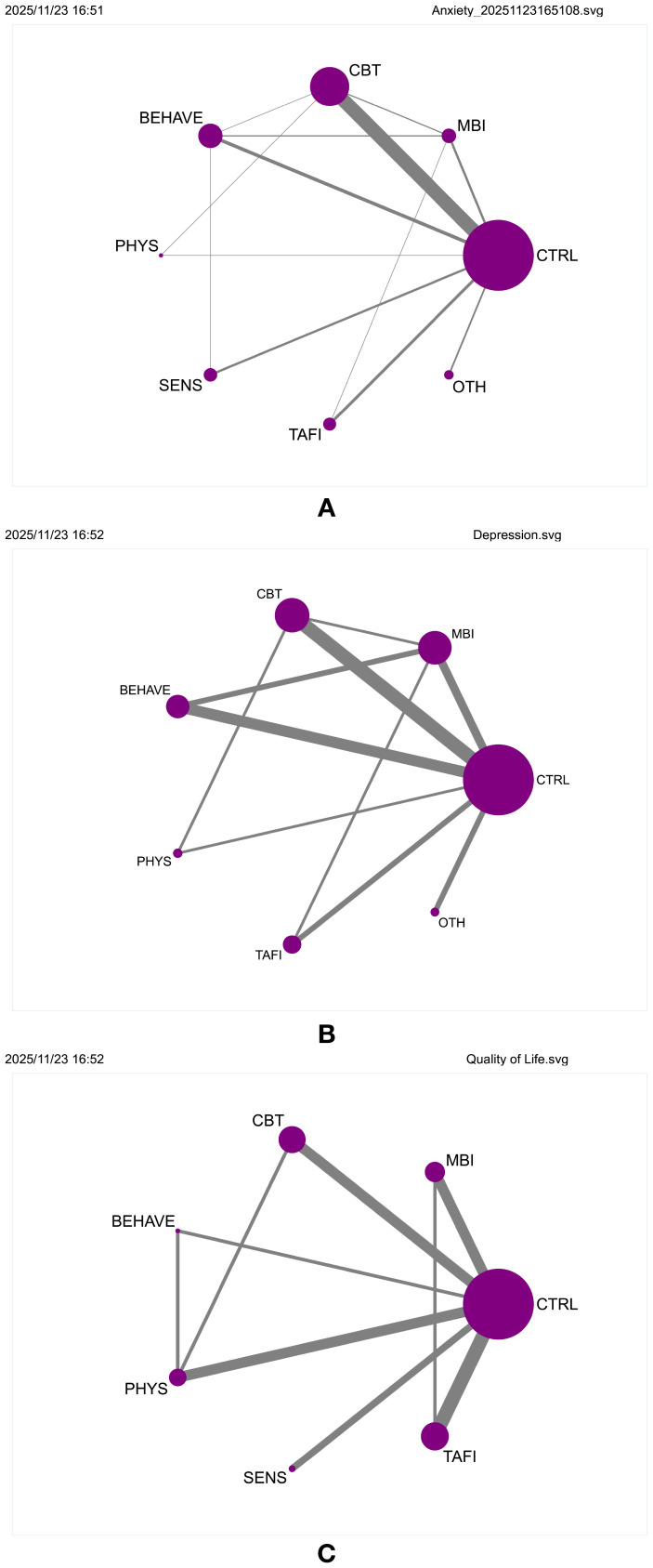
**(A)** Network geometry of comparisons for anxiety outcomes. **(B)** Network geometry of comparisons for depression outcomes. **(C)** Network geometry of comparisons for quality of life outcomes.

A frequentist random-effects model was used to conduct a network meta-analysis comparing the relative effectiveness of seven non-pharmacological interventions in alleviating anxiety symptoms among individuals with ASD. The network geometry is illustrated in (**Supplementary Figure S6**, **Figure 2A**), comprising 28 direct comparison links. MBI, CBT, BEHAVE, and CTRL formed the core comparison network, with CBT acting as the central hub with the most connections. Node size and edge thickness represent sample size and comparison strength, respectively, reflecting a “core–periphery” structure.

The contribution plot revealed that MBI *vs*. CTRL (100.0%) and CBT *vs*. CTRL (60.5%) had the highest influence on overall network estimates (**Supplementary Figure S7**). Consistency was confirmed through loop-specific, global, and local inconsistency tests (all p > 0.05), indicating a coherent network structure (**Supplementary Tables S5**-**S7**). The funnel plot showed approximate symmetry, and after removing two outlying small-sample studies, no substantial small-study effects or publication bias were detected (**Supplementary Figure S8**). Additional figures are provided in (**Supplementary Figures S9**, **S10**). Main effect estimates are presented in (**Table 1**, **Supplementary Table S14**). MBI showed the strongest effect in reducing anxiety symptoms (SMD = –1.13, 95% CI [–1.69, –0.57]), outperforming CBT (SMD = –0.65, 95% CI [–0.93, –0.36]), BEHAVE (SMD = –0.62, 95% CI [–1.09, –0.14]), and PHYS (SMD = –0.55, 95% CI [–1.55, –0.45]). While SENS, TAFI, and OTH also showed negative effects, their credible intervals crossed zero, indicating insufficient evidence for statistical significance. SUCRA rankings (**Supplementary Figure S11**) further supported these findings, with MBI ranked highest (94.3%), followed by CBT (64.2%), BEHAVE (60.6%), PHYS (54.0%), OTH (47.5%), and TAFI (42.1%). SENS ranked lowest among active interventions (28.2%), while the control condition showed the lowest SUCRA value overall (9.1%), confirming its baseline comparison status.

**Table 1:** Network meta-analysis matrix of anxiety, depression, and quality of life.

Outcome Measures								
Anxiety	MBI	0.49 (-0.10,1.07)	0.52 (-0.12,1.15)	0.58 (-0.55,1.72)	0.92 (0.08,1.75)	0.73 (-0.05,1.51)	0.68 (-0.30,1.66)	1.13 (0.57,1.69)
	-0.49 (-1.07,0.10)	CBT	0.03 (-0.50,0.56)	0.10 (-0.90,1.10)	0.43 (-0.27,1.13)	0.24 (-0.43,0.91)	0.20 (-0.66,1.05)	0.65 (0.36,0.93)
	-0.52 (-1.15,0.12)	-0.03 (-0.56,0.50)	BEHAVE	0.07 (-1.03,1.17)	0.40 (-0.33,1.13)	0.21 (-0.55,0.98)	0.17 (-0.77,1.10)	0.62 (0.14,1.09)
	-0.58 (-1.72,0.55)	-0.10 (-1.10,0.90)	-0.07 (-1.17,1.03)	PHYS	0.33 (-0.86,1.52)	0.15 (-1.02,1.32)	0.10 (-1.18,1.38)	0.55 (-0.45,1.55)
	-0.92(-1.75,-0.08)	-0.43 (-1.13,0.27)	-0.40 (-1.13,0.33)	-0.33 (-1.52,0.86)	SENS	-0.19 (-1.07,0.70)	-0.23 (-1.26,0.80)	0.21 (-0.43,0.86)
	-0.73 (-1.51,0.05)	-0.24 (-0.91,0.43)	-0.21 (-0.98,0.55)	-0.15 (-1.32,1.02)	0.19 (-0.70,1.07)	TAFI	-0.05 (-1.06,0.96)	0.40 (-0.21,1.01)
	-0.68 (-1.66,0.30)	-0.20 (-1.05,0.66)	-0.17 (-1.10,0.77)	-0.10 (-1.38,1.18)	0.23 (-0.80,1.26)	0.05 (-0.96,1.06)	OTH	0.45 (-0.36,1.25)
	-1.13(-1.69,-0.57)	-0.65 (-0.93,-0.36)	-0.62(-1.09,-0.14)	-0.55 (-1.55,0.45)	-0.21 (-0.86,0.43)	-0.40 (-1.01,0.21)	-0.45 (-1.25,0.36)	CTRL
Depression								
	MBI	-0.08 (-0.47,0.32)	-0.00 (-0.38,0.38)	0.34 (-0.29,0.97)	0.02 (-0.50,0.53)	-0.14 (-0.77,0.49)	0.26 (-0.07,0.59)	
	0.08 (-0.32,0.47)	CBT	0.08 (-0.36,0.51)	0.42 (-0.13,0.97)	0.09 (-0.46,0.64)	-0.07 (-0.68,0.55)	0.33 (0.03,0.64)	
	0.00 (-0.38,0.38)	-0.08 (-0.51,0.36)	BEHAVE	0.34 (-0.30,0.99)	0.02 (-0.55,0.58)	-0.14 (-0.78,0.50)	0.26 (-0.08,0.60)	
	-0.34 (-0.97,0.29)	-0.42 (-0.97,0.13)	-0.34 (-0.99,0.30)	PHYS	-0.33 (-1.06,0.40)	-0.49 (-1.26,0.29)	-0.08 (-0.64,0.47)	
	-0.02 (-0.53,0.50)	-0.09 (-0.64,0.46)	-0.02 (-0.58,0.55)	0.33 (-0.40,1.06)	TAFI	-0.16 (-0.88,0.56)	0.24 (-0.24,0.72)	
	0.14 (-0.49,0.77)	0.07 (-0.55,0.68)	0.14 (-0.50,0.78)	0.49 (-0.29,1.26)	0.16 (-0.56,0.88)	OTH	0.40 (-0.14,0.94)	
	-0.26 (-0.59,0.07)	-0.33 (-0.64,-0.03)	-0.26 (-0.60,0.08)	0.08 (-0.47,0.64)	-0.24 (-0.72,0.24)	-0.40 (-0.94,0.14)	CTRL	
Quality of Life								
	PHYS	-0.66 (-6.59,5.27)	-2.82(-11.41,5.7)	-3.34 (-9.52,2.84)	-4.15(-10.27,1.98)	-5.32(-9.71,-0.94)	-4.16 (-8.88,0.56)	
	0.66 (-5.27,6.59)	TAFI	-2.16(-10.44,6.1)	-2.68 (-9.78,4.42)	-3.48 (-9.13,2.16)	-4.66(-8.70,-0.62)	-3.50 (-8.88,1.88)	
	2.82(-5.78,11.41)	2.16 (-6.12,10.44)	SENS	-0.52 (-9.87,8.83)	-1.33 (-9.78,7.13)	-2.50 (-9.71,4.70)	-1.34 (-9.37,6.69)	
	3.34 (-2.84,9.52)	2.68 (-4.42,9.78)	0.52 (-8.83,9.87)	CBT	-0.81 (-8.08,6.46)	-1.98 (-7.84,3.87)	-0.82 (-7.39,5.74)	
	4.15(-1.98,10.27)	3.48 (-2.16,9.13)	1.33 (-7.13,9.78)	0.81 (-6.46,8.08)	MBI	-1.18 (-5.53,3.17)	-0.01 (-5.63,5.60)	
	5.32 (0.94,9.71)	4.66 (0.62,8.70)	2.50 (-4.70,9.71)	1.98 (-3.87,7.84)	1.18 (-3.17,5.53)	BEHAVE	1.16 (-2.39,4.72)	
	4.16 (-0.56,8.88)	3.50 (-1.88,8.88)	1.34 (-6.69,9.37)	0.82 (-5.74,7.39)	0.01 (-5.60,5.63)	-1.16 (-4.72,2.39)	CTRL	

Subgroup analyses revealed that interventions were more effective among adults (>18 years; SMD = –0.61, 95% CI [–0.82, –0.40]) than among children/adolescents (≤18 years; SMD = –0.38, 95% CI [–0.56, –0.20]), suggesting age may moderate intervention efficacy. By intervention type, MBI yielded the strongest effects (SMD = –0.85, 95% CI [–1.32, –0.38]), followed by CBT (SMD = –0.60), TAFI (SMD = –0.57), SENS (SMD = –0.52), and BEHAVE (SMD = –0.43). OTH showed the weakest effect (SMD = –0.33, 95% CI [–0.62, –0.04]).Duration-based subgroup analysis showed that medium-duration interventions (9–16 weeks) had the most pronounced effect (SMD = –0.59, 95% CI [–0.83, –0.35]), followed by short-term interventions (≤8 weeks; SMD = –0.54), while long-term interventions (>16 weeks) had slightly reduced effects (SMD = –0.47, 95% CI [–0.70, –0.23]). These findings suggest that moderate intervention durations may optimize outcomes. Relevant visualizations are presented in (**Supplementary Figures S12**–**S14**).

#### 3.5.2 Depression

To evaluate the comparative efficacy of various non-pharmacological interventions for depressive symptoms in individuals with autism spectrum disorder (ASD), a network meta-analysis was conducted using a frequentist random-effects model. Six intervention types were included: mindfulness-based interventions (MBI), cognitive behavioral therapy (CBT), behavioral and functional training (BEHAVE), physical activity (PHYS), technology-assisted and family-involved interventions (TAFI), and other therapies (OTH). The network structure involved 15 direct comparisons, with CBT *vs*. CTRL and MBI *vs*. CTRL serving as central links, indicating a concentrated evidence base (**Figure 2B**, **Supplementary Figure S15**). Contribution analysis showed that CBT *vs*. CTRL contributed most to overall effect estimation, followed by MBI *vs*. CTRL (**Supplementary Figure S16**). Consistency was supported by non-significant loop-specific, global, and local inconsistency tests (all p > 0.05; **Supplementary Tables S8**-**S10**). Funnel plots appeared symmetric, and exclusion of small-sample studies did not alter the overall pattern, suggesting minimal publication bias (**Supplementary Figure S17**). Additional effect displays are available (**Supplementary Figures S18**, **S19**).

Effect estimates (**Table 2**, **Supplementary Table S15**) indicated that CBT had the most robust and statistically significant effect in reducing depressive symptoms (SMD = –0.33; 95% CI [–0.64, –0.03]), being the only intervention with a credible interval not overlapping zero. Although MBI (SMD = –0.26; 95% CI [–0.59, 0.07]), BEHAVE (SMD = –0.26; 95% CI [–0.60, 0.08]), and TAFI (SMD = –0.24; 95% CI [–0.72, 0.24]) showed similar directions, their intervals crossed zero. PHYS showed a positive but negligible impact (SMD = 0.08), suggesting limited benefit. SUCRA rankings showed OTH (73.4%) and CBT (70.5%) ranked highest (**Supplementary Figure S20**); however, the estimate for OTH (SMD = –0.40; 95% CI [–0.94, 0.14]) was not statistically significant, warranting cautious interpretation.

**Table 2:** Ranking of non-pharmacological interventions in order of effectiveness.

Anxiety(49studies,N=2714)		Depression(20studies,N=1052)		Quality of life(17studies,N=861)	
Treatment	SUCRA	Treatment	SUCRA	Treatment	SUCRA
MBI	94.3	OTH	73.4	PHYS	84.3
CBT	64.2	CBT	70.5	TAFI	78.1
BEHAVE	60.6	BEHAVE	58.8	SENS	51.3
PHYS	54	MBI	58.2	BEHAVE	46.0
OTH	47.5	TAFI	55.3	MBI	36.7
TAFI	42.1	PHYS	17.3	CBT	35.7
SENS	28.2	CTRL	16.5	CTRL	18.0
CTRL	9.1	–	–	–	–

Subgroup analyses were conducted to explore potential effect modifiers. Age-related differences were minimal, with slightly greater effects observed among adults (SMD = 0.25; 95% CI [0.10, 0.40]) than adolescents (SMD = 0.24; 95% CI [0.02, 0.46]), and no significant between-group heterogeneity (p = 0.948). When classified by intervention type, MBI (SMD = 0.32; 95% CI [0.10, 0.54]) and CBT (SMD = 0.24; 95% CI [0.04, 0.44]) showed consistently positive effects. Although BEHAVE (SMD = 0.05), SENS (SMD = 0.32), and TAFI (SMD = 0.52) followed the same direction, none reached statistical significance. The ranking of OTH, despite its high SUCRA, was undermined by a wide credible interval. In terms of intervention duration, medium-term interventions (9–16 weeks) yielded the strongest effects (SMD = 0.32; 95% CI [0.13, 0.50]), followed by short-term (≤8 weeks; SMD = 0.19) and long-term (>16 weeks; SMD = 0.19; 95% CI [–0.03, 0.42]). No significant differences were observed across duration groups (p = 0.636), suggesting that pacing may affect accumulation and maintenance of benefits (**Supplementary Figures S21**, **S23**).

Overall, CBT demonstrated the most consistent and statistically significant antidepressant effect, with MBI also showing promise. BEHAVE and TAFI displayed potential but require further validation in large-scale trials. While SUCRA rankings inform prioritization, they should be interpreted in light of corresponding estimates and credible intervals. Future research should expand sample sizes, standardize intervention protocols, and enhance identification of moderating factors to enable more targeted and scalable interventions.

#### 3.5.3 Quality of life

To assess the effectiveness of non-pharmacological interventions on quality of life (QoL) in individuals with autism spectrum disorder (ASD), a frequentist random-effects model was employed to conduct a network meta-analysis involving six intervention types: mindfulness-based interventions (MBI), cognitive behavioral therapy (CBT), behavioral and functional training (BEHAVE), physical activity (PHYS), sensory therapy (SENS), and technology-assisted and family-involved interventions (TAFI). The network structure is shown in **Figure 2C** and **Supplementary Figure S24**, where all interventions were directly compared with the control group (CTRL), forming a tightly connected and evenly distributed network. The PHYS *vs*. CTRL (100.0) and TAFI *vs*. CTRL (67.4) comparisons contributed most to the overall effect estimation, followed closely by CBT (63.0) and MBI (60.3), suggesting a multi-hub, highly interconnected configuration (**Supplementary Figure S25**). All inconsistency tests, including loop-specific, global, and local, showed p-values > 0.05 (**Supplementary Tables S11**-**S13**), indicating good model consistency. The funnel plot appeared symmetric, with no significant risk of small-study effects or publication bias (**Supplementary Figure S26**).

Main effect estimates are presented in **Table 3** and **Supplementary Table S16**. As higher QoL scores indicate better outcomes, a positive SMD suggests an improvement. PHYS (SMD = 5.32; 95% CI [0.94, 9.71]) and TAFI (SMD = 4.66; 95% CI [0.62, 8.70]) significantly improved QoL, with credible intervals not crossing zero. While SENS (SMD = 2.50; 95% CI [–4.70, 9.71]), CBT (SMD = 1.98; 95% CI [–3.87, 7.84]), MBI (SMD = 1.18; 95% CI [–3.87, 7.84]), and BEHAVE (SMD = 1.16; 95% CI [–2.39, 4.72]) followed the same positive direction, their intervals overlapped zero and did not reach statistical significance. SUCRA rankings (**Supplementary Figure S27**) placed PHYS (84.3%) and TAFI (78.1%) at the top, followed by SENS (51.3%), BEHAVE (46.0%), MBI (36.7%), and CBT (35.7%), with CTRL ranking lowest (18.0%). The cumulative ranking plot (**Supplementary Figure S28**) further supported this pattern.

**Table 3:** Summary of core risk of bias indicators for included studies.

Bias domain	Risk proportion (n=67)	Key issue note	Reference
A	92.5% (62/67)	Good control of selection bias	**Supplementary Figure S1**
B	55.2% (37/67)	Nearly half of the studies have a risk of predictable grouping	**Supplementary Figure S1**
C	70.1% (47/67)	High difficulty in implementing blinding for behavioral/psychological interventions	**Supplementary Figure S2**
D	50.7% (34/67)	May affect the objectivity of emotional scale scores	**Supplementary Figure S2**
E	89.6% (60/67)	Good control of attrition bias	**Supplementary Table S4**
F	86.6%(58/67)	A few studies did not fully report quality of life outcomes	**Supplementary Table S4**
G	64.2% (43/67 at low risk)	19 studies at moderate risk, 5 studies at high risk	**Supplementary Figure S1**-**Supplementary Figure S2**, **Supplementary Table S4**

A: Random sequence generation; B: Allocation concealment; C: Blinding of participants and personnel D: Blinding of outcome assessors; E: Incomplete outcome; F: Selective outcome reporting; G: Other risks of bias.

To explore potential effect modifiers, three subgroup analyses were conducted. The age subgroup analysis (**Supplementary Figure S29**) revealed greater effects among adolescents (≤18 years; SMD = –0.59; 95% CI [–1.12, –0.05]) compared to adults (>18 years; SMD = –0.31; 95% CI [–0.60, –0.02]), with significant between-group heterogeneity (p = 0.000), suggesting enhanced responsiveness among younger populations. In the intervention-type subgroup (**Supplementary Figure S30**), PHYS (SMD = –1.02; 95% CI [–1.49, –0.54]) was the only statistically significant approach. Although MBI (SMD = –0.53) ranked second, its confidence interval crossed zero. CBT (SMD = –0.11), BEHAVE (SMD = –0.07), SENS (SMD = –0.43), and TAFI (SMD = –1.62) also demonstrated negative trends, though with limited statistical certainty. Regarding intervention duration, mid-term interventions (9–16 weeks) showed the greatest effect (SMD = –0.56; 95% CI [–0.90, –0.21]), followed by short-term (≤8 weeks; SMD = –0.27; 95% CI [–0.82, 0.27]) and long-term interventions (>16 weeks; SMD = –0.37; 95% CI [–0.92, 0.17]). Group differences did not reach statistical significance (p = 0.000), but the findings suggest 9–16 weeks may be the most suitable duration for improving QoL (**Supplementary Figures S30**–**S32**).

Overall, PHYS and TAFI demonstrated the highest effectiveness and robustness in enhancing QoL among individuals with ASD. While MBI, CBT, and BEHAVE also showed potential benefits, current evidence remains inconclusive. Future studies should focus on optimizing intervention pacing, particularly mid-term durations, and tailoring strategies to varying functional levels to advance the precision and evidence base of QoL-enhancing interventions.

#### 3.5.4 GRADE assessment

For the pairwise meta-analyses, a random-effects model was employed to estimate intervention effects, with heterogeneity assessed using the I² statistic and Tau². Significant heterogeneity was indicated by an I² value greater than 50% or a p-value less than 0.1. Where necessary, sensitivity analyses were conducted, including the leave-one-out method and analyses restricted to studies at lower risk of bias, to examine the robustness of the primary conclusions. Furthermore, weighted least squares and variance-stabilizing transformations were applied as supplementary analytical techniques to mitigate the potential influence of small sample sizes or extreme values on the pooled estimates.

Subgroup analyses were further performed to explore the potential moderating effects of age, intervention type, and intervention duration on outcomes, aiming to identify how different population characteristics, intervention modalities, and timing might contribute to outcome variability. All subgroup variables were based on pre-specified clinical and theoretical rationales (e.g., age, intervention duration), with no *post hoc* exploratory splits conducted.

We recognize that other factors, such as gender, severity of functional impairment, comorbid conditions (e.g., attention-deficit/hyperactivity disorder), and socioeconomic background, may also moderate intervention effects. However, due to the highly heterogeneous or incomplete reporting of these variables in the original studies, systematic subgroup or meta-regression analyses for these factors could not be performed in the present study (**Table 4**).

**Table 4:** Summary of GRADE evidence quality for core outcomes (key intervention comparisons).

Outcome	Key intervention comparison	Main reasons for downgrading	GRADE level
Anxiety	CTRL *vs* MBI	Study limitations+ publication bias	LOW
	CTRL *vs* CBT	Study limitations	MODERATE
Depression	CTRL *vs* CBT	Publication bias	MODERATE
	CTRL *vs* MBI	Study limitations + imprecision	LOW
Quality of Life	CTRL *vs* PHYS	Study limitations + indirectness	LOW
	CTRL *vs* TAFI	Imprecision	VERY LOW

## 4 Discussion

This study employed a network meta-analysis (NMA) to establish a probability-based framework for prioritizing interventions in clinical decision-making. Although the included interventions are heterogeneous in their mechanisms of action, they share the ultimate goal of improving anxiety, depressive symptoms, and quality of life in individuals with ASD. By synthesizing both direct and indirect comparison evidence, the NMA allowed for a quantitative assessment of the relative effects of various interventions on a unified scale and provided SUCRA rankings. For instance, mindfulness-based intervention (MBI) demonstrated the highest probability of being the optimal intervention for alleviating anxiety (SUCRA = 94.3%). It is crucial to emphasize that the SUCRA value represents the relative likelihood of an intervention being the best option, not an absolute determination of efficacy. When SUCRA values for different interventions are close and their effect size confidence intervals widely overlap, their therapeutic effects should be considered comparable. In such cases, the final clinical choice should integrate patient preferences, intervention feasibility, and cost-effectiveness. This probabilistic ranking system provides high-level, robust evidence-based guidance for optimizing clinical decisions within a complex evidence landscape.

### 4.1 Effects of non-pharmacological interventions on anxiety in individuals with ASD

This study demonstrates distinct differential effectiveness among non-pharmacological interventions for improving anxiety symptoms in individuals with ASD. Mindfulness-Based Intervention (MBI) demonstrated the most substantial advantage (SMD = -1.13). It enhances patients’ emotion regulation and awareness through practices such as meditation and mindful breathing (27), a mechanism also validated in child and adolescent populations (28). Cognitive Behavioral Therapy (CBT) likewise exhibited robust efficacy (SMD = -0.65), with its core components—such as cognitive restructuring and exposure therapy—effectively helping patients identify and modify negative thought patterns (29, 30).

Subgroup analyses further elucidated differentiated intervention characteristics. Notably, the effect of MBI was particularly pronounced in adult ASD patients, potentially attributable to their greater cognitive maturity and self-management capabilities, enabling them to derive more benefit from interventions requiring introspection and meta-cognitive engagement. In contrast, for children and adolescents whose cognitive abilities are still developing, the more structured nature of CBT may render it more readily acceptable and implementable. Furthermore, Behavioral and Functional Training (BEHAVE, SMD = -0.62) and Physical Activity (PHYS, SMD = -0.55) also demonstrated moderate effect sizes. The former may indirectly alleviate anxiety by enhancing social and adaptive skills (31, 32), while the latter likely modulates emotion through physiological mechanisms (33). Regarding intervention duration, medium-term interventions lasting 9–16 weeks yielded the optimal benefit, suggesting that sufficient duration is crucial for intervention effectiveness, whereas overly extended periods may lead to fatigue and decreased adherence (32).

In comparison, the effects of Sensory Therapy (SENS) and Technology-Assisted and Family Intervention (TAFI) did not reach statistical significance in this study. This may be related to the considerable heterogeneity in intervention protocols and the unclear identification of core active components (34, 35), necessitating further validation through standardized protocols and component analyses in future research.

In summary, for targeting anxiety symptoms in ASD, MBI and CBT should be prioritized as recommended strategies. However, clinical selection must consider the patient’s age and developmental stage. MBI may hold a greater advantage for adults and high-functioning adolescents, whereas CBT or BEHAVE might represent more pragmatic choices for children, adolescents, or individuals requiring structured support. Future research should focus on optimizing personalized intervention pathways for different patient subgroups.

### 4.2 Effects of non-pharmacological interventions on depression in individuals with ASD

This study indicates that for depressive symptoms in individuals with ASD, Cognitive Behavioral Therapy (CBT) demonstrates the most clear-cut and robust efficacy (SMD = -0.33), being the only intervention where the effect size reached statistical significance. CBT effectively improves emotion management and social adaptation in ASD individuals by helping them identify and restructure negative cognitions, and learn behavioral activation and problem-solving strategies (36). Its delivery formats are flexible; for instance, both school-based group CBT and internet-delivered CBT (iCBT) have been shown to significantly reduce depressive symptoms while enhancing treatment accessibility and family involvement (37, 38).

Subgroup analyses provided important clues for precision intervention. This study found that CBT yielded consistent benefits across different age groups, being particularly acceptable and implementable for adolescents in a critical period of psychological development due to its structured nature. In contrast, although the overall effect of Mindfulness-Based Intervention (MBI) was not statistically significant (SMD = -0.26), it showed a positive improving trend, with greater potential observed particularly in adult ASD patients. This may be because adults generally possess more mature metacognitive and introspective abilities, enabling them to benefit more from MBI, which emphasizes awareness and non-judgmental acceptance (39, 40). Technology-Assisted and Family Intervention (TAFI) also demonstrated application value (SMD = -0.24). By utilizing digital platforms (e.g., interactive games, apps) and family support systems, TAFI enhances intervention engagement and sustainability, contributing positively to improving emotional state (41).

Notably, Physical Activity (PHYS) did not show a significant antidepressant effect in this study, which contrasts with some previous findings (42, 43). This discrepancy might be related to the generally shorter intervention durations of the included PHYS studies, limited sample sizes, or the possibility that its mechanism of action focuses more on anxiety reduction and quality of life improvement rather than directly targeting core depressive symptoms. The effects of Behavioral and Functional Training (BEHAVE) and Other Therapies (OTH) remained inconclusive, suggesting that their active components and intervention standardization need enhancement (44).

In summary, CBT should be the first-line strategy for alleviating depressive symptoms in ASD. Clinical decision-making should consider the patient’s age and cognitive profile: CBT should be prioritized for adolescents and individuals requiring structured support; for adults with relatively preserved cognitive function, MBI or TAFI could be considered as supplementary or alternative options. Future research needs to clarify the effects of different interventions on specific depressive subtypes (e.g., with comorbid anxiety, with anhedonia) to advance truly individualized treatment.

### 4.3 Effects of non-pharmacological interventions on quality of life in individuals with ASD

Quality of life (QoL) serves as a multidimensional composite indicator for evaluating intervention outcomes in ASD. This study found that in improving QoL, Physical Activity (PHYS) and Technology-Assisted and Family Intervention (TAFI) demonstrated unique value distinct from interventions targeting emotional symptoms. PHYS showed the optimal effect (SMD = 5.32), comprehensively enhancing life experience across physiological, psychological, and social functional dimensions through improved motor coordination, sensory integration, and facilitated social interaction within structured environments (45, 46). For instance, animal-assisted activities such as therapeutic horseback riding not only improved physical function but also significantly enhanced patients’ self-efficacy and well-being by establishing emotional connections (47).

Subgroup analyses suggest that QoL interventions require attention to developmental stage appropriateness. As another highly effective strategy (SMD = 4.66), the core advantage of TAFI lies in generating broad benefits by empowering the family system. Online platforms and parent training not only increase intervention accessibility but also, by improving parent-child interaction and family atmosphere, provide patients with a continuous and stable support environment, thereby exerting profound positive impacts on QoL (48, 49). It is noteworthy that such interventions were particularly effective in the child and adolescent population, potentially related to this group’s higher dependence on the family system.

In contrast, although Mindfulness-Based Intervention (MBI) can improve subjective well-being by enhancing emotion regulation and stress tolerance (39), and Cognitive Behavioral Therapy (CBT) may potentially enhance social function by improving social cognition (50), neither achieved a statistically significant improvement in overall QoL in this study. This may reflect the comprehensive nature of QoL measurement: the effects of interventions directly targeting symptom reduction may not fully translate into patient-reported overall life quality improvement. The uncertain effects of Sensory Therapy (SENS) and Behavioral and Functional Training (BEHAVE) might be related to insufficient assessment of the daily impact of sensory processing in current research, as well as limitations in the specificity of QoL scales for the ASD population (51).

In summary, improving the quality of life of individuals with ASD should prioritize multidimensional, ecological intervention strategies. PHYS and TAFI represent the most effective choices due to their ability to be directly embedded into life contexts and simultaneously enhance individual capabilities and the support environment. Future research should focus on developing more sensitive, ASD-specific QoL scales and explore synergistic models that integrate symptom-targeted interventions (e.g., CBT, MBI) with broader quality of life interventions (e.g., PHYS, TAFI).

### 4.4 Limitations

Despite its strengths, this study has several limitations that should be considered when interpreting the results. First, as the included samples primarily consisted of high-functioning male individuals with ASD, the generalizability of our findings to females, non-verbal individuals, or those with co-occurring intellectual disability is limited and warrants future investigation. Second, some intervention categories (e.g., Sensory Therapy [SENS] and Other Therapies [OTH]) were supported by only one or two studies, which may affect the robustness of their effect estimates. Third, although we used standardized mean differences (SMDs) to integrate data from different outcome scales, the heterogeneity in assessment tools for anxiety, depression, and quality of life may still impact the comparability and precision of the findings. Fourth, as the included samples primarily consisted of high-functioning individuals with ASD, we were unable to conduct moderator analyses for variables such as gender, comorbid ADHD, and socioeconomic status, primarily due to insufficient data completeness. Specifically, females constituted only 7.1% of the total sample, precluding meaningful gender-based subgroup analysis. Regarding ADHD comorbidity and socioeconomic variables, only 12 studies (17.9%) reported ADHD co-occurrence, and only 8 studies (11.9%) mentioned household income levels, resulting in inadequate data coverage to control for the confounding effects of these variables. Finally, our analytical approach required the synthesis of interventions into broad categories. Although this was methodologically necessary, it comes at the cost of obscuring the effects of individual therapeutic components. Future applications of methodologies such as component network meta-analysis would be valuable for disentangling the effective elements within multimodal interventions like TAFI and OTH.

## 5 Conclusions and implications

This network meta-analysis systematically evaluated the relative effectiveness of seven categories of non-pharmacological interventions for improving anxiety, depression, and quality of life in individuals with Autism Spectrum Disorder. The results demonstrate that Mindfulness-Based Intervention (MBI) holds the strongest advantage for alleviating anxiety symptoms (SUCRA = 94.3%), Cognitive Behavioral Therapy (CBT) provides the most stable improvement for depressive symptoms (SUCRA = 70.5%), while Physical Activity (PHYS) and Technology-Assisted and Family Intervention (TAFI) show the most significant effects on enhancing quality of life (SUCRAs of 84.3% and 78.1%, respectively).

Based on subgroup analyses, we propose clinically actionable intervention pathways: MBI should be prioritized for adult patients with co-occurring anxiety symptoms; CBT should be the primary choice across all age groups for depressive symptoms; and when the goal is enhancing quality of life, PHYS and TAFI demonstrate the greatest potential, with the latter being particularly suitable for the child and adolescent population. All intervention programs are recommended to last for at least 8 weeks, with a medium duration of 9–16 weeks yielding optimal benefits.

This study provides a clear decision-making framework for clinical practice, supporting the development of individualized intervention strategies based on the three dimensions of symptom targeting, population characteristics, and resource availability. Future research should focus on delineating the core components of interventions and systematically investigating key covariates to advance non-pharmacological interventions for ASD towards a higher level of precision.

## Data Availability

The original contributions presented in the study are included in the article/**Supplementary Material**. Further inquiries can be directed to the corresponding author.

## References

[1] Vahia VN . Diagnostic and statistical manual of mental disorders 5: A quick glance. Indian J Psychiatry. (2013) 55:220–3. doi: 10.4103/0019-5545.117131, PMID: 24082241 PMC3777342

[2] Centers for Disease Control and Prevention (CDC) . Data and statistics on autism spectrum disorder (2023). National Center on Birth Defects and Developmental Disabilities. Available online at: https://www.cdc.gov/ncbddd/autism/data.html (Accessed June 6, 2025).

[3] China National Radio . China Autism Education and Rehabilitation Industry Development Report (2024). CNR. Available online at: https://edu.cnr.cn/list/20240402/t20240402_526650066.shtml.

[4] Lai MC Kassee C Besney R Bonato S Hull L Mandy W . Prevalence of co-occurring mental health diagnoses in the autism population: A systematic review and meta-analysis. Lancet Psychiatry. (2019) 6:819–29. doi: 10.1016/S2215-0366(19)30289-5, PMID: 31447415

[5] Hollocks MJ Lerh JW Magiati I Meiser-Stedman R Brugha TS . Anxiety and depression in adults with autism spectrum disorder: A systematic review and meta-analysis. psychol Med. (2019) 49:559–72. doi: 10.1017/S0033291718002283, PMID: 30178724

[6] Cassidy S Ropar D Mitchell P Chapman P . Can adults with autism spectrum disorders infer what happened to someone from their emotional response? Autism Research (2014) 7(1):112–23. doi: 10.1002/aur.1351, PMID: 24307231

[7] Joshi G Wilens TE . Pharmacotherapy of attention-deficit/hyperactivity disorder in individuals with autism spectrum disorder. Child and Adolescent Psychiatric Clinics of North America. (2022) 31(3):449–68. doi: 10.1016/j.chc.2022.03.012, PMID: 35697395

[8] Lord C Charman T Havdahl A Carbone P Anagnostou E Boyd B . The Lancet Commission on the future of care and clinical research in autism. Lancet. (2022) 399:271–334. doi: 10.1016/S0140-6736(21)01541-5, PMID: 34883054

[9] Hume K Steinbrenner JR Odom SL Morin KL Nowell SW Tomaszewski B . Evidence-based practices for children, youth, and young adults with autism: Third generation review. J Autism Dev Disord. (2021) 51:4013–30. doi: 10.1007/s10803-021-04950-x, PMID: 33449225 PMC8510990

[10] Sukhodolsky DG Bloch MH Panza KE Reichow B . Cognitive-behavioral therapy for anxiety in children with high-functioning autism: A meta-analysis. Pediatrics. (2013) 132:e1341–50. doi: 10.1542/peds.2013-1193, PMID: 24167175 PMC3813396

[11] Spek AA Van Ham NC Nyklíček I . Mindfulness-based therapy in adults with an autism spectrum disorder: A randomized controlled trial. Res Dev Disabil. (2013) 34:246–53. doi: 10.1016/j.ridd.2012.08.009, PMID: 22964266

[12] Jiang X Song M Qin W Xu X Yuan Q . Nonpharmaceutical therapy for autism spectrum disorder: a protocol for systematic review and network meta-analysis. Medicine. (2022) 101:e28811. doi: 10.1097/MD.0000000000028811, PMID: 35363171 PMC9281987

[13] Hutton B Salanti G Caldwell DM Chaimani A Schmid CH Cameron C . The PRISMA extension statement for reporting of systematic reviews incorporating network meta-analyses of health care interventions: Checklist and explanations. Ann Internal Med. (2015) 162:777–84. doi: 10.7326/M14-2385, PMID: 26030634

[14] Zeng G Niu J Zhu K Li F Li L Gao K . Effects of non-pharmacological interventions on depressive and anxiety symptoms in pregnant women: A systematic review and network meta-analysis. EClinicalMedicine. (2025) 79:101255. doi: 10.1016/j.eclinm.2024.101255, PMID: 39802308 PMC11718295

[15] Catalá-López F Hutton B Núñez-Beltrán A Page MJ Ridao M Macías Saint-Gerons D . The pharmacological and non-pharmacological treatment of attention deficit hyperactivity disorder in children and adolescents: A systematic review with network meta-analyses of randomised trials. PloS One. (2017) 12:e0180355. doi: 10.1371/journal.pone.0180355, PMID: 28700715 PMC5507500

[16] Luo D Wan X Liu J Tong T . Optimally estimating the sample mean from the sample size, median, mid-range, and/or mid-quartile range. Stat Methods Med Res. (2018) 27:1785–805. doi: 10.1177/0962280216669183, PMID: 27683581

[17] Wan X Wang W Liu J Tong T . Estimating the sample mean and standard deviation from the sample size, median, range and/or interquartile range. BMC Med Res Method. (2014) 14:135. doi: 10.1186/1471-2288-14-135, PMID: 25524443 PMC4383202

[18] Sterne JA Savović J Page MJ Elbers RG Blencowe NS Boutron I . RoB 2: A revised tool for assessing risk of bias in randomised trials. BMJ. (2019) 366:l4898. doi: 10.1136/bmj.l4898, PMID: 31462531

[19] Hozo SP Djulbegovic B Hozo I . Estimating the mean and variance from the median, range, and the size of a sample. BMC Med Res Method. (2005) 5:13. doi: 10.1186/1471-2288-5-13, PMID: 15840177 PMC1097734

[20] Salanti G . Indirect and mixed-treatment comparison, network, or multiple-treatments meta-analysis: Many names, many benefits, many concerns for the next generation evidence synthesis tool. Res Synthesis Methods. (2012) 3:80–97. doi: 10.1002/jrsm.1037, PMID: 26062083

[21] Bucher HC Guyatt GH Griffith LE Walter SD . The results of direct and indirect treatment comparisons in meta-analysis of randomized controlled trials. J Clin Epidemiol. (1997) 50:683–91. doi: 10.1016/S0895-4356(97)00049-8, PMID: 9250266

[22] Papakonstantinou T Nikolakopoulou A Rücker G Chaimani A Schwarzer G Egger M . Estimating the contribution of studies in network meta-analysis: Paths, flows and streams. F1000Research. (2018) 7:610. doi: 10.12688/f1000research.15070.1, PMID: 30338058 PMC6148216

[23] Rücker G Schwarzer G . Reduce dimension or reduce weights? Comparing two approaches to multi-arm studies in network meta-analysis. Stat Med. (2015) 33:4353–69. doi: 10.1002/sim.6308, PMID: 24942211

[24] Dias S Welton NJ Caldwell DM Ades AE . Checking consistency in mixed treatment comparison meta-analysis. Stat Med. (2010) 29:932–44. doi: 10.1002/sim.3767, PMID: 20213715

[25] White IR Barrett JK Jackson D Higgins JP . Consistency and inconsistency in network meta-analysis: Model estimation using multivariate meta-regression. Res Synthesis Methods. (2012) 3:111–25. doi: 10.1002/jrsm.1045, PMID: 26062085 PMC4433771

[26] Salanti G Ades AE Ioannidis JPA . Graphical methods and numerical summaries for presenting results from multiple-treatment meta-analysis: An overview and tutorial. J Clin Epidemiol. (2011) 64:163–71. doi: 10.1016/j.jclinepi.2010.03.016, PMID: 20688472

[27] Simione L Frolli A Sciattella F Chiarella SG . Mindfulness-based interventions for people with autism spectrum disorder: A systematic literature review. Brain Sci. (2024) 14:1001. doi: 10.3390/brainsci14101001, PMID: 39452015 PMC11506216

[28] Hwang YS Kearney P Klieve H Lang W Roberts J . Cultivating mind: Mindfulness interventions for children with autism spectrum disorder and problem behaviours, and their mothers. J Child Family Stud. (2015) 24:3093–106. doi: 10.1007/s10826-015-0114-x

[29] Perihan C Burke M Bowman-Perrott L Bicer A Gallup J Thompson J . Effects of cognitive behavioral therapy for reducing anxiety in children with high functioning ASD: A systematic review and meta-analysis. J Autism Dev Disord. (2020) 50:1958–72. doi: 10.1007/s10803-019-03948-2, PMID: 30810842

[30] Ung D Selles R Small BJ Storch EA . A systematic review and meta-analysis of cognitive-behavioral therapy for anxiety in youth with high-functioning autism spectrum disorders. Child Psychiatry Hum Dev. (2015) 46:533–47. doi: 10.1007/s10578-014-0494-y, PMID: 25246292

[31] Reaven J Moody EJ Grofer Klinger L Keefer A Duncan A O’Kelley S . Training clinicians to deliver group CBT to manage anxiety in youth with ASD: Results of a multisite trial. J Consulting Clin Psychol. (2018) 86:205–17. doi: 10.1037/ccp0000276, PMID: 29504790 PMC5841598

[32] Spain D Sin J Linder KB McMahon J Happé F . Social anxiety in autism spectrum disorder: A systematic review. Res Autism Spectr Disord. (2018) 52:51–68. doi: 10.1016/j.rasd.2018.04.007

[33] Healy S Nacario A Braithwaite RE Hopper C . The effect of physical activity interventions on youth with autism spectrum disorder: A meta-analysis. Autism Res. (2018) 11:818–33. doi: 10.1002/aur.1955, PMID: 29693781

[34] Case-Smith J Weaver LL Fristad MA . A systematic review of sensory processing interventions for children with autism spectrum disorders. Autism. (2015) 19:133–48. doi: 10.1177/1362361313517762, PMID: 24477447

[35] Boyd BA Hume K McBee MT Alessandri M Gutierrez A Johnson L . Comparative efficacy of LEAP, TEACCH and non-model-specific special education programs for preschoolers with autism spectrum disorders. J Autism Dev Disord. (2014) 44:366–80. doi: 10.1007/s10803-013-1877-9, PMID: 23812661

[36] Mazefsky CA White SW . Emotion regulation: Concepts and practice in autism spectrum disorder. Child Adolesc Psychiatr Clinics North America. (2013) 23:15–24. doi: 10.1016/j.chc.2013.08.002, PMID: 24231164 PMC3830422

[37] Spaargaren KL Begeer SM Greaves-Lord K Riper H Van Straten A . Protocol of a randomized controlled trial into guided internet-delivered cognitive behavioral therapy for insomnia in autistic adults (i-Sleep Autism). Contemp Clin Trials. (2024) 146:107704. doi: 10.1016/j.cct.2024.107704, PMID: 39357740

[38] Luxford S Hadwin JA Kovshoff H . Evaluating the effectiveness of a school-based cognitive behavioural therapy intervention for anxiety in adolescents diagnosed with autism spectrum disorder. J Autism Dev Disord. (2017) 47:3896–908. doi: 10.1007/s10803-016-3007-4, PMID: 27440250 PMC5676836

[39] Conner CM White SW . Brief report: Feasibility and preliminary efficacy of individual mindfulness therapy for adults with autism spectrum disorder. J Autism Dev Disord. (2018) 48:290–300. doi: 10.1007/s10803-017-3316-y, PMID: 28921306

[40] Cachia RL Anderson A Moore DW . Mindfulness in individuals with autism spectrum disorder: A systematic review and narrative analysis. Rev J Autism Dev Disord. (2016) 3:165–78. doi: 10.1007/s40489-015-0064-x

[41] Odom SL Thompson JL Hedges S Boyd BA Dykstra JR Duda MA . Technology-aided interventions and instruction for adolescents with autism spectrum disorder. J Autism Dev Disord. (2015) 45:3805–19. doi: 10.1007/s10803-014-2323-z, PMID: 25468409

[42] Pan CY Tsai CL Chu CH Sung MC Huang CY Ma WY . Effects of physical exercise intervention on motor skills and executive functions in children with ADHD: A pilot study. J Attention Disord. (2019) 23:384–97. doi: 10.1177/1087054716687530, PMID: 25646023

[43] Güeita-Rodríguez J Ogonowska-Slodownik A Morgulec-Adamowicz N Martín-Prades ML Cuenca-Zaldívar JN Palacios-Ceña D . Effects of aquatic therapy for children with autism spectrum disorder on social competence and quality of life: A mixed methods study. Int J Environ Res Public Health. (2021) 18:3126. doi: 10.3390/ijerph18063126, PMID: 33803581 PMC8002945

[44] Davis TN Scalzo R Butler E Stauffer M Farah YN Perez S . Animal-assisted interventions for children with autism spectrum disorder: A systematic review. Educ Training Autism Dev Disabil. (2015) 50:316–29. doi: 10.1177/215416471505000307

[45] Bass MM Duchowny CA Llabre MM . The effect of therapeutic horseback riding on social functioning in children with autism. J Autism Dev Disord. (2009) 39:1261–7. doi: 10.1007/s10803-009-0734-3, PMID: 19350376

[46] Greco G De Ronzi R . Effect of Karate training on social, emotional, and executive functioning in children with autism spectrum disorder. J Phys Educ Sport. (2020) 20:1637–45. doi: 10.7752/jpes.2020.04220

[47] Zhao M Chen S You Y Wang Y Zhang Y . Effects of a therapeutic horseback riding program on social interaction and communication in children with autism. Int J Environ Res Public Health. (2021) 18:2656. doi: 10.3390/ijerph18052656, PMID: 33800787 PMC7967314

[48] Althoff CE Dammann CP Hope SJ Ausderau KK . Parent-mediated interventions for children with autism spectrum disorder: A systematic review. Am J Occup Ther. (2019) 73:7303205010p1–7303205010p13. doi: 10.5014/ajot.2019.030932, PMID: 31120831

[49] Estes A Swain DM MacDuffie KE . The effects of early autism intervention on parents and family adaptive functioning. Pediatr Med (Hong Kong China). (2019) 2:21. doi: 10.21037/pm.2019.07.03 PMC6776235 31583390

[50] Sung M Ooi YP Goh TJ Pathy P Fung DSS Ang RP . Effects of cognitive-behavioral therapy on anxiety in children with autism spectrum disorders: a randomized controlled trial. Child Psychiatry Hum Dev. (2011) 42(6):634–49. doi: 10.1007/s10578-011-0238-1, PMID: 21660428

[51] Schaaf RC Benevides TW Kelly D Mailloux-Maggio Z . Occupational therapy and sensory integration for children with autism: A feasibility, safety, acceptability and fidelity study. Autism. (2012) 16:321–7. doi: 10.1177/1362361311435157, PMID: 22318118

