# Supplementary material for: Comparative effectiveness of non-pharmacological interventions for anxiety, depression, and quality of life in individuals with autism spectrum disorder: A systematic review and network meta-analysis
Source: Front Psychiatry. 2025 Dec 2;16:1660412. doi: 10.3389/fpsyt.2025.1660412 (PMC12706584; doi:10.3389/fpsyt.2025.1660412)
Supplement: Supplementary file 1 [file SupplementaryFile1.pdf]

# SUPPLEMENTARY MATERIAL

## Table of Contents

**Supplementary Methods 1 –** Database search strategy.

**Supplementary Methods 2 –** Conversion formula for standard deviation (SD).

**Supplementary Methods 3 –** Explanation of technical terms.

**Supplementary Methods 4 –** Inclusion in the list of studies.

**Supplementary Table S1 –** Definition of Interventions and Controls.

**Supplementary Table S2 –** Anxiety, Depression and Quality of Life rating scale.

**Supplementary Table S3 –** Study characteristics of the included studies.

**Supplementary Table S4 –** Risk of bias table of included studies.

**Supplementary Table S5 –** Anxiety: Loop Inconsistency Test.

**Supplementary Table S6 –** Anxiety: Global Inconsistency Test.

**Supplementary Table S7 –** Anxiety: Local Inconsistency Test.

**Supplementary Table S8 –** Depression: Loop Inconsistency Test.

**Supplementary Table S9 –** Depression: Global Inconsistency Test.

**Supplementary Table S10 –** Depression: Local Inconsistency Test.

**Supplementary Table S11 –** Quality of Life: Loop Inconsistency Test.

**Supplementary Table S12 –** Quality of Life: Global Inconsistency Test.

**Supplementary Table S13 –** Quality of Life: Local Inconsistency Test.

**Supplementary Table S14 –** Anxiety: league table.

**Supplementary Table S15 –** Depression: league table.

**Supplementary Table S16 –** Quality of Life: league table.

**Supplementary Table S17** – Table of reasons for downgrading.

**Supplementary Figure S1** – Risk of bias graph.

**Supplementary Figure S2** – Risk of bias summary.

**Supplementary Figure S3 –** Anxiety: Pairwise meta-analysis.

**Supplementary Figure S4 –** Depression: Pairwise meta-analysis.

**Supplementary Figure S5 –** Quality of Life: Pairwise meta-analysis.

**Supplementary Figure S6 –** Anxiety: network evidence plot.

**Supplementary Figure S7** – Anxiety:Contribution plot of the network.

**Supplementary Figure S8 –** Anxiety: comparison-adjusted funnel plot.

**Supplementary Figure S9 –** Anxiety: network forest.

**Supplementary Figure S10** – Anxiety: Forest plot of pairwise com parisons.

**Supplementary Figure S11** – Anxiety: cumulative probability plot .

**Supplementary Figure S12** – Anxiety: Subgroup analysis by gender.

**Supplementary Figure S13** – Anxiety: Subgroup analysis by interv ention type.

**Supplementary Figure S14** – Anxiety: Subgroup analysis by intervention duration.

**Supplementary Figure S15 –** Depression: network evidence plot.

**Supplementary Figure S16** – Depression:Contribution plot of the network.

**Supplementary Figure S17 –** Depression: comparison-adjusted funnel plot.

**Supplementary Figure S18 –** Depression: network forest.

**Supplementary Figure S19** – Depression: Forest plot of pairwise c omparisons.

**Supplementary Figure S20 –** Depression: cumulative probability plot .

**Supplementary Figure S21** – Depression: Subgroup analysis by gender.

**Supplementary Figure S22** – Depression: Subgroup analysis by int ervention type.

**Supplementary Figure S23** – Depression: Subgroup analysis by intervention duration.

**Supplementary Figure S24 –** Quality of Life: network evidence plot.

**Supplementary Figure S25** – Quality of Life:Contribution plot of the network.

**Supplementary Figure S26 –** Quality of Life: comparison-adjusted funnel plot.

**Supplementary Figure S27 –** Quality of Life: network forest.

**Supplementary Figure S28** – Quality of Life: Forest plot of pairwise comparisons.

**Supplementary Figure S29** – Quality of Life: cumulative probability plot .

**Supplementary Figure S30** – Quality of Life: Subgroup analysis by gender.

**Supplementary Figure S31** – Quality of Life: Subgroup analysis by intervention type.

**Supplementary Figure S32** – Quality of Life: Subgroup analysis by intervention duration.

**Supplementary Figure S33** – Anxiety: Risk of bias network for pairwise comparisons.

**Supplementary Figure S34** – Anxiety: Contribution of low, moderate, and high RoB comparisons to each network estimate.

**Supplementary Figure S35** – Depression: Risk of bias network for pairwise comparisons.

**Supplementary Figure S36** –Depression: Contribution of low, moderate, and high RoB comparisons to each network estimate.

**Supplementary Figure S37** – Quality of Life: Risk of bias network for pairwise comparisons.

**Supplementary Figure S38** – Quality of Life: Contribution of low, moderate, and high RoB comparisons to each network estimate.

**Supplementary Methods 1 –** Database search strategy.

## PubMed

| | | |
|---|---|---|
| #1 | "Autism Spectrum Disorder"[Mesh] | 48,368 |
| #2 | ((((((Autistic Spectrum Disorders[Title/Abstract]) OR (Disorder, Autistic Spectrum[Title/Abstract])) OR (Autism Spectrum Disorders[Title/Abstract])) OR (PDD[Title/Abstract])) OR (PDD[Title/Abstract])) OR (Asperge[Title/Abstract])) OR (PDD-NOS[Title/Abstract]) | 19,583 |
| **#3** | **("Autism Spectrum Disorder"[Mesh]) OR (((((((Autistic Spectrum Disorders[Title/Abstract]) OR (Disorder, Autistic Spectrum[Title/Abstract])) OR (Autism Spectrum Disorders[Title/Abstract])) OR (PDD[Title/Abstract])) OR (PDD[Title/Abstract])) OR (Asperge[Title/Abstract])) OR (PDD-NOS[Title/Abstract]))** | **59,907** |
| #4 | "Exercise"[Mesh] | 271,145 |
| #5 | "Mindfulness"[Mesh] | 8,239 |
| #6 | "Relaxation"[Mesh] | 22,638 |
| #7 | ((((((((((((((((sports[Title/Abstract]) OR (Therapeutics[Title/Abstract])) OR (therapy[Title/Abstract])) OR (yoga[Title/Abstract])) OR (music[Title/Abstract])) OR (education[Title/Abstract])) OR (Qigong[Title/Abstract])) OR (art[Title/Abstract])) OR (Dancing[Title/Abstract])) OR (sport*[Title/Abstract])) OR (Physical Activit*[Title/Abstract])) OR (training*[Title/Abstract])) OR (therap*[Title/Abstract])) OR (education*[Title/Abstract])) OR (Ch'i Kung[Title/Abstract])) OR (art*[Title/Abstract])) OR (Danc*[Title/Abstract])) OR (cognitive Behavioral[Title/Abstract]) | 5,650,070 |
| **#8** | **((("Exercise"[Mesh]) OR ("Mindfulness"[Mesh])) OR ("Relaxation"[Mesh])) OR (((((((((((((((((sports[Title/Abstract]) OR (Therapeutics[Title/Abstract])) OR (therapy[Title/Abstract])) OR (yoga[Title/Abstract])) OR (music[Title/Abstract])) OR (education[Title/Abstract])) OR (Qigong[Title/Abstract])) OR (art[Title/Abstract])) OR (Dancing[Title/Abstract])) OR (sport*[Title/Abstract])) OR (Physical Activit*[Title/Abstract])) OR (training*[Title/Abstract])) OR (therap*[Title/Abstract])) OR (education*[Title/Abstract])) OR (Ch'i Kung[Title/Abstract])) OR (art*[Title/Abstract])) OR (Danc*[Title/Abstract])) OR (cognitive Behavioral[Title/Abstract]))** | **5,797,017** |
| #9 | "Anxiety"[Mesh] | 126,426 |
| #10 | ((((Anxiet*[Title/Abstract]) OR (Angst[Title/Abstract])) OR (Nervousn | 314,415 |

| | | |
|---|---|---|
| | ess[Title/Abstract])) OR (Hypervigilance[Title/Abstract])) OR (Anxiousness[Title/Abstract]) | |
| #11 | "Depression"[Mesh] | 169,364 |
| #12 | (Symptom*, Depressive[Title/Abstract]) OR (Depressive Symptom*[Title/Abstract]) | 81,904 |
| #13 | "Quality of Life"[Mesh] | 305,049 |
| #14 | ((((Life Quality[Title/Abstract]) OR (Health-Related Quality Of Life [Title/Abstract])) OR (HRQOL[Title/Abstract])) OR (mental health[Title/Abstract])) OR (psychological health[Title/Abstract]) | 358,276 |
| #15 | **((((("Anxiety"[Mesh]) OR (((((Anxiet*[Title/Abstract]) OR (Angst[Title/Abstract])) OR (Nervousness[Title/Abstract])) OR (Hypervigilance[Title/Abstract])) OR (Anxiousness[Title/Abstract]))) OR ("Depression"[Mesh])) OR ((Symptom*, Depressive[Title/Abstract]) OR (Depressive Symptom*[Title/Abstract]))) OR ("Quality of Life"[Mesh])) OR (((HRQOL[Title/Abstract]) OR (mental health[Title/Abstract])) OR (psychological health[Title/Abstract]))** | **960,503** |
| #16 | **(((((((randomized controlled trial[Title/Abstract]) OR (randomized[Title/Abstract])) OR (placebo[Title/Abstract])) OR (randomized clinical trial*[Title/Abstract])) OR (randomized controlled clinical trial*[Title/Abstract])) OR (RCT[Title/Abstract])) OR (random control test*[Title/Abstract])) OR (randomized experiment*[Title/Abstract])** | **904,396** |
| **#17** | **#3 AND #8 AND #15 AND #16** | **347** |

**Search date: March 28, 2025.**
**Update date: May 11, 2025.**

To reduce the likelihood of missing relevant trials, we additionally reviewed the reference lists of systematic reviews published in the past two years and performed limited manual searches to identify potentially eligible studies that may not have been retrieved through database queries.

**Supplementary Methods 2 –** Conversion formula for standard deviation (SD).

**For studies that did not report standard deviations (SD) directly, we estimated SDs using the following formulas, in line with the Cochrane Handbook and published recommendations (Luo et al., 2018; Wan et al., 2014):**

**1. From Standard Error (SE):**

SD = SE × $\sqrt{N}$

SE: standard error; N: sample size.

**2. From 95% Confidence Interval (CI):**

If N ⩾ 100 (per group):

SD = (Upper limit − Lower limit) / 3.92

If N ⩽ 60 (per group):

SD = (Upper limit − Lower limit) / t<sub>inv</sub>(0.975, n − 1)

t<sub>inv</sub>: inverse t-distribution value with (n − 1) degrees of freedom.

If 60 < N < 100:

Both methods were considered appropriate, with sensitivity analyses conducted when needed.

**3. From Range:**

SD = (Max − Min) / 4

**4. From Interquartile Range (IQR):**

SD = (Q3 − Q1) / 1.35

**Note: All conversions were performed using standard statistical functions in Excel.**

**Supplementary Methods 3 –** Explanation of technical terms.

| | |
|---|---|
| **Standardized Mean Difference (SMD)** | The **Standardized Mean Difference (SMD)** is a metric used in meta-analyses to quantify the effect size between intervention and control groups across studies that may use different measurement scales. It is calculated by dividing the difference in group means by the pooled standard deviation, allowing for comparability across heterogeneous outcome measures. In this study, SMDs were used to synthesize the effects of diverse non-pharmacological interventions on anxiety, depression, and quality of life in individuals with autism spectrum disorder (ASD). |
| **95% Credible Interval (CI)** | The 95% **Confidence Interval (CI)** is a frequentist statistical concept that defines a range of values that, under repeated sampling, would contain the true parameter value 95% of the time. In our network meta-analysis, CIs were reported to reflect the uncertainty around the SMD estimates for each treatment comparison. |
| **Statistical Significance** | **Statistical significance** was determined based on whether the 95% CI excluded zero. If the entire interval lies above or below zero, the difference between the compared interventions is considered statistically significant, indicating that the observed effect is unlikely to be due to chance alone. |
| **Heterogeneity ($I^2$ Statistic)** | **Heterogeneity** refers to the variability in effect sizes across included studies. In this analysis, statistical heterogeneity was assessed using the $I^2$ statistic, which quantifies the proportion of total variation due to between-study differences rather than random error. Values of $I^2 >$ 50% were interpreted as moderate to substantial heterogeneity, warranting careful consideration in result interpretation and sensitivity analysis. |
| **Transitivity Assumption** | The **transitivity assumption** underpins the validity of indirect comparisons in network meta-analysis. It requires that the included studies are sufficiently similar in terms of population characteristics, intervention settings, and outcome measurements. For example, it assumes that participants receiving intervention A in one study |

| | |
|---|---|
| | would have been eligible to receive intervention B in another study. This assumption allows for valid comparisons across interventions even when direct head-to-head data are absent. |
| **Consistency** | **Consistency** refers to the agreement between direct and indirect evidence in a network of studies. Consistency was evaluated both globally and locally in this study. The global inconsistency was assessed using the design-by-treatment interaction model, while local inconsistency was checked via loop-specific and node-splitting approaches. Inconsistency indicates a potential violation of transitivity or methodological heterogeneity and affects the credibility of the network estimates. |
| **SUCRA** | **SUCRA** is a metric used to rank the relative effectiveness of different interventions. It ranges from 0 to 1, with higher values indicating better performance. A SUCRA value close to 1 suggests that the intervention is likely to be among the most effective, whereas a value close to 0 suggests low efficacy. In this study, SUCRA was used to compare and rank non-pharmacological interventions across the three outcomes. |

**Supplementary Methods 4 –** Inclusion in the list of studies.


1. Braden, B. B., Pagni, B. A., Monahan, L., Walsh, M. J. M., Dixon, M. V., Delaney, S., Ballard, L., & Ware, J. E., Jr (2022). Quality of life in adults with autism spectrum disorder: influence of age, sex, and a controlled, randomized mindfulness-based stress reduction pilot intervention. Quality of life research : an international journal of quality of life aspects of treatment, care and rehabilitation, 31(5), 1427–1440. https://doi.org/10.1007/s11136-021-03013-x
2. Capriola-Hall, N. N., Brewe, A. M., Golt, J., & White, S. W. (2021). Anxiety and Depression Reduction as Distal Outcomes of a College Transition Readiness Program for Adults with Autism. Journal of autism and developmental disorders, 51(1), 298–306. https://doi.org/10.1007/s10803-020-04549-6
3. Clarke, C., Hill, V., & Charman, T. (2017). School based cognitive behavioural therapy targeting anxiety in children with autistic spectrum disorder: a quasi-experimental randomised controlled trail incorporating a mixed methods approach. Journal of autism and developmental disorders, 47(12), 3883–3895. https://doi.org/10.1007/s10803-016-2801-x
4. Cook, Julia M.; Donovan, Caroline L.; Garnett, Michelle S. (2017). Parent-mediated cognitive behavioural therapy for young children with high-functioning autism spectrum disorder and anxiety: a randomized control trial. Early Child Development and Care, (1–16), doi:10.1080/03004430.2017.1303684
5. Corbett, B. A., Blain, S. D., Ioannou, S., & Balser, M. (2017). Changes in anxiety following a randomized control trial of a theatre-based intervention for youth with autism spectrum disorder. Autism : the international journal of research and practice, 21(3), 333–343. https://doi.org/10.1177/1362361316643623
6. Felicia Cruz Pryor;Alan Lincoln;Robyn Igelman;Varvara Toma;Roya Iravani; (2021). Efficacy of a computer-assisted cognitive-behavior therapy program for treating youth with anxiety and co-occurring autism spectrum disorder: Camp Cope-A-Lot . Research in Autism Spectrum Disorders. doi:10.1016/j.rasd.2021.101748
7. Frolli, A., Ricci, M. C., Di Carmine, F., Orefice, A., Saviano, E., & Carotenuto, M. (2021). Emotional Rational Education Training Associated with Mindfulness for Managing Anxiety within Adolescents Affected by High-Functioning Autism: A DesCIptive Study. Behavioral sciences (Basel, Switzerland), 11(11), 156. https://doi.org/10.3390/bs11110156
8. Gaigg, S. B., Flaxman, P. E., McLaven, G., Shah, R., Bowler, D. M., Meyer, B., Roestorf, A., Haenschel, C., Rodgers, J., & South, M. (2020). Self-guided mindfulness and cognitive behavioural practices reduce anxiety in autistic adults: A pilot 8-month waitlist-controlled trial of widely available online tools. Autism : the international journal of research and practice, 24(4), 867–883. https://doi.org/10.1177/1362361320909184
9. D. A. García-Villamisar, & Dattilo, J. . (2010). Effects of a leisure programme on quality of life and stress of individuals with asd. J Intellect Disabil Res, 54(7), 611-61
10. Guzick, A. G., Schneider, S. C., Kook, M., Greenberg, R., Perozo-Garcia, A., L

ee, M. P., Garcia, J., Onyeka, O. C., Riddle, D. B., & Storch, E. A. (2024). Internet-based, parent-led cognitive behavioral therapy for autistic youth with anxiety-related disorders: A randomized trial comparing email vs. telehealth support. Behaviour research and therapy, 183, 104639. https://doi.org/10.1016/j.brat.2024.104639

11. Huntjens, A., van den Bosch, L. W., Sizoo, B., Kerkhof, A., Smit, F., & van der Gaag, M. (2024). The effectiveness and safety of dialectical behavior therapy for suicidal ideation and behavior in autistic adults: a pragmatic randomized controlled trial. Psychological medicine, 54(10), 2707-2718.

12. Lee, D., Frey, G. C., Cothran, D. J., Harezlak, J., & Shih, P. C. (2022). Effects of a Gamified, Behavior Change Technique-Based Mobile App on Increasing Physical Activity and Reducing Anxiety in Adults With Autism Spectrum Disorder: Feasibility Randomized Controlled Trial. JMIR formative research, 6(7), e35701. https://doi.org/10.2196/35701

13. Luxford, S., Hadwin, J. A., & Kovshoff, H. (2017). Evaluating the effectiveness of a school-based cognitive behavioural therapy intervention for anxiety in adolescents diagnosed with autism spectrum disorder. Journal of Autism and Developmental Disorders, 47, 3896-3908.

14. Lindor, E. , Millard, O. , Papadopoulos, N. , Devenish, B. D. , Bellows, S. , & Mantilla, A. , et al. (2023). The feasibility and acceptability of allplay dance for autistic children: a pilot randomised controlled trial. Research in autism spectrum disorders, 109.

15. McGillivray, J. A., & Evert, H. T. (2014). Group cognitive behavioural therapy program shows potential in reducing symptoms of depression and stress among young people with ASD. Journal of autism and developmental disorders, 44(8), 2041–2051. https://doi.org/10.1007/s10803-014-2087-9

16. McKenzie, R., Dallos, R., Stedmon, J., Hancocks, H., Vickery, P. J., Barton, A., ... & Ewings, P. (2020). SAFE, a new therapeutic intervention for families of children with autism: a randomised controlled feasibility trial. BMJ open, 10(12), e038411.

17. McNally Keehn, R. H., Lincoln, A. J., Brown, M. Z., & Chavira, D. A. (2013). The Coping Cat program for children with anxiety and autism spectrum disorder: a pilot randomized controlled trial. Journal of autism and developmental disorders, 43(1), 57–67. https://doi.org/10.1007/s10803-012-1541-9

18. Mills, W., Kondakis, N., Orr, R., Warburton, M., & Milne, N. (2020). Does Hydrotherapy Impact Behaviours Related to Mental Health and Well-Being for Children with Autism Spectrum Disorder? A Randomised Crossover-Controlled Pilot Trial. International journal of environmental research and public health, 17(2), 558. https://doi.org/10.3390/ijerph17020558

19. McVey, A. J., Dolan, B. K., Willar, K. S., Pleiss, S., Karst, J. S., Casnar, C. L., Caiozzo, C., Vogt, E. M., Gordon, N. S., & Van Hecke, A. V. (2016). A Replication and Extension of the PEERS® for Young Adults Social Skills Intervention: Examining Effects on Social Skills and Social Anxiety in Young Adults with Autism Spectrum Disorder. Journal of autism and developmental disorders, 46(12), 3739–3754. https://doi.org/10.1007/s10803-016-2911-5

20. Sizoo, B. B., & Kuiper, E. (2017). Cognitive behavioural therapy and mindfulness based stress reduction may be equally effective in reducing anxiety and depression in adults with autism spectrum disorders. Research in developmental disabilities, 64, 47–55. https://doi.org/10.1016/j.ridd.2017.03.004
21. HansashreePadmanabha, PratibhaSinghi, JitendraKumarSahu, & PrahbhjotMalhi. (2019). Home-based sensory interventions in children with autism spectrum disorder: a randomized controlled trial. Indian Journal of Pediatrics, 86(1), 8.
22. Pagni, B. A., Hill, E., Walsh, M. J. M., Delaney, S., Ogbeama, D., Monahan, L., Cook, J. R., Guerithault, N., Dixon, M. V., Ballard, L., & Braden, B. B. (2023). Distinct and shared therapeutic neural mechanisms of mindfulness-based and social support stress reduction groups in adults with autism spectrum disorder. Journal of psychiatry & neuroscience : JPN, 48(2), E102–E114. https://doi.org/10.1503/jpn.220159
23. Pagni, B. A. , Walsh, M. J. M. , Foldes, E. , Sebren, A. , Dixon, M. V. , & Guerithault, N. , et al. (2020). The neural correlates of mindfulness-induced depression reduction in adults with autism spectrum disorder: a pilot study. Journal of neuroscience research, 98(6), 1150-1161.
24. Papadopoulos, N. , Sciberras, E. , Hiscock, H. , Williams, K. , Mcgillivray, J. , & Mihalopoulos, C. , et al. (2022). Sleeping sound autism spectrum disorder (asd): a randomised controlled trial of a brief behavioural sleep intervention in primary school-aged autistic children. Journal of child psychology and psychiatry, and allied disciplines, 63(11), 1423-1433.
25. Prasitwut, P., Wantanakorn, P., Chuchottaworn, K., Reangkanjanaseart, S., & Chuthapisith, J. (2024). Effectiveness of Fabric Weaving Therapy for Children with Autism Spectrum Disorder: A Randomized Waitlist-Controlled Trial. Journal of integrative and complementary medicine, 30(3), 261–268. https://doi.org/10.1089/jicm.2023.0027
26. Quadt, L., Garfinkel, S. N., Mulcahy, J. S., Larsson, D. E., Silva, M., Jones, A. M., Strauss, C., & CItchley, H. D. (2021). Interoceptive training to target anxiety in autistic adults (ADIE): A single-center, superiority randomized controlled trial. EClinicalMedicine, 39, 101042. https://doi.org/10.1016/j.eclinm.2021.101042
27. Judy, Reaven, Audrey, Blakeley-Smith, Kathy, & Culhane-Shelburne, et al. (2012). Group cognitive behavior therapy for children with high-functioning autism spectrum disorders and anxiety: a randomized trial. Journal of Child Psychology & Psychiatry.
28. Russell, A. , Gaunt, D. M. , Cooper, K. , Barton, S. , Horwood, J. , & Kessler, D. , et al. (2020). The feasibility of low-intensity psychological therapy for depression co-occurring with autism in adults: the autism depression trial (adept) - a pilot randomised controlled trial (november, 10.1177/1362361319889272, 2019). Autism :(6), 24.
29. Schiltz, Hillary K.; McVey, Alana J.; Dolan, Bridget K.; Willar, Kirsten S.; Pleiss, Sheryl; Karst, Jeffrey S.; Carson, Audrey M.; Caiozzo, Christina; Vogt, Elisabeth M.; Yund, Brianna D.; Van Hecke, Amy Vaughan (2017). Changes in Depressive Symptoms Among Adolescents with ASD Completing the PEERS® Social Skills I

ntervention. Journal of Autism and Developmental Disorders.https://doi.org/10.1007/s10803-017-3396-6
30. Spek, A. A. , Van Ham, N. C. , & Nyklí?ek, Ivan. (2013). Mindfulness-based therapy in adults with an autism spectrum disorder: a randomized controlled trial. Research in Developmental Disabilities, 34(1), 246-253.
31. Storch, E. A. , Arnold, E. B. , Lewin, A. B. , Nadeau, J. M. , Jones, A. M. , & De Nadai, A. S. , et al. (2013). The effect of cognitive-behavioral therapy versus treatment as usual for anxiety in children with autism spectrum disorders: a randomized, controlled trial. Journal of the American Academy of Child and Adolescent Psychiatry, 52(2), 132-142.e2.
32. Storch, E. A., Schneider, S. C., De Nadai, A. S., Selles, R. R., McBride, N. M., Grebe, S. C., Bergez, K. C., Ramirez, A., Viana, A. G., & Lewin, A. B. (2020). A Pilot Study of Family-Based Exposure-Focused Treatment for Youth with Autism Spectrum Disorder and Anxiety. Child psychiatry and human development, 51(2), 209–219. https://doi.org/10.1007/s10578-019-00923-3
33. Sung, M. , Ooi, Y. P. , Goh, T. J. , Pathy, P. , Fung, D. S. S. , & Ang, R. P. , et al. (2011). Effects of cognitive-behavioral therapy on anxiety in children with autism spectrum disorders: a randomized controlled trial. Child Psychiatry & Human Development, 42(6), 634-649.
34. Wijnhoven, L. A. M. W., Creemers, D. H. M., Vermulst, A. A., Lindauer, R. J. L., Otten, R., Engels, R. C. M. E., & Granic, I. (2020). Effects of the video game 'Mindlight' on anxiety of children with an autism spectrum disorder: A randomized controlled trial. Journal of behavior therapy and experimental psychiatry, 68, 101548. https://doi.org/10.1016/j.jbtep.2020.101548
35. Wood, J. J., Drahota, A., Sze, K., Har, K., Chiu, A., & Langer, D. A. (2009). Cognitive behavioral therapy for anxiety in children with autism spectrum disorders: A randomized, controlled trial. Journal of child psychology and psychiatry, 50(3), 224-234.
36. Wood, J. J., Ehrenreich-May, J., Alessandri, M., Fujii, C., Renno, P., Laugeson, E., ... & Storch, E. A. (2015). Cognitive behavioral therapy for early adolescents with autism spectrum disorders and clinical anxiety: A randomized, controlled trial. Behavior therapy, 46(1), 7-19.
37. Westerberg, B., Holländare, F., & Bejerot, S. (2023). An internet-based behavioral intervention for adults with autism spectrum disorder - A randomized controlled trial and feasibility study. Internet interventions, 34, 100672. https://doi.org/10.1016/j.invent.2023.100672
38. Wood, J. J., Kendall, P. C., Wood, K. S., Kerns, C. M., Seltzer, M., Small, B. J., ... & Storch, E. A. (2020). Cognitive behavioral treatments for anxiety in children with autism spectrum disorder: A randomized clinical trial. JAMA psychiatry, 77(5), 474-483.
39. Yoo, H. J., Bahn, G., Cho, I. H., Kim, E. K., Kim, J. H., Min, J. W., Lee, W. H., Seo, J. S., Jun, S. S., Bong, G., Cho, S., Shin, M. S., Kim, B. N., Kim, J. W., Park, S., & Laugeson, E. A. (2014). A randomized controlled trial of the Korean version of the PEERS(®) parent-assisted social skills training program for teens

with ASD. Autism research : official journal of the International Society for Autism Research, 7(1), 145–161. https://doi.org/10.1002/aur.1354
40. Yang, Y. J., & Chung, K. M. (2023). Pilot randomized control trial of an app-based CBT program for reducing anxiety in individuals with ASD without intellectual disability. Journal of Autism and Developmental Disorders, 53(4), 1331-1346.
41. Adams, D., Malone, S., Dargue, N., Keen, D., Rodgers, J., Simpson, K., Wicks, R., Bullot, A., & Rapee, R. (2024). Prevention and Reduction of Anxiety in Autistic Preschoolers Through an Autism-Specific Parent-Mediated Intervention: A Pilot Randomised Controlled Trial Evaluating Short and Longer Term Outcomes. Journal of autism and developmental disorders, 10.1007/s10803-024-06570-5. Advance online publication. https://doi.org/10.1007/s10803-024-06570-5
42. Akhani, A., Dehghani, M., Gharraee, B., & Hakim Shooshtari, M. (2021). Parent training intervention for autism symptoms, functional emotional development, and parental stress in children with autism disorder: A randomized clinical trial. Asian journal of psychiatry, 62, 102735. https://doi.org/10.1016/j.ajp.2021.102735
43. Aljubour, A., AbdElBaki, M., El Meligy, O., Al-Jabri, B., & Sabbagh, H. (2023). Effect of Culturally Adapted Dental Visual Aids on Anxiety Levels in Children with Autism Spectrum Disorder: A Randomized Clinical Trial. Children (Basel, Switzerland), 10(6), 1040. https://doi.org/10.3390/children10061040
44. Chalfant, A. M., Rapee, R., & Carroll, L. (2007). Treating anxiety disorders in children with high functioning autism spectrum disorders: a controlled trial. Journal of autism and developmental disorders, 37(10), 1842–1857. https://doi.org/10.1007/s10803-006-0318-4
45. Chien, Y. L., Tsai, W. C., Chen, W. H., Yang, C. L., Gau, S. S., Soong, W. T., Laugeson, E., & Chiu, Y. N. (2023). Effectiveness, durability, and clinical correlates of the PEERS social skills intervention in young adults with autism spectrum disorder: the first evidence outside North America. Psychological medicine, 53(3), 966–976. https://doi.org/10.1017/S0033291721002385
46. Conaughton, R. J., Donovan, C. L., & March, S. (2017). Efficacy of an internet-based CBT program for children with comorbid High Functioning Autism Spectrum Disorder and anxiety: A randomised controlled trial. Journal of affective disorders, 218, 260–268. https://doi.org/10.1016/j.jad.2017.04.032
47. Fatta, L. M., Laugeson, E. A., Bianchi, D., Italian Peers® team support group, Laghi, F., & Scattoni, M. L. (2025). Program for the Education and Enrichment of Relational Skills (PEERS®) for Italy: A Randomized Controlled Trial of a Social Skills Intervention for Autistic Adolescents. Journal of autism and developmental disorders, 55(1), 202–220. https://doi.org/10.1007/s10803-023-06211-3
48. Hawkins, J. R., Weatherby, N., Wrye, B., & Ujcich Ward, K. (2019). Bergamot Aromatherapy for Medical Office-Induced Anxiety Among Children With an Autism Spectrum Disorder: A Randomized, Controlled, Blinded Clinical Trial. Holistic nursing practice, 33(5), 285–294. https://doi.org/10.1097/HNP.0000000000000341
49. Gao, X. , & Drani, S. . (2024). Parent-implemented interventions in chinese families of children with autism spectrum disorder. PALGRAVE COMMUNICATIONS, 10(1).

50. Hesselmark, E., Plenty, S., & Bejerot, S. (2014). Group cognitive behavioural th erapy and group recreational activity for adults with autism spectrum disorders: a p reliminary randomized controlled trial. Autism : the international journal of research and practice, 18(6), 672–683. https://doi.org/10.1177/1362361313493681
51. Howells, K., Sivaratnam, C., Lindor, E., Hyde, C., McGillivray, J., Whitehouse, A., & Rinehart, N. (2020). Can Participation in a Community Organized Football P rogram Improve Social, Behavioural Functioning and Communication in Children wi th Autism Spectrum Disorder? A Pilot Study. Journal of autism and developmental disorders, 50(10), 3714–3727. https://doi.org/10.1007/s10803-020-04423-5
52. Ireri, N. W., White, S. W., & Mbwayo, A. W. (2019). Treating anxiety and so cial deficits in children with autism spectrum disorder in two schools in Nairobi, K enya. Journal of autism and developmental disorders, 49, 3309-3315.
53. Kilburn, T. R. , Srensen, M. J. , Thastum, M. , Rapee, R. M. , & Thomse n, P. H. . (2020). Group based cognitive behavioural therapy for anxiety in childre n with autism spectrum disorder: a randomised controlled trial in a general child ps ychiatric hospital setting. Journal of Autism and Developmental Disorders(8).
54. McConachie, H., McLaughlin, E., Grahame, V., Taylor, H., Honey, E., Tavernor, L., ... & Le Couteur, A. (2014). Group therapy for anxiety in children with autis m spectrum disorder. Autism, 18(6), 723-732.
55. Nadig, A., Flanagan, T., White, K., & Bhatnagar, S. (2018). Results of a RCT on a Transition Support Program for Adults with ASD: Effects on Self-Determinati on and Quality of Life. Autism research : official journal of the International Socie ty for Autism Research, 11(12), 1712–1728. https://doi.org/10.1002/aur.2027
56. Santomauro, D., Sheffield, J., & Sofronoff, K. (2016). Depression in Adolescent s with ASD: A Pilot RCT of a Group Intervention. Journal of autism and develop mental disorders, 46(2), 572–588. https://doi.org/10.1007/s10803-015-2605-4
57. Wijker, C., Leontjevas, R., Spek, A., & Enders-Slegers, M. J. (2020). Effects of Dog Assisted Therapy for Adults with Autism Spectrum Disorder: An Exploratory Randomized Controlled Trial. Journal of autism and developmental disorders, 50(6), 2153–2163. https://doi.org/10.1007/s10803-019-03971-9
58. Gerber, A. H., Nahmias, A., Schleider, J. L., & Lerner, M. D. (2024). Results f rom a Pilot Randomized Controlled Trial of a Single-Session Growth-Mindset Interv ention for Internalizing Symptoms in Autistic Youth. Journal of autism and develop mental disorders, 10.1007/s10803-024-06341-2. Advance online publication. https://do i.org/10.1007/s10803-024-06341-2
59. Backman, A. , Roll-Pettersson, L. , Bsc, A. M. , Bsc, N. C. , Sundqvist, E. , & Zander, E. , et al. (2024). Internet-delivered psychoeducation (scope) for tr ansition-aged autistic youth: pragmatic randomized controlled trial. JOURNAL OF MEDICAL INTERNET RESEARCH, 26(000).
60. Baghdadli, A., Brisot, J., Henry, V., Michelon, C., Soussana, M., Rattaz, C., & Picot, M. C. (2013). Social skills improvement in children with high-functioning aut ism: a pilot randomized controlled trial. European child & adolescent psychiatry, 22 (7), 433–442. https://doi.org/10.1007/s00787-013-0388-8
61. Chien, Y. L., Tsai, W. C., Chen, W. H., Yang, C. L., Gau, S. S., Soong, W.

T., Laugeson, E., & Chiu, Y. N. (2023). Effectiveness, durability, and clinical correlates of the PEERS social skills intervention in young adults with autism spectrum disorder: the first evidence outside North America. Psychological medicine, 53(3), 966–976. https://doi.org/10.1017/S0033291721002385

62. Corbett, B. A., White, S., Lerner, M., Preacher, K. J., Klemencic, M. E., Simmons, G. L., Pilkington, J., Gable, P., Gioia, A., & Key, A. P. (2023). Peers, play, and performance to build social salience in autistic youth: A multisite randomized clinical trial. Journal of consulting and clinical psychology, 91(7), 411–425. https://doi.org/10.1037/ccp0000821

63. Ozyurt, GoncaDinsever, CaglaAkpinar, SelcukOzcan, KursatSal, YucelOzturk, Yusuf. (2017). The effect of therapeutic horseback riding for children diagnosed with autism spectrum disorder on autistic symptoms and the quality of life. Journal of Turbulence, 18(6).

64. Pahnke, J., Jansson-Fröjmark, M., Andersson, G., Bjureberg, J., Jokinen, J., Bohman, B., & Lundgren, T. (2023). Acceptance and commitment therapy for autistic adults: A randomized controlled pilot study in a psychiatric outpatient setting. Autism : the international journal of research and practice, 27(5), 1461–1476. https://doi.org/10.1177/13623613221140749

65. Storch, E. A., Lewin, A. B., Collier, A. B., Arnold, E., De Nadai, A. S., Dane, B. F., Nadeau, J. M., Mutch, P. J., & Murphy, T. K. (2015). A randomized controlled trial of cognitive-behavioral therapy versus treatment as usual for adolescents with autism spectrum disorders and comorbid anxiety. Depression and anxiety, 32(3), 174–181. https://doi.org/10.1002/da.22332

66. White, S. W., Ollendick, T., Albano, A. M., Oswald, D., Johnson, C., Southam-Gerow, M. A., Kim, I., & Scahill, L. (2013). Randomized controlled trial: Multimodal Anxiety and Social Skill Intervention for adolescents with autism spectrum disorder. Journal of autism and developmental disorders, 43(2), 382–394. https://doi.org/10.1007/s10803-012-1577-x

67. Matthews, N. L., Mitchell, M. M., Honda, H., Malligo, A., Boyd, S., Pagni, B. A., & Blair Braden, B. (2025). Pilot randomized controlled trial of MINDful TIME, a novel telehealth mindfulness-based intervention for autistic adolescents and their caregivers. Autism : the international journal of research and practice, 13623613251328484. Advance online publication. https://doi.org/10.1177/13623613251328484

**Supplementary Table S1 –** Definition of Interventions and Controls.

| **Intervention** | **Abbreviation** | **Definition** |
|---|---|---|
| **Control** | | |
| 1 Control group (Usual care) | CTRL | The control group refers to participants who did not receive targeted therapeutic intervention. This includes usual care, waitlist control, psychoeducation, or general health consultations. |
| **Experimental intervention** | | |
| 2 Mindfulness-Based Interventions | MBI | Mindfulness interventions are psychological approaches emphasizing present-moment awareness and non-judgmental acceptance. Common forms include Mindfulness-Based Stress Reduction (MBSR) and Acceptance and Commitment Therapy (ACT), often used to regulate mood and alleviate anxiety and depression. |
| 3 Cognitive Behavioral Therapy | CBT | CBT encompasses classical cognitive behavioral therapy and related variants (e.g., dialectical behavior therapy), aiming to restructure maladaptive thoughts and behaviors through structured, goal-oriented sessions. |
| 4 Behavioral and Functional Training | BEHAVE | This category includes structured interventions such as PEERS, STEPS, transition support programs, and social skills training. These aim to enhance adaptive functioning and social interaction skills. |
| 5 Physical Activity | PHYS | Interventions involving physical activity such as AllPlay dance, Auskick football, LEGO therapy, therapeutic horseback riding, or leisure sports. These aim to promote physical health, emotional regulation, and engagement. |
| 6 Sensory-Based Therapy | SENS | Includes interventions using environmental or sensory stimuli for regulation, such as hydrotherapy, aromatherapy, visual support, and sensory integration therapy. |
| 7 Technology- and Family-Based Interventions | TAFI | Includes interventions delivered through digital media or family participation, such as VR (virtual reality), Mindlight games, parent-implemented training, and home-based support programs. |
| 8 Other Therapies | OTH | This group includes therapies not categorized above, such as animal-assisted therapy, theater-based programs, growth mindset training, sleep interventions, and personality development projects. |

**Supplementary Table S2 –** Anxiety,Depression and Quality of Life rating scale.

## Anxiety

| Abbreviation | Range | Full title |
|---|---|---|
| ASR-AP | 0-100 | Adult Self-Report – Anxiety Problems Subscale |
| SCAS-P | 0-114 | Spence Children's Anxiety Scale-parent version |
| CBCL-Anx | 0-100 | Child Behavior Checklist – Anxiety Problems Subscale |
| STAI-C | 20-60 | State–Trait-anxiety Inventory for Children |
| ADIS-P PIR | 0-8 | Anxiety Disorders Interview Schedule – Parent Version, Clinician Severity Rating |
| SCARED | 0-82 | Screen for Child Anxiety Related Emotional Disorders |
| GAD-7 | 0-21 | Generalized Anxiety Disorder – 7 Item Scale |
| PARS-A | 0-25 | Pediatric Anxiety Rating Scale – Modified for Autism |
| SIAS | 0-60 | Social Interaction Anxiety Scale |
| BAI | 0-63 | Beck Anxiety Inventory |
| SCAS | 0-114 | Spence Children's Anxiety Scale – Parent and Self Versions |
| DASS-Anx | 0-42 | Depression Anxiety Stress Scales – Anxiety Subscale |
| ADIS-P | 0-8 | Anxiety Disorders Interview Schedule – Parent Version |
| LSAS-SR | 0-144 | Liebowitz Social Anxiety Scale – Self-Report |
| HADS-A | 0-21 | Hospital Anxiety and Depression Scale – Anxiety Subscale |
| STAI-2 | 20–80 | State–Trait Anxiety Inventory – Trait Version |
| DBC–A | 0-3 | Developmental Behaviour Checklist (DBC) – Anxiety Subscale |
| STAI-T | 20-80 | Spielberger State–Trait Anxiety Inventory – Trait Version |
| SCL-90-R(Anxiety) | 16-80 | Symptom Checklist-90-Revised – Anxiety Subscale |
| PARS | 0-25 | Pediatric Anxiety Rating Scale |
| SCAS-C | 0-114 | Spence Children's Anxiety Scale – Child version |
| MASC-P | 0-117 | Multidimensional Anxiety Scale for Children – Parent Version |
| ASC-ASD-P | -- | Anxiety Scale for Children – Autism Spectrum Disorder Parent report |
| MASC-2 | 0-150 | Multidimensional Anxiety Scale for Children – 2nd Edition |
| STAI-CH | 20-60 | State-Trait Anxiety Inventory for Children |
| CASI-Anx | 0-78 | Child and Adolescent Symptom Inventory – ASD Anxiety Scale |
| STAI-S | 20-80 | State-Trait Anxiety Inventory – State subscale |

## Depression

| Abbreviation | Range | Full title |
|---|---|---|
| ASR-DP | 0-100 | Adult Self-Report – Depressive Problems Subscale |
| CDI-2 | 0-54 | Children's Depression Inventory – Second Edition |
| BDI-II | 0-63 | Beck Depression Inventory – Second Edition |
| DASS-Dep | 0-42 | Depression Anxiety Stress Scales – Depression Subscale |
| HADS-D | 0-21 | Hospital Anxiety and Depression Scale – Depression Subscale |
| CDI | 35-68 | Children's Depression Inventory |
| SCL-90-R | 10-50 | Symptom Checklist-90-Revised – Depression Subscale |
| CBCL-Dep | 50-100 | Child Behavior Checklist – Depressive Problems Subscale |

## Quality of Life

| Abbreviation | Range | Full title |
|---|---|---|
| WHOQOL-BREF | 24-120 | World Health Organization Quality of Life – Brief Version |
| QOL-SV | 40-120 | Quality of Life Questionnaire – Spanish Version |
| PedsQL | 0-100 | Pediatric Quality of Life Inventory |
| CHU-9D | 0-1 | Child Health Utility 9 Dimensions |
| PedsQL | 0-100 | Pediatric Quality of Life Inventory 4.0 |
| PedsQL | 0-92 | Pediatric Quality of Life Inventory |
| EQ-5D-5L | -0.594 to 1 | EuroQol Five-Dimension Five-Level Scale |
| BBQ | 0-96 | Brunnsviken Brief Quality of Life Scale |
| FQOL | 25-125 | Beach Center Family Quality of Life Scale |
| QOLI | -6 to 6 | Quality of Life Inventory |
| QoL-Q | 10-30 | Abridged Quality of Life Questionnaire |
| Kidscreen-27-P | 27-135 | Kidscreen-27 Health-Related Quality of Life Questionnaire |

Note: Higher scores on anxiety and depression scales indicate greater symptom severity, whereas higher scores on quality of life (QoL) measures generally reflect better perceived health or overall well-being.

**Supplementary Table S3 –** Study characteristics of the included studies.

| ID | study | County | Design | Characteristics of subject | | | Interventions information | | | outcome | |
|---|---|---|---|---|---|---|---|---|---|---|---|
| | | | | Number (Female/Men) | age (mean[SD]) | Severity Level | Intervention Type | Intervention Dose (min/week) | Duration (weeks) | Reported | Measurement Tool(s) |
| 1 | Braden 2022 | USA | RCT | 12F/16M | 30.32 (11.74) | Moderate ASD | Mindfulness-Based Stress Reduction (MBSR) | 120 min/week +~45 min/day homepractice | 8 | QoL | WHOQOL-BREF |
| 2 | Hall 2020 | USA | RCT | 5F/11M | 19.87 (1.92) | High-functioning ASD | College transition readiness program (STEPS) with CBT elements | 60 min/week × 12–16 sessions + 4–6 community outings + weekly check-ins | 16 | ANX, DEP | ASR-AP; ASR-DP |
| 3 | Clarke 2016 | UK | Quasi-RCT | 14Boys | 12.64 (0.85) | Moderate ASD | School-based Cognitive Behavioural Therapy | 60 min/week × 6 sessions | 6 | ANX | SCAS-P |
| 4 | Cook 2017 | Australia | RCT | 2Girls/12Boys | 5.5 (0.88) | High-functioning ASD | Parent-mediated cognitive behavioural therapy | 90 min/week × 9 sessions + 1 booster session (one month later) | 10 | ANX | CBCL-Anx |
| 5 | Corbett 2016 | USA | RCT | 4Girls / 13Boys | 11.27 (2.51) | High-functioning ASD | Peer-mediated, theatre-based intervention (SENSE Theatre) | 240 min/week × 10 sessions | 10 | ANX | STAI-C |
| 6 | Pryor 2021 | USA | RCT (cross-over design) | 12 Boys | 11.67(2.19) | High-functioning ASD | Computer-assisted CBT(Camp Cope-A-Lot, CCAL) | 30–45 min/session × 12 sessions = ~360–540 min total; therapist-facilitated | 12 | ANX | ADIS-P PIR |
| 7 | Frolli 2021 | Italy | RCT | 7Girls / 20Boys | 13.30 (0.55) | High-functioning ASD | Mindfulness + Emotional Rational Education (M-ERE) | 60 min/week × 24 sessions (6 months) | 24 | ANX, DEP | SCARED; CDI-2 |

| | | | | | | | | | | | |
|---|---|---|---|---|---|---|---|---|---|---|---|
| 8 | Gaigg 2020 | UK | RCT | 1F/8M; 2F/12M | 40.3（12.7）; 42.5 (10.3) | High-functioning ASD | Self-guided online CBT (Serenity) or MBT (Be Mindful) | Self-paced; target completion within 6–8 weeks; average ~60–90 min/week (unstructured) | 8 | ANX | GAD-7 |
| 9 | Villamisar 2010 | Spain | RCT | 15 W / 22 M | 31.49 (4.83) | High-functioning ASD | Group-based leisure programme (media, exercise, games, crafts, events, and recreational outings) | 120 min/day × 5 days/week | 52 | QoL | QOL-SV |
| 10 | Guzick 2024 | USA | RCT | 10 Girls /18 Boys | 11.11 (2.63) | High-functioning ASD | Internet-based, parent-led CBT with telehealth support (iCBT-Video) | 30 min/week (avg) × 12 | 12 | ANX | PARS-A |
| 11 | Huntjens 2024 | Netherlands | RCT | 40 W / 33 M / 1 Non-binary | 36.9（10.6) | High-functioning ASD | Comprehensive Dialectical Behavior Therapy (DBT) | 45 min/week individual therapy + 135 min/week group training | 26 | ANX, DEP | SIAS, BDI-II |
| 12 | Lee 2022 | USA | RCT (Feasibility Trial) | 9W / 3 M | 27.1（7.5) | High-functioning ASD | Gamified behavior change mobile app (PuzzleWalk) | ~162.9 min/day → 1140 min/week | 5 | ANX | BAI |
| 13 | Luxford 2017 | UK | RCT | 4 Girls /14 Boys | 13.20 (1.10) | High-functioning ASD | School-based group CBT | 90 min/week × 6 sessions | 6 | ANX | SCAS |
| 14 | Lindor 2023 | Australia | RCT | 7 Girls / 7 Boys | 9.00（1.47) | Moderate | Community-based classical/contemporary dance program (AllPlay Dance) | 60 min/week × 10 sessions | 10 | QoL | PedsQL |
| 15 | McGillivray 2014 | Australia | Quasi-RCT | 7W / 19 M | 20.27 (4.39) | High-functioning ASD | Group-based CBT (“Think well, feel well, be well” program) | 120 min/week × 9 sessions | 9 | ANX, DEP | DASS-Anx; DASS-Dep |
| 16 | McKenzie 2020 | UK | RCT | 5 Girls /17Boys | 9.5（2.7) | Level 1–2 | Systemic Autism-related Family Enabling (SAFE) – family-based systemic | 180 min/session × 5 sessions over 16 weeks → ~56 min/week | 16 | QoL | CHU-9D |

| | | | | | | | | | | | |
|---|---|---|---|---|---|---|---|---|---|---|---|
| | | | | | | | therapy | | | | |
| 17 | Keehn 2013 | USA | RCT (pilot) | 12 Boys | 11.26 (1.53) | High-functioning ASD | Modified Coping Cat CBT program (16 weekly sessions + parent components) | 60–90 min/session × 1/week | 16 | ANX | ADIS-P |
| 18 | Mills 2020 | Australia | RCT (Cross over-controlled pilot) | 2 Girls / 6 Boys | 8.72 (1.99) | Moderate | Hydrotherapy (weekly 45-minute aquatic play sessions) | 45 min/week × 4 sessions | 4 | ANX | CBCL-Anx |
| 19 | McVey 2016 | USA | RCT | 6 W / 18 M | 20.92 (3.31) | High-functioning ASD | PEERS® for Young Adults – group-based social skills training | 90 min/week × 16 | 16 | ANX | LSAS-SR |
| 20 | Sizoo 2017 | Netherlands | RCT | 8 W / 19 M | 35.1 (9.22) | High-functioning ASD | Cognitive Behavioural Therapy (CBT), tailored for ASD | 90 min/week × 13 sessions | 13 | ANX, DEP | HADS-A; HADS-D |
| 21 | Padmanabha 2018 | India | RCT (pilot, parallel-group) | 5 Girls / 16 Boys | Median 48 months (IQR 39–60) | Severe | Home-based Sensory Interventions (HBSI) | 45–60 min/session, 5 sessions/week × 12 weeks | 12 | QoL | PedsQL |
| 22 | Pagni 2023 | USA | RCT | 26F / 13M | 31.15 (13.57) | High-functioning ASD | Mindfulness-Based Stress Reduction (MBSR) | ~120 min/week × 8 sessions | 8 | ANX, DEP | STAI-2; BDI-II |
| 23 | Pagni 2020 | USA | RCT | 8F / 7M | 32.27 (13.16) | High-functioning ASD | Mindfulness-Based Stress Reduction (MBSR) | 120 min/week × 8 sessions + ~45 min/day home practice | 8 | ANX, DEP | STAI-2; BDI-II |

| | | | | | | | | | | | |
|---|---|---|---|---|---|---|---|---|---|---|---|
| 24 | Prasitwut 2024 | Thailand | RCT | 2 girls / 9 boys | 12.2 (2.4) | Mixed (IQ mean = 65.0; 15 children with ID, 8 without ID) | Fabric Weaving Therapy | 45–60 min/week × 10 sessions (hospital-based) + ~80 min/week (median; IQR: 47.5–162.4) home-based | 24 (12 weeks hospital-based + 12 weeks home-based) | QoL | PedsQL |
| 25 | Papadopoulos 2022 | Australia | RCT | 43 girls / 80 boys | 8.45 (2.10) | ASD with SCQ score ⩾11 | Brief Behavioral Sleep Intervention ("Sleeping Sound") | Two 50-min face-to-face sessions + one 30-min follow-up phone call over 4 weeks | 4 | ANX | DBC–A |
| 26 | Quadt 2021 | UK | RCT | 29F / 32M | Median 29 years (IQR 23–43; range 18–64) | High-functioning ASD | Interoceptive Training (ADIE: Aligning Dimensions of Interoceptive Experience) | 6 sessions total over 2 months; each session ~60–75 minutes | 8 | ANX | STAI-T |
| 27 | Reaven 2012 | USA | RCT | 24 boys | 10.48 (1.79) | High-functioning ASD | Group Cognitive Behavioral Therapy (Facing Your Fears program) | 90 min/week × 12 sessions | 12 | ANX | ADIS-P |
| 28 | Russell 2020 | UK | RCT | 11F / 24M | 35.3 (13.6) | High-functioning ASD | Guided Self-Help based on Low-Intensity Cognitive Behavioural Therapy (CBT with Behavioral Activation) | 9 sessions over 10–16 weeks; each session ~30–45 min (first up to 90 min) | 16 | ANX, DEP, QoL | GAD-7; BDI-II; EQ-5D-5L |
| 29 | Schiltz 2018 | USA | RCT | 4 girls / 45 boys | 13.25 (1.07) | High-functioning ASD | PEERS® Social Skills Intervention | 90 min/week × 14 sessions | 14 | DEP | CDI |
| 30 | Spek 2013 | Netherlands | RCT | 7F / 13M | 44.4 (11.1) | High-functioning ASD | Mindfulness-Based Therapy for Autism Spectrum Disorders (MBT-AS) | 150 min/week × 9 sessions + 40–60 min/day home practice (6 days/week) | 9 | ANX, DEP | SCL-90-R |

| | | | | | | | | | | | |
|---|---|---|---|---|---|---|---|---|---|---|---|
| 31 | Storch 2013 | USA | RCT | 5 girls / 19 boys | 8.89 (1.34) | High-functioning ASD | Modular Cognitive Behavioral Therapy (BIACA program) | 60–90 min/week × 16 sessions | 16 | ANX | PARS |
| 32 | Storch 2020 | USA | RCT | 4 girls / 10 boys | 10.07 (2.89) | High-functioning ASD | Family-Based Exposure-Focused Treatment (FET) | 45–55 min/week × 12 sessions | 12 | ANX | PARS |
| 33 | Sung 2011 | Singapore | RCT | 2 girls / 34 boys | 11.33 (2.03) | High-functioning ASD | Manualized Group Cognitive Behavioral Therapy | 90 min/week × 16 sessions | 16 | ANX | SCAS-C |
| 34 | Wijnhoven 2020 | Netherlands | RCT | 15 girls / 38 boys | 11.10 (2.07) | High-functioning ASD | Mindlight video game intervention (CBT-based + neurofeedback) | 60 min/week × 6 sessions | 6 | ANX | SCAS-P |
| 35 | Wood 2009 | USA | RCT | 5 girls / 12 boys | 9.18 (1.42) | High-functioning ASD | Modular Individualized Cognitive Behavioral Therapy (CBT with parent and school involvement) | 90 min/week × 16 sessions (30 min child + 60 min parent/family) | 16 | ANX | MASC-P |
| 36 | Wood 2015 | USA | RCT | 6 girls / 13 boys | 12.4 (1.3) | High-functioning ASD | Behavioral Interventions for Anxiety in Children with Autism (BIACA, adapted for early adolescents) | 90 min/week × 16 sessions (30 min with child, 30 min with parents, 30 min joint) | 16 | ANX | PARS |
| 37 | Westerberg 2023 | Sweden | RCT | 20F / 19M | 32.76 (9.52) | High-functioning ASD | Internet-Based Behavioral Intervention (MILAS, based on CBT principles) | ~90–120 min/week × 18 modules + biweekly 60-min therapist-moderated group chats | 18 | ANX, DEP, QoL | HADS-A; HADS-D; BBQ |
| 38 | Wood 2019 | USA | RCT | 21 girls / 54 boys | 9.9 (1.8) | High-functioning ASD | Behavioral Interventions for Anxiety in Children with Autism (BIACA, adapted CBT) | 90 min/week × 16 sessions (child + parent joint sessions) | 16 | ANX; DEP | PARS |

| | | | | | | | | | | | |
|---|---|---|---|---|---|---|---|---|---|---|---|
| 39 | Yoo 2014 | Korea | RCT | 1 girl / 22 boys | 14.04 (1.64) | High-functioning ASD | Korean Version of PEERS® Parent-Assisted Social Skills Training | 90 min/week × 14 sessions | 14 | DEP | CDI |
| 40 | Adams 2024 | Australia | RCT | 11 girls / 18 boys | 4.75 (0.45) | Not specified | Parent-mediated autism-specific intervention combining CLK (Cool Little Kids) and CUES (Coping with Uncertainty in Everyday Situations) (CLK-CUES) | 120 min/week × 6 sessions | 6 | ANX | ASC-ASD-P |
| 41 | Akhani 2021 | Iran | RCT | 2 girls / 17 boys | 3.94 (0.64) | Not specified | Parent training based on Ingersoll and Dvortcsak protocol (Project ImPACT model) | Alternating weekly 120-min group sessions and 60-min 1:1 coaching sessions × 12 weeks | 12 | QoL | WHOQOL-BREF |
| 42 | Aljubour 2023 | Saudi Arabia | RCT; double-blind | 9 girls / 23 boys | 8.17 (not reported) | Mild to moderate ASD | Culturally adapted dental visual aids | 15 min/day × 7 days/week × 4 weeks = 105 min/week (parent-led exposure at home) | 4 | ANX | ASC-ASD-P |
| 43 | Chalfant 2006 | Australia | RCT | 12 girls / 16 boys | 10.77 (1.35) | High-functioning ASD | Manualized cognitive behavioural therapy (CBT) adapted from "Cool Kids" program for HFA children | 120 min/week × 12 sessions (9 weekly + 3 monthly booster sessions) | 24 | ANX | SCAS-P |
| 44 | Chien 2021 | Taiwan | RCT | 6 F / 35 M | 25.3 (4.5) | High-functioning ASD | PEERS-YA (Program for the Education and Enrichment of Relational Skills – Young Adult version), caregiver-assisted manualized social skills training | 90 min/week × 16 sessions | 16 | ANX | SIAS |

| | | | | | | | | | | | |
|---|---|---|---|---|---|---|---|---|---|---|---|
| 45 | Conaughton 2017 | Australia | RCT | 5 girls / 16 boys | 9.81 (1.30) | High-functioning ASD | BRAVE-ONLINE (unmodified internet-based CBT program for anxiety, therapist-assisted) | 60 min/week × 10 child sessions + 60 min/week × 6 parent sessions + 2 booster sessions (1 and 3 months later) | 14 | ANX | SCAS-P |
| 46 | Fatta 2024 | Italy | RCT | 6 girls / 12 boys | 15.14 (2.26) | Level 1 | Italian culturally adapted PEERS® (Program for the Education and Enrichment of Relational Skills) | 120 min/week × 14 sessions | 14 | ANX; DEP | MASC-2; BDI-II |
| 47 | Hawkins 2019 | USA | quasi-RCT | 3 girls / 8 boys | 8.54 (1.90) | Level 2 | Inhalation aromatherapy using undiluted bergamot (Citrus bergamia) essential oil via scent strip in medical office | Single dose of 15 min exposure (one-time administration during office visit) | 1 (single-session intervention) | ANX | STAI-CH |
| 48 | Gao 2024 | China | RCT | 9 girls / 26 boys | 5.49 (2.74) | Mild to severe ASD | Parent-implemented intervention (PII) based on WHO Caregiver Skills Training (CST), applied at home following structured professional training | 90–120 min/day × 7 days/week | 20 | QoL | FQOL |
| 49 | Hesselmark 2014 | Sweden | RCT | 13F / 21M | 31.9 (8.5) | severity level not specified | Group cognitive behavioural therapy (CBT) | 180 min/week × 36 sessions | 36 | DEP; QoL | BDI; QOLI |
| 50 | Howells 2020 | Australia | quasi-RCT | 2 girls / 17 boys | 7.98 (1.71) | ASD diagnosis confirmed per DSM-5/DSM-IV | Community-based organized football program (NAB AFL Auskick); naturalistic physical activity intervention | 60–90 min/week × average 12 sessions | Average 12 (Range 4–21 weeks) | ANX; DEP | CBCL-Anx; CBCL-Dep |

| | | | | | | | | | | | |
|---|---|---|---|---|---|---|---|---|---|---|---|
| 51 | Ireri 2019 | Kenya | quasi-RCT | 6 girls / 14 boys | Not reported | ASD diagnosis per DSM-5; severity not specified | MASSI (Multimodal Anxiety and Social Skills Intervention); school-based CBT program including 26 individual + 14 group sessions | 60 min/week × 40 sessions | ~24–26 weeks(2 interventi on phases + 3-week break) | ANX | CASI-Anx |
| 52 | Kilbur n 2020 | Denmar k | RCT | 10 girls / 15 boys | 11.99 (1.70) | ASD diagnosis confirmed via ICD-10 | Group CBT (Cool Kids ASD, 2nd Edition) | 120 min/week × 10 sessions | 10 | ANX | SCAS-P |
| 53 | McCon achie 2014 | UK | RCT | 2 girls / 15 boys | 11.7 (1.4) | High-function ing ASD | Group CBT using the "Exploring Feelings" manual | 120 min/week × 7 sessions | 7 | ANX | SCAS-P |
| 54 | Nadig 2018 | Canada | RCT | 7 F / 10 M | 20.65 (3.28) | High-function ing ASD | Group-based Transition Support Program tailored to self-expressed needs in social communication, self-determination, and collaboration | 120 min/week × 10 sessions | 10 | QoL | QoL-Q |
| 55 | Santo mauro 2015 | Australi a | RCT | 5 girls / 5 boys | 15.75 (1.37) | High-function ing ASD | Group CBT ("Exploring Depression" program by Attwood & Garnett) | 60 min/week × 10 sessions + 1 booster | 11 | DEP | DASS-Dep |
| 56 | Wijker 2019 | Netherl ands | RCT | 13 F / 14 M | Not reported | High-function ing ASD | Individual Animal-Assisted Therapy (AAT) with certified therapy dogs | 60 min/week × 10 sessions | 10 | DEP | SCL-90-R |
| 57 | Gerber 2023 | USA | RCT | 4 girls / 16 boys | 14.27 (1.76) | High-function ing ASD | Digital Growth Mindset Single-Session Intervention (GM-SSI; "Project Personality") | Single 30-minute session (self-guided) | 1 | ANX; DEP | MASC-2; CDI-2 |

| | | | | | | | | | | | |
|---|---|---|---|---|---|---|---|---|---|---|---|
| 58 | Backman 2024 | Sweden | RCT | 54 F / 21 M | 20.48 (3.07) | High-functioning ASD | Internet-delivered therapist-supported psychoeducational program (SCOPE) for transition-aged autistic youth; 8 weekly modules with video, text, and therapist messaging | ~60–90 min/week × 8 modules | 8 | ANX; DEP; QoL | HADS-A; HADS-D; BBQ |
| 59 | Baghdadli 2013 | France | RCT | 7 boys | 10.7 (1.8) | High-functioning ASD | Group-based Social Skills Training Program (SST-GP) with video modeling, social scenarios, and role-play | 90 min/week × 20 sessions | 20 | QoL | Kidscreen-27-P |
| 60 | Chien 2023 | Taiwan | RCT | 6 F / 35 M | 25.3 (4.5) | High-functioning ASD | Group-based PEERS® for Young Adults (PEERS-YA) | 90 min/week × 16 sessions | 16 | ANX | SIAS |
| 61 | Corbett 2023 | USA | RCT | 31 girls / 83 boys | 12.62 (1.86) | High-functioning ASD | SENSE Theatre – Peer-mediated, theater-based intervention | 180 min/week × 12 sessions | 12 | ANX | CASS |
| 62 | Özyurt 2017 | Turkey | RCT | 5girls / 7 boys | Not reported | Not reported | Individual therapeutic horseback riding (1 child/session), once per week, guided by certified instructor + volunteer team | 60 min/week × 8 weeks | 8 | QoL | PedsQL |
| 63 | Pahnke 2023 | Sweden | RCT | 10 F / 10 M | 38.4 (10.0) | High-functioning ASD | Group-based ACT (NeuroACT), 14 weekly 150-min sessions + 30-min homework guidance | 180 min/week × 14 sessions | 14 | QoL | QOLI |

| | | | | | | | | | | | |
|---|---|---|---|---|---|---|---|---|---|---|---|
| 64 | Storch 2015 | USA | RCT | 4girls / 12boys | 12.75 (1.24) | High-functioning ASD | Modular, individualized CBT (BIACA-based protocol) | 75 min/week (avg) × 16 sessions | 16 | ANX | PARS |
| 65 | White 2013 | USA | RCT | 4girls / 11boys | 14.17 (1.62) | High-functioning ASD | MASSI (Multimodal Anxiety and Social Skills Intervention) | 75 min/week (individual) + 75 min/week (group) × 14 sessions | 15 | ANX | PARS |
| 66 | Matthews 2025 | USA | RCT | 6girls / 13boys | 15.32 (1.60) | High-functioning ASD | Telehealth mindfulness-based group intervention (MINDful TIME), 8 weekly sessions + mobile app guided meditation + caregiver support | 90 min/week × 8 sessions | 8 | DEP | CDI-2 |
| 67 | Yang 2023 | Korea | RCT | 3F / 27M | 20.97 (5.06) | ASD without intellectual disability | App-based cognitive behavioural therapy (CBT) – HARU ASD program | 10–15 min/day × 7 days/week | 9.5 | ANX | STAI-S |

Note:ANX = Anxiety; DEP = Depression; QoL = Quality of Life;RCT = Randomized Controlled Trial; ASD = Autism Spectrum Disorder; SD = Standard Deviation;CTRL = Control; MBI = Mindfulness-Based Intervention; CBT = Cognitive Behavioral Therapy;BEHAVE = Behavioral and Functional Training; PHYS = Physical Activity Intervention;SENS = Sensory-Based Intervention; TAFI = Technology- and Family-Based Intervention;OTH = Other Therapies.

**Anxiety Measures:**PARS / PARS-A = Pediatric Anxiety Rating Scale;SCAS / SCAS-P / SCAS-C = Spence Children's Anxiety Scale (Parent / Child Version);STAI / STAI-S / STAI-C / STAI-T / STAI-2 / STAI-CH = State – Trait Anxiety Inventory (total, state, child, trait, version 2, child version);GAD-7 = Generalized Anxiety Disorder 7-item;BAI / MASC / MASC-P / MASC-2 = Beck Anxiety Inventory / Multidimensional Anxiety Scale for Children;SIAS = Social Interaction Anxiety Scale;ASR-AP = Adult Self-Report – Anxiety Problems;ADIS-P / ADIS-P PIR = Anxiety Disorders Interview Schedule – Parent Version / Parent Interview Rating;LSAS-SR = Liebowitz Social Anxiety Scale – Self-Report;DBC – A = Developmental Behavior Checklist – Anxiety subscale;CASS = Contextual Assessment of Social Skills;CASI-Anx = Child and Adolescent Symptom Inventory – Anxiety;ASC-ASD-P = Anxiety Scale for Children with Autism Spectrum Disorder – Parent Version.

**Depression Measures:**BDI / BDI-II = Beck Depression Inventory / Second Edition;CDI / CDI-2 = Children's Depression Inventory;PHQ-9 = Patient Health Questionnaire-9;DASS-Dep = Depression subscale of Depression Anxiety Stress Scale;ASR-DP = Adult Self-Report – Depression Problems;HADS-D = Hospital Anxiety and Depression Scale – Depression;SCL-90-R-d = Symptom Checklist-90-Revised – Depression;QIDS = Quick Inventory of Depressive Symptomatology.
**Quality of Life Measures:**WHOQOL-BREF = World Health Organization Quality of Life Scale – BREF;PedsQL = Pediatric Quality of Life Inventory;CHU-9D = Child Health Utility 9D;EQ-5D-5L = EuroQol 5 Dimensions 5 Levels;QOLI = Quality of Life Inventory;QOL-Q = Quality of Life Questionnaire;BBQ = Brunnsviken Brief Quality of Life Scale;Kidscreen-27-P = Kidscreen-27 Parent Version;FQOL = Family Quality of Life Scale.

## **Supplementary Table S4 –** Risk of bias table of included studies.

| Study | A | B | C | D | E | F | G |
|---|---|---|---|---|---|---|---|
| Braden 2021 | 🟢 | 🟢 | 🟢 | 🟢 | 🔴 | 🟢 | 🟢 |
| Hall 2020 | 🟢 | 🟢 | 🟡 | 🟢 | 🟢 | 🟢 | 🟢 |
| Clarke 2016 | 🟢 | 🟢 | 🟡 | 🟢 | 🔴 | 🟡 | 🔴 |
| Cook 2017 | 🟢 | 🟢 | 🟡 | 🟡 | 🟢 | 🟡 | 🟢 |
| Corbett 2016 | 🟢 | 🟢 | 🟡 | 🟢 | 🟢 | 🟢 | 🟢 |
| Pryor 2021 | 🟢 | 🟢 | 🟡 | 🟢 | 🟢 | 🟡 | 🟢 |
| Frolli 2021 | 🟢 | 🟢 | 🟡 | 🟡 | 🟢 | 🟡 | 🔴 |
| Gaigg 2020 | 🟢 | 🟢 | 🟡 | 🟡 | 🟢 | 🟡 | 🟢 |
| Villamisar 2010 | 🟢 | 🟢 | 🟢 | 🟢 | 🟢 | 🟡 | 🟢 |
| Guzick 2024 | 🟢 | 🟢 | 🟢 | 🟢 | 🟢 | 🟢 | 🟢 |
| Huntjens 2024 | 🟢 | 🟢 | 🟡 | 🟢 | 🟢 | 🟢 | 🟢 |
| Lee 2022 | 🟢 | 🟢 | 🟡 | 🟢 | 🟢 | 🟡 | 🟢 |
| Luxford 2017 | 🟢 | 🟢 | 🔴 | 🔴 | 🟢 | 🟢 | 🟢 |
| Lindor 2023 | 🟢 | 🟡 | 🟢 | 🟡 | 🟢 | 🟢 | 🟡 |
| McGillivray 2014 | 🔴 | 🟡 | 🔴 | 🟡 | 🟢 | 🟢 | 🟢 |
| McKenzie 2020 | 🟢 | 🟢 | 🟡 | 🟢 | 🟢 | 🟢 | 🟢 |
| Keehn 2013 | 🟢 | 🟡 | 🟡 | 🟢 | 🟢 | 🟢 | 🟢 |
| Mills 2020 | 🟢 | 🟡 | 🔴 | 🟢 | 🟢 | 🟢 | 🔴 |
| McVey 2016 | 🟡 | 🟡 | 🟢 | 🟡 | 🟢 | 🟢 | 🟢 |
| Sizoo 2017 | 🟡 | 🔴 | 🔴 | 🟢 | 🟢 | 🟢 | 🟢 |
| Padmanabha 2018 | 🟢 | 🟢 | 🔴 | 🟡 | 🟢 | 🟢 | 🟢 |
| Pagni 2023 | 🟡 | 🟡 | 🟢 | 🟢 | 🟡 | 🟢 | 🟢 |
| Pagni 2020 | 🟢 | 🟡 | 🟡 | 🟢 | 🟡 | 🟢 | 🟡 |
| Papadopoulos 2022 | 🟢 | 🟡 | 🟢 | 🟢 | 🔴 | 🟡 | 🟡 |
| Prasitwut 2024 | 🟢 | 🟢 | 🟢 | 🟡 | 🟡 | 🟢 | 🟢 |
| Quadt 2021 | 🟢 | 🟢 | 🔴 | 🟢 | 🟢 | 🟢 | 🟢 |
| Reaven 2012 | 🟢 | 🟡 | 🔴 | 🟢 | 🟢 | 🟢 | 🟢 |
| Russell 2020 | 🟢 | 🟢 | 🟡 | 🟡 | 🟢 | 🟢 | 🟡 |

| Study | A | B | C | D | E | F | G |
|---|---|---|---|---|---|---|---|
| Spek 2013 | Low | Low | Low | Unclear | Low | Low | Low |
| Storch 2013 | Low | Unclear | Unclear | Low | Low | Low | Low |
| Storch 2020 | Low | Unclear | High | Low | Low | Low | Low |
| Sung 2011 | Low | Unclear | High | Low | Low | Low | Low |
| Wijnhoven 2020 | Low | Low | Unclear | Unclear | Low | Low | Low |
| Wood 2009 | Low | Low | Unclear | Low | Low | Low | Low |
| Wood 2015 | Low | Unclear | Low | Unclear | Unclear | Low | Unclear |
| Westerberg 2023 | Low | Low | High | Unclear | Low | Low | Low |
| Wood 2019 | Low | Low | Unclear | Low | Low | Low | Low |
| Yoo 2014 | Low | Unclear | High | Unclear | Low | Low | Low |
| Yang 2023 | Low | Unclear | High | Low | Low | Low | Low |
| Adams 2024 | Low | Low | High | Low | Low | Low | Unclear |
| Akhani 2021 | Low | Unclear | High | Unclear | Low | Low | Low |
| Aljubour 2023 | Low | Unclear | Low | Low | Low | Low | Low |
| Chalfant 2006 | Low | Unclear | Unclear | Unclear | Low | Low | Unclear |
| Chien 2021 | Low | Unclear | Unclear | Unclear | Low | Low | Low |
| Conaughton 2017 | Low | Low | Unclear | Low | Low | Low | Low |
| Fatta 2024 | Low | Low | Unclear | Unclear | Low | Low | Low |
| Hawkins 2019 | Low | Unclear | Unclear | Unclear | Low | Low | Unclear |
| Gao 2024 | Low | Unclear | Unclear | Unclear | Low | Low | Low |
| Hesselmark 2014 | Low | Unclear | High | High | Low | Low | Unclear |
| Howells 2020 | High | High | Unclear | Unclear | Low | Low | Unclear |
| Ireri 2019 | Low | Unclear | Unclear | Unclear | Low | Low | Unclear |
| Kilburn 2020 | Low | Low | Unclear | Low | Low | Low | Low |
| McConachie 2014 | Low | Low | Unclear | Low | Low | Low | Low |
| Nadig 2018 | Low | Low | Unclear | Low | Low | Low | Low |
| Santomauro 2015 | Low | Unclear | Unclear | Unclear | Low | Low | Unclear |
| Wijker 2019 | Low | Low | Unclear | Unclear | Low | Low | Low |
| Gerber 2023 | Low | Low | Unclear | Low | Low | Low | Low |
| Backman 2024 | Low | Low | Low | Unclear | Low | Low | Low |
| Baghdadli 2013 | Low | Low | Low | Low | Low | Low | Low |
| Chien 2023 | Low | Unclear | Unclear | Unclear | Low | Low | Low |
| Corbett 2023 | Low | Low | Unclear | Low | Low | Low | Low |
| Ozyurt 2017 | Low | Unclear | High | Unclear | Low | Low | Unclear |
| Pahnke 2023 | Low | Unclear | High | Unclear | Low | Low | Low |
| Storch 2015 | Low | Low | High | Low | Low | Low | Low |
| White 2013 | Low | Low | High | Low | Low | Low | Low |
| Matthews 2025 | Low | Low | High | Unclear | Low | Low | Low |

Risk of bias: Low risk; Unclear; High risk

Note: A: Random sequence generation; B: Allocation concealment; C: Blinding of participants and personnelD: Blinding of outcome assessors; E: Incomplete outcome; F: Selective outcome reporting; G: Other risks of bias

**Supplementary Figure S1** – Risk of bias graph.

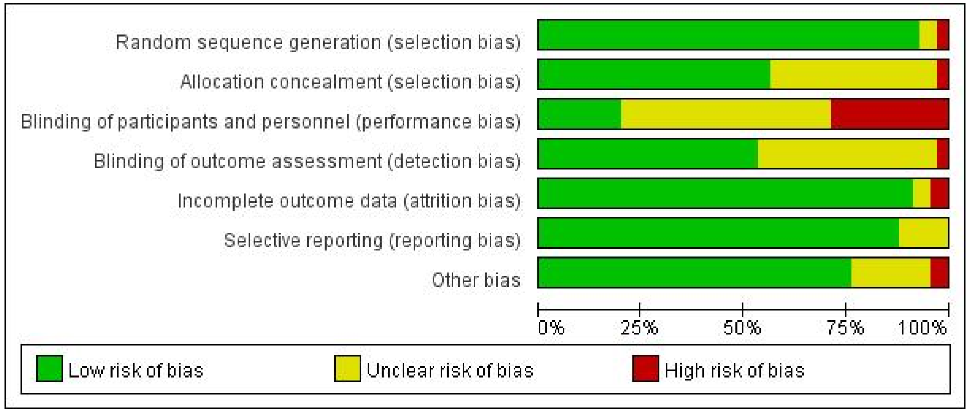


**Supplementary Figure S2** – Risk of bias summary.

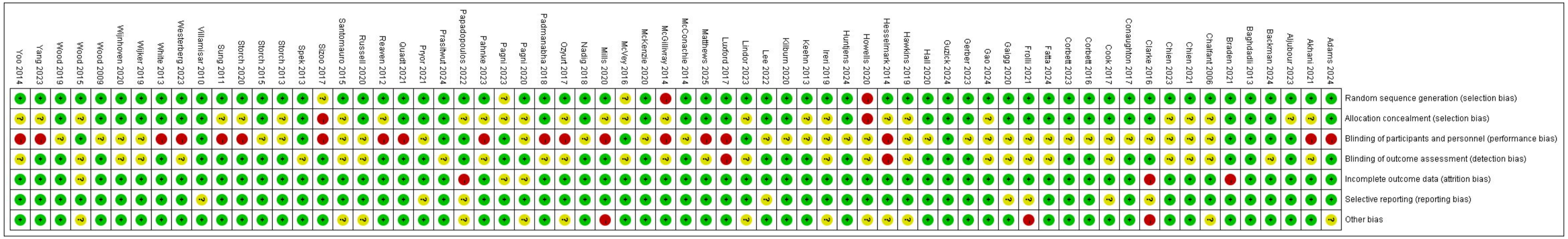

**Supplementary Figure S3 –** Anxiety: Pairwise meta-analysis.

| Study or Subgroup | Experimental Mean | Experimental SD | Experimental Total | Control Mean | Control SD | Control Total | Weight | Std. Mean Difference IV, Fixed, 95% CI |
|---|---|---|---|---|---|---|---|---|
| **3.1.1 CBT vs CTRL** | | | | | | | | |
| Backman 2024 | 10 | 4.02 | 24 | 10.04 | 4.33 | 24 | 2.0% | -0.01 [-0.58, 0.56] |
| Frolli 2021 | 14 | 1.27 | 27 | 20.93 | 1.01 | 27 | 0.4% | -5.95 [-7.23, -4.67] |
| Gaigg 2020 | 46.95 | 5.89 | 14 | 52.48 | 6.1 | 16 | 1.1% | -0.90 [-1.65, -0.14] |
| Pagni 2020 | 39.2 | 8.18 | 15 | 41 | 13.07 | 13 | 1.1% | -0.16 [-0.91, 0.58] |
| Sizoo 2017 | 9.5 | 3 | 32 | 10.9 | 4.4 | 27 | 2.3% | -0.37 [-0.89, 0.14] |
| Spek 2013 | 16.6 | 4.9 | 20 | 21.4 | 6.1 | 21 | 1.5% | -0.85 [-1.49, -0.21] |
| **Subtotal (95% CI)** | | | **132** | | | **128** | **8.4%** | **-0.67 [-0.94, -0.39]** |
| Heterogeneity: Chi² = 74.13, df = 5 (P < 0.00001); I² = 93% | | | | | | | | |
| Test for overall effect: Z = 4.79 (P < 0.00001) | | | | | | | | |
| **3.1.2 MBI vs CTRL** | | | | | | | | |
| Adams 2024 | 22.81 | 12.83 | 26 | 23.08 | 11.79 | 24 | 2.0% | -0.02 [-0.58, 0.53] |
| Chalfant 2006 | 13.79 | 10.96 | 28 | 41.37 | 9.09 | 19 | 1.0% | -2.64 [-3.45, -1.84] |
| Clarke 2016 | 27.9 | 9.6 | 14 | 34.1 | 8.4 | 14 | 1.1% | -0.67 [-1.43, 0.10] |
| Conaughton 2017 | 23.8 | 15.21 | 21 | 30.55 | 15.21 | 21 | 1.7% | -0.44 [-1.05, 0.18] |
| Cook 2017 | 66.07 | 10.17 | 14 | 74.17 | 10.21 | 12 | 1.0% | -0.77 [-1.57, 0.03] |
| Gaigg 2020a | 48.85 | 5.77 | 9 | 52.48 | 6.1 | 16 | 0.9% | -0.59 [-1.42, 0.25] |
| Huntjens 2024 | 38.7 | 12.8 | 57 | 40.6 | 12.8 | 56 | 4.6% | -0.15 [-0.52, 0.22] |
| Ireri 2019 | 6.58 | 2.17 | 20 | 6.35 | 1.86 | 20 | 1.6% | 0.11 [-0.51, 0.73] |
| Keehn 2013 | 20.08 | 11.34 | 12 | 31.7 | 13.36 | 10 | 0.8% | -0.91 [-1.80, -0.02] |
| Kilburn 2020 | 35.82 | 17.06 | 22 | 41.55 | 12.27 | 22 | 1.8% | -0.38 [-0.98, 0.22] |
| Luxford 2017 | 31.89 | 18.4 | 18 | 35.41 | 21.35 | 17 | 1.4% | -0.17 [-0.84, 0.49] |
| McConachie 2014 | 38.35 | 16.23 | 17 | 42 | 19.53 | 15 | 1.3% | -0.20 [-0.90, 0.50] |
| McGillivray 2014 | 14.2 | 8.09 | 15 | 12.58 | 6.88 | 12 | 1.1% | 0.21 [-0.55, 0.97] |
| Pryor 2021 | 3.83 | 1.26 | 12 | 6.17 | 1.34 | 12 | 0.7% | -1.74 [-2.70, -0.77] |
| Reaven 2012 | 2.55 | 2.5 | 20 | 4.61 | 1.7 | 23 | 1.5% | -0.96 [-1.59, -0.32] |
| Russell 2020 | 8.3 | 4.5 | 28 | 10.6 | 5.7 | 17 | 1.7% | -0.45 [-1.06, 0.16] |
| Storch 2013 | 11.58 | 3.15 | 24 | 16.05 | 3.22 | 21 | 1.4% | -1.38 [-2.04, -0.72] |
| Storch 2015 | 10.94 | 3.91 | 16 | 13.93 | 3.56 | 15 | 1.2% | -0.78 [-1.51, -0.04] |
| Storch 2020 | 7.79 | 5.37 | 14 | 15.06 | 3.21 | 18 | 0.9% | -1.66 [-2.48, -0.83] |
| Sung 2011 | 26.54 | 15.57 | 28 | 27.62 | 13.57 | 29 | 2.3% | -0.07 [-0.59, 0.45] |
| Westerberg 2023 | 10.86 | 3.83 | 42 | 10.33 | 3.74 | 42 | 3.4% | 0.14 [-0.29, 0.57] |
| White 2013 | 14 | 3.44 | 15 | 15.47 | 5.26 | 15 | 1.2% | -0.32 [-1.04, 0.40] |
| Wood 2009 | 58.48 | 14.72 | 14 | 76.57 | 14.65 | 22 | 1.2% | -1.21 [-1.94, -0.47] |
| Wood 2015 | 11.62 | 3.26 | 19 | 14.04 | 2.64 | 14 | 1.2% | -0.78 [-1.50, -0.06] |
| Wood 2019 | 2.43 | 0.7 | 59 | 2.93 | 0.59 | 18 | 2.1% | -0.73 [-1.27, -0.19] |
| **Subtotal (95% CI)** | | | **564** | | | **504** | **39.0%** | **-0.49 [-0.62, -0.37]** |
| Heterogeneity: Chi² = 83.46, df = 24 (P < 0.00001); I² = 71% | | | | | | | | |
| Test for overall effect: Z = 7.66 (P < 0.00001) | | | | | | | | |
| **3.1.3 BEHAVE vs CTRL** | | | | | | | | |
| Chien 2021 | 39.1 | 15.5 | 36 | 45.7 | 15.7 | 36 | 2.9% | -0.42 [-0.89, 0.05] |
| Corbett 2023 | 4.03 | 1.59 | 93 | 4.19 | 1.53 | 114 | 8.3% | -0.10 [-0.38, 0.17] |
| Fatta 2024 | 55.61 | 12.91 | 18 | 55.26 | 12.42 | 19 | 1.5% | 0.03 [-0.62, 0.67] |
| Hall 2020 | 54.79 | 6.05 | 16 | 60.08 | 9.94 | 16 | 1.2% | -0.63 [-1.34, 0.09] |
| McVey 2016 | 50.96 | 31.99 | 24 | 65.58 | 24.6 | 23 | 1.8% | -0.50 [-1.08, 0.08] |
| Pagni 2023 | 38.03 | 12.52 | 36 | 38.42 | 10.37 | 38 | 3.0% | -0.03 [-0.49, 0.42] |
| Wood 2019a | 2.13 | 0.91 | 66 | 2.93 | 0.59 | 18 | 2.1% | -0.93 [-1.47, -0.39] |
| **Subtotal (95% CI)** | | | **289** | | | **264** | **20.9%** | **-0.28 [-0.45, -0.10]** |
| Heterogeneity: Chi² = 10.94, df = 6 (P = 0.09); I² = 45% | | | | | | | | |
| Test for overall effect: Z = 3.14 (P = 0.002) | | | | | | | | |
| **3.1.4 PHYS vs CTRL** | | | | | | | | |
| Howells 2020 | 60.95 | 9.54 | 19 | 66.67 | 11.76 | 21 | 1.6% | -0.52 [-1.15, 0.11] |
| **Subtotal (95% CI)** | | | **19** | | | **21** | **1.6%** | **-0.52 [-1.15, 0.11]** |
| Heterogeneity: Not applicable | | | | | | | | |
| Test for overall effect: Z = 1.61 (P = 0.11) | | | | | | | | |
| **3.1.5 SENS vs CTRL** | | | | | | | | |
| Aljubour 2023 | 13.84 | 8.6 | 32 | 18.06 | 12.16 | 32 | 2.5% | -0.40 [-0.89, 0.10] |
| Hawkins 2019 | 33.64 | 8.37 | 11 | 27.5 | 6.78 | 14 | 0.9% | 0.79 [-0.03, 1.62] |
| Mills 2020 | 54.88 | 5.17 | 8 | 61.25 | 9.51 | 8 | 0.6% | -0.79 [-1.82, 0.24] |
| Quadt 2021 | 54.3 | 11.3 | 50 | 55.7 | 10.7 | 47 | 3.9% | -0.13 [-0.52, 0.27] |
| **Subtotal (95% CI)** | | | **101** | | | **101** | **8.0%** | **-0.16 [-0.44, 0.12]** |
| Heterogeneity: Chi² = 7.42, df = 3 (P = 0.06); I² = 60% | | | | | | | | |
| Test for overall effect: Z = 1.09 (P = 0.28) | | | | | | | | |
| **3.1.6 TAFI vs CTRL** | | | | | | | | |
| Backman 2024a | 9.33 | 4.06 | 60 | 10.04 | 4.33 | 24 | 2.8% | -0.17 [-0.64, 0.30] |
| Guzick 2024 | 11.53 | 4.6 | 28 | 10.32 | 4.68 | 29 | 2.3% | 0.26 [-0.26, 0.78] |
| Lee 2022 | 14.5 | 10.05 | 12 | 12.9 | 9.01 | 12 | 1.0% | 0.16 [-0.64, 0.96] |
| Wijnhoven 2020 | 20.08 | 7.18 | 53 | 23.92 | 9.34 | 56 | 4.3% | -0.46 [-0.84, -0.08] |
| Yang 2023 | 38.53 | 10.12 | 15 | 50.73 | 11.13 | 15 | 1.0% | -1.12 [-1.89, -0.34] |
| **Subtotal (95% CI)** | | | **168** | | | **136** | **11.4%** | **-0.25 [-0.48, -0.02]** |
| Heterogeneity: Chi² = 10.65, df = 4 (P = 0.03); I² = 62% | | | | | | | | |
| Test for overall effect: Z = 2.09 (P = 0.04) | | | | | | | | |
| **3.1.7 OTH vs CTRL** | | | | | | | | |
| Corbett 2016 | 34.41 | 9.14 | 17 | 40.62 | 10.25 | 13 | 1.1% | -0.63 [-1.37, 0.11] |
| Gerber 2023 | 61.53 | 10.44 | 20 | 64.95 | 14.02 | 20 | 1.6% | -0.27 [-0.89, 0.35] |
| Papadopoulos 2022 | 6.54 | 2.37 | 98 | 7.6 | 2.26 | 105 | 8.0% | -0.46 [-0.74, -0.18] |
| **Subtotal (95% CI)** | | | **135** | | | **138** | **10.8%** | **-0.45 [-0.69, -0.21]** |
| Heterogeneity: Chi² = 0.54, df = 2 (P = 0.76); I² = 0% | | | | | | | | |
| Test for overall effect: Z = 3.64 (P = 0.0003) | | | | | | | | |
| **Total (95% CI)** | | | **1408** | | | **1292** | **100.0%** | **-0.40 [-0.48, -0.32]** |
| Heterogeneity: Chi² = 199.68, df = 50 (P < 0.00001); I² = 75% | | | | | | | | |
| Test for overall effect: Z = 10.01 (P < 0.00001) | | | | | | | | |
| Test for subgroup differences: Chi² = 12.54, df = 6 (P = 0.05), I² = 52.1% | | | | | | | | |

Std. Mean Difference
IV, Fixed, 95% CI

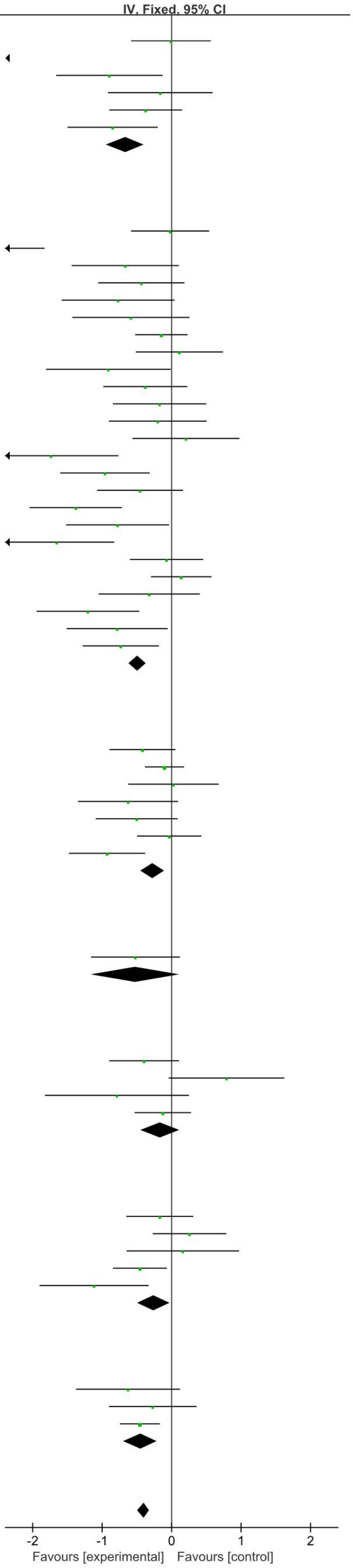

# **Supplementary Figure S4 –** Depression: Pairwise meta-analysis.

| Study or Subgroup | Experimental Mean | Experimental SD | Experimental Total | Control Mean | Control SD | Control Total | Weight | Std. Mean Difference IV, Fixed, 95% CI |
|---|---|---|---|---|---|---|---|---|
| **4.1.1 MBI vs CTRL** | | | | | | | | |
| Backman 2024b | 7.13 | 4.09 | 24 | 6.13 | 4.39 | 24 | 5.2% | 0.23 [-0.34, 0.80] |
| Frolli 2021b | 14.11 | 0.75 | 27 | 14.11 | 0.75 | 27 | 5.9% | 0.00 [-0.53, 0.53] |
| Pagni 2020b | 8.73 | 6.05 | 15 | 10.15 | 7.35 | 13 | 3.0% | -0.21 [-0.95, 0.54] |
| Sizoo 2017b | 7.3 | 5.6 | 32 | 6.9 | 5 | 32 | 7.0% | 0.07 [-0.42, 0.56] |
| Spek 2013b | 28.3 | 8.6 | 20 | 37.7 | 11.6 | 21 | 4.0% | -0.90 [-1.55, -0.25] |
| **Subtotal (95% CI)** | | | **118** | | | **117** | **25.0%** | **-0.10 [-0.36, 0.16]** |
| Heterogeneity: Chi$^2$ = 7.90, df = 4 (P = 0.10); I$^2$ = 49% | | | | | | | | |
| Test for overall effect: Z = 0.76 (P = 0.45) | | | | | | | | |
| **4.1.2 CBT vs CTRL** | | | | | | | | |
| Hesselmark 2014 | 9.2 | 6.9 | 0 | 11.1 | 8.9 | 0 | | Not estimable |
| Huntjens 2024b | 15.78 | 7.56 | 31 | 20 | 9.21 | 26 | 6.0% | -0.50 [-1.03, 0.03] |
| McGillivray 2014b | 16.87 | 10.8 | 15 | 19.2 | 9.45 | 10 | 2.6% | -0.22 [-1.02, 0.58] |
| Russell 2020b | 18.7 | 10.3 | 28 | 27.6 | 13.6 | 16 | 4.1% | -0.75 [-1.39, -0.12] |
| Santomauro 2015 | 17.2 | 8.95 | 10 | 23.93 | 11.58 | 10 | 2.1% | -0.62 [-1.53, 0.28] |
| Westerberg 2023b | 6.7 | 3.41 | 42 | 6.86 | 3.42 | 42 | 9.1% | -0.05 [-0.47, 0.38] |
| **Subtotal (95% CI)** | | | **126** | | | **104** | **23.9%** | **-0.35 [-0.61, -0.09]** |
| Heterogeneity: Chi$^2$ = 4.24, df = 4 (P = 0.37); I$^2$ = 6% | | | | | | | | |
| Test for overall effect: Z = 2.59 (P = 0.010) | | | | | | | | |
| **4.1.3 BEHAVE vs CTRL** | | | | | | | | |
| Fatta 2024b | 52 | 9.84 | 18 | 48.95 | 7.14 | 19 | 4.0% | 0.35 [-0.30, 1.00] |
| Hall 2020b | 58.43 | 8.67 | 16 | 66.17 | 11.02 | 16 | 3.2% | -0.76 [-1.48, -0.04] |
| Pagni 2023b | 10.35 | 9.05 | 37 | 11.05 | 9.86 | 39 | 8.3% | -0.07 [-0.52, 0.38] |
| Schiltz 2018 | 43.05 | 5.52 | 24 | 49.41 | 8.47 | 25 | 4.8% | -0.87 [-1.46, -0.28] |
| Yoo 2014 | 18.14 | 8.62 | 24 | 16.78 | 11.27 | 23 | 5.1% | 0.13 [-0.44, 0.71] |
| **Subtotal (95% CI)** | | | **119** | | | **122** | **25.3%** | **-0.21 [-0.46, 0.05]** |
| Heterogeneity: Chi$^2$ = 11.68, df = 4 (P = 0.02); I$^2$ = 66% | | | | | | | | |
| Test for overall effect: Z = 1.57 (P = 0.12) | | | | | | | | |
| **4.1.4 PHYS vs CTRL** | | | | | | | | |
| Howells 2020b | 64.42 | 9.58 | 19 | 64.52 | 10.42 | 21 | 4.3% | -0.01 [-0.63, 0.61] |
| **Subtotal (95% CI)** | | | **19** | | | **21** | **4.3%** | **-0.01 [-0.63, 0.61]** |
| Heterogeneity: Not applicable | | | | | | | | |
| Test for overall effect: Z = 0.03 (P = 0.98) | | | | | | | | |
| **4.1.5 TAFI vs CTRL** | | | | | | | | |
| Backman 2024c | 6.97 | 4.45 | 60 | 6.13 | 4.39 | 24 | 7.4% | 0.19 [-0.29, 0.66] |
| Matthews 2025 | 59.53 | 7.86 | 19 | 66.1 | 12.21 | 21 | 4.1% | -0.62 [-1.26, 0.02] |
| **Subtotal (95% CI)** | | | **79** | | | **45** | **11.5%** | **-0.10 [-0.48, 0.28]** |
| Heterogeneity: Chi$^2$ = 3.98, df = 1 (P = 0.05); I$^2$ = 75% | | | | | | | | |
| Test for overall effect: Z = 0.52 (P = 0.60) | | | | | | | | |
| **4.1.6 OTH vs CTRL** | | | | | | | | |
| Gerber 2023b | 54.35 | 11.27 | 20 | 60.68 | 13.2 | 20 | 4.2% | -0.51 [-1.14, 0.13] |
| Wijker 2019 | 15.62 | 13.7 | 27 | 20 | 13.7 | 26 | 5.7% | -0.31 [-0.86, 0.23] |
| **Subtotal (95% CI)** | | | **47** | | | **46** | **9.9%** | **-0.40 [-0.81, 0.02]** |
| Heterogeneity: Chi$^2$ = 0.20, df = 1 (P = 0.65); I$^2$ = 0% | | | | | | | | |
| Test for overall effect: Z = 1.89 (P = 0.06) | | | | | | | | |
| **Total (95% CI)** | | | **508** | | | **455** | **100.0%** | **-0.21 [-0.34, -0.08]** |
| Heterogeneity: Chi$^2$ = 31.27, df = 19 (P = 0.04); I$^2$ = 39% | | | | | | | | |
| Test for overall effect: Z = 3.21 (P = 0.001) | | | | | | | | |
| Test for subgroup differences: Chi$^2$ = 3.27, df = 5 (P = 0.66), I$^2$ = 0% | | | | | | | | |

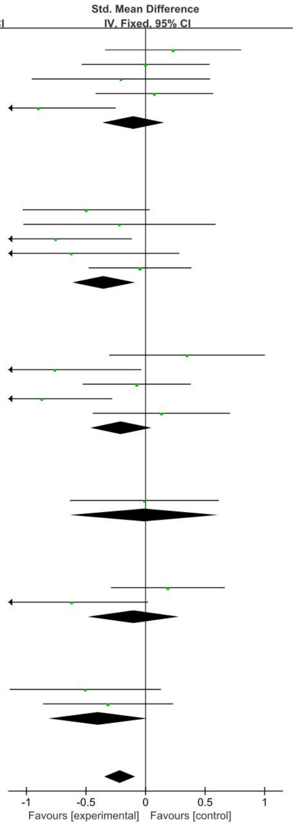


# **Supplementary Figure S5 –** Quality of Life: Pairwise meta-analysis.

| Study or Subgroup | Experimental Mean | Experimental SD | Experimental Total | Control Mean | Control SD | Control Total | Weight | Std. Mean Difference IV, Fixed, 95% CI |
|---|---|---|---|---|---|---|---|---|
| **2.1.1 MBI vs CTRL** | | | | | | | | |
| Braden 2022 | 14.1 | 5.05 | 27 | 14.45 | 6.18 | 28 | 6.3% | -0.06 [-0.59, 0.47] |
| Backman 2024d | 45 | 19.24 | 24 | 42.33 | 17.4 | 24 | 5.5% | 0.14 [-0.42, 0.71] |
| Pahnke 2023 | 1.67 | 1.4 | 17 | 0.12 | 1.9 | 19 | 3.7% | 0.90 [0.21, 1.59] |
| **Subtotal (95% CI)** | | | **68** | | | **71** | **15.5%** | **0.24 [-0.10, 0.58]** |
| Heterogeneity: Chi$^2$ = 4.87, df = 2 (P = 0.09); I$^2$ = 59% | | | | | | | | |
| Test for overall effect: Z = 1.40 (P = 0.16) | | | | | | | | |
| **2.1.2 CBT vs CTRL** | | | | | | | | |
| Westerberg 2023c | 59.26 | 12.96 | 42 | 62.48 | 10.67 | 41 | 9.4% | -0.27 [-0.70, 0.16] |
| Russell 2020c | 0.69 | 0.24 | 28 | 0.66 | 0.19 | 16 | 4.7% | 0.13 [-0.48, 0.75] |
| Huntjens 2024c | 3.91 | 1.07 | 57 | 3.66 | 0.9 | 56 | 12.8% | 0.25 [-0.12, 0.62] |
| Hesselmark 2014c | 0.64 | 1.9 | 57 | 0.01 | 1.62 | 56 | 12.7% | 0.35 [-0.02, 0.73] |
| **Subtotal (95% CI)** | | | **184** | | | **169** | **39.6%** | **0.15 [-0.06, 0.36]** |
| Heterogeneity: Chi$^2$ = 5.04, df = 3 (P = 0.17); I$^2$ = 41% | | | | | | | | |
| Test for overall effect: Z = 1.37 (P = 0.17) | | | | | | | | |
| **2.1.3 BEHAVE vs CTRL** | | | | | | | | |
| Nadig 2018 | 22.53 | 3.83 | 17 | 20.89 | 4.17 | 19 | 4.0% | 0.40 [-0.26, 1.06] |
| **Subtotal (95% CI)** | | | **17** | | | **19** | **4.0%** | **0.40 [-0.26, 1.06]** |
| Heterogeneity: Not applicable | | | | | | | | |
| Test for overall effect: Z = 1.18 (P = 0.24) | | | | | | | | |
| **2.1.4 PHYS vs CTRL** | | | | | | | | |
| Baghdadli 2013 | 39.1 | 6.6 | 7 | 36.3 | 4.9 | 6 | 1.4% | 0.44 [-0.67, 1.55] |
| Lindor 2023 | 62.23 | 9.42 | 8 | 55.21 | 10.46 | 12 | 2.1% | 0.67 [-0.26, 1.59] |
| Ozyurt 2017 | 82.08 | 22.59 | 12 | 61.08 | 18.98 | 12 | 2.4% | 0.97 [0.12, 1.83] |
| Villamisar 2010 | 63.62 | 8.99 | 37 | 55.29 | 3.45 | 34 | 6.8% | 1.19 [0.68, 1.70] |
| **Subtotal (95% CI)** | | | **64** | | | **64** | **12.7%** | **0.98 [0.61, 1.35]** |
| Heterogeneity: Chi$^2$ = 2.01, df = 3 (P = 0.57); I$^2$ = 0% | | | | | | | | |
| Test for overall effect: Z = 5.17 (P < 0.00001) | | | | | | | | |
| **2.1.5 SENS vs CTRL** | | | | | | | | |
| Prasitwut 2024 | 31 | 18.52 | 11 | 36.3 | 13.9 | 12 | 2.6% | -0.31 [-1.14, 0.51] |
| Padmanabha 2018 | 76.99 | 7.8 | 21 | 72.17 | 8.4 | 19 | 4.4% | 0.58 [-0.05, 1.22] |
| **Subtotal (95% CI)** | | | **32** | | | **31** | **6.9%** | **0.25 [-0.25, 0.75]** |
| Heterogeneity: Chi$^2$ = 2.86, df = 1 (P = 0.09); I$^2$ = 65% | | | | | | | | |
| Test for overall effect: Z = 0.97 (P = 0.33) | | | | | | | | |
| **2.1.6 TAFI vs CTRL** | | | | | | | | |
| McKenzie 2020 | 32.5 | 7.2 | 22 | 33 | 7.9 | 12 | 3.6% | -0.07 [-0.77, 0.64] |
| Backman 2024e | 45.51 | 20.69 | 60 | 42.33 | 17.4 | 24 | 7.8% | 0.16 [-0.32, 0.63] |
| Akhani 2021 | 90.94 | 10.28 | 19 | 77.76 | 10.95 | 22 | 3.9% | 1.21 [0.54, 1.89] |
| Gao 2024 | 65.11 | 3.01 | 35 | 60.57 | 2.59 | 35 | 6.0% | 1.60 [1.06, 2.14] |
| **Subtotal (95% CI)** | | | **136** | | | **93** | **21.2%** | **0.72 [0.43, 1.01]** |
| Heterogeneity: Chi$^2$ = 22.34, df = 3 (P < 0.0001); I$^2$ = 87% | | | | | | | | |
| Test for overall effect: Z = 4.90 (P < 0.00001) | | | | | | | | |
| **Total (95% CI)** | | | **501** | | | **447** | **100.0%** | **0.41 [0.27, 0.54]** |
| Heterogeneity: Chi$^2$ = 57.99, df = 17 (P < 0.00001); I$^2$ = 71% | | | | | | | | |
| Test for overall effect: Z = 6.01 (P < 0.00001) | | | | | | | | |
| Test for subgroup differences: Chi$^2$ = 20.86, df = 5 (P = 0.0009), I$^2$ = 76.0% | | | | | | | | |

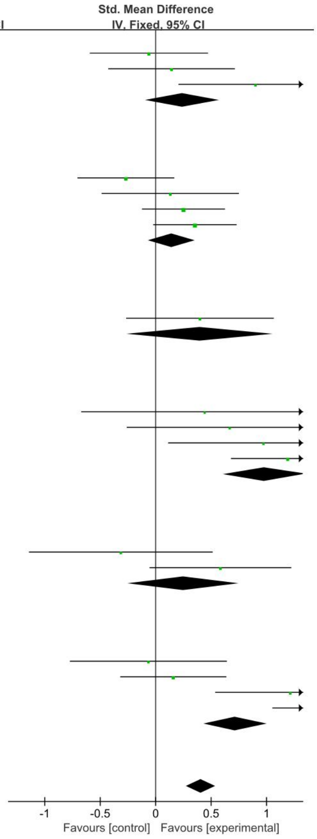

**Supplementary Figure S6 –** Anxiety: network evidence plot.

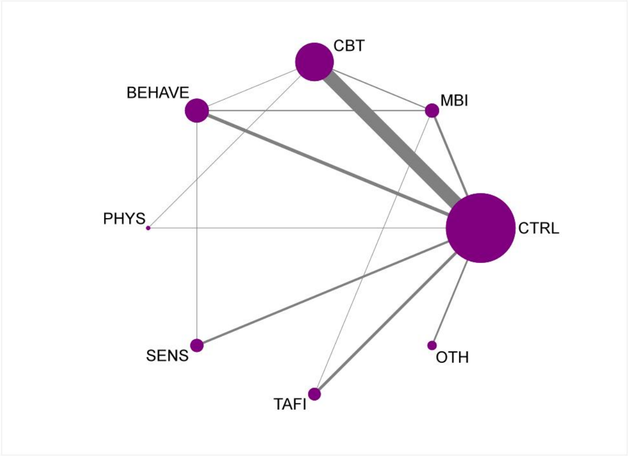


**Supplementary Figure S7** – Anxiety:Contribution plot of the network.

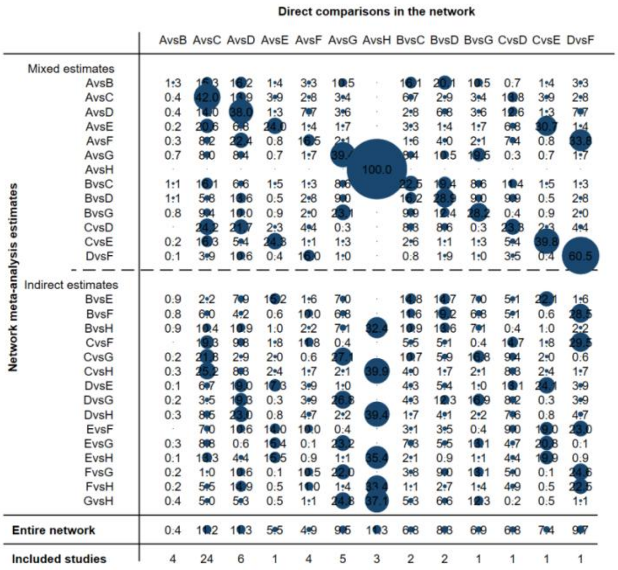


**Supplementary Figure S8 –** Anxiety: comparison-adjusted funnel plot.

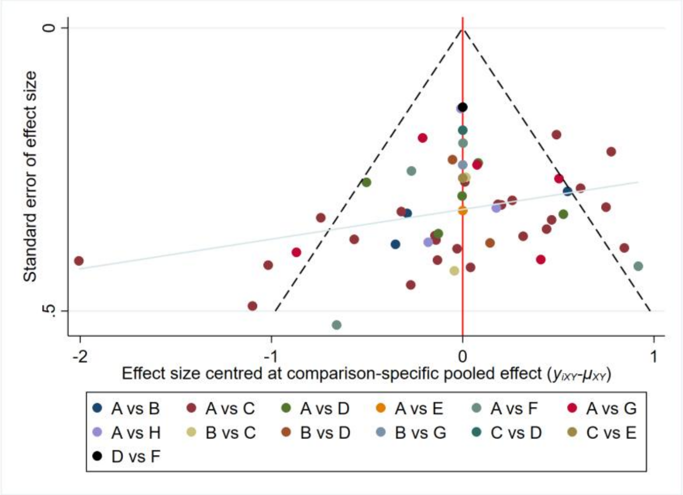

**Supplementary Table S5 –** Anxiety: Loop Inconsistency Test.

| | **Results of loop inconsistency test** | | | | | |
|---|---|---|---|---|---|---|
| Loop | IF | seIF | z_value | p_value | CI_95 | Loop_Heterog_tau2 |
| CTRL-MBI-TAFI | 1.613 | 2.232 | 0.723 | **0.470** | (0.00,5.99) | 1.325 |
| CTRL-MBI-BEHAVE | 1.071 | 1.354 | 0.791 | **0.429** | (0.00,3.73) | 0.710 |
| CTRL-MBI-CBT | 0.551 | 0.913 | 0.604 | **0.546** | (0.00,2.34) | 0.522 |
| CTRL-CBT-BEHAVE | 0.515 | 0.603 | 0.854 | **0.393** | (0.00,1.70) | 0.227 |
| CTRL-BEHAVE-SENS | 0.258 | 0.405 | 0.636 | **0.525** | (0.00,1.05) | 0.061 |
| CTRL-CBT-PHYS | 0.044 | 0.897 | 0.049 | **0.961** | (0.00,1.80) | 0.269 |
| MBI-CBT-BEHAVE | 0.040 | 0.350 | 0.114 | **0.910** | (0.00,0.73) | 0.00 |

**Supplementary Table S6 –** Anxiety: Global Inconsistency Test.

| | | |
|---|---|---|
| chi2( 10) | = | 12.74 |
| Prob > chi2 | = | **0.2388** |

**Supplementary Table S7 –** Anxiety: Local Inconsistency Test.

| Side | Direct | | Indirect | | Difference | | |
|---|---|---|---|---|---|---|---|
| | Coef. | Std. Err. | Coef. | Std. Err. | Coef. | Std. Err. | P>z |
| A B | -1.507899 | 0.3934741 | -0.7003413 | 0.4149268 | -0.8075579 | 0.5707876 | **0.157** |
| A C | -0.6500084 | 0.1537094 | -0.6036929 | 0.5676328 | -0.0463155 | 0.5875927 | **0.937** |
| A D | -0.4934779 | 0.2955516 | -0.900831 | 0.451958 | 0.4073531 | 0.5417269 | **0.452** |
| A E | -0.5207521 | 0.7391098 | -0.5749907 | 0.7315023 | 0.0542386 | 1.039894 | **0.958** |
| A F | -0.122411 | 0.375446 | -0.5583095 | 0.7252969 | 0.4358985 | 0.8167086 | **0.594** |
| A G * | -0.2548967 | 0.3120324 | -2.91228 | 1.338975 | 2.657384 | 1.37853 | **0.054** |
| A H | . | . | . | . | . | . | . |
| B C | 0.3429082 | 0.5311825 | 0.5642551 | 0.3757273 | -0.2213469 | 0.6518401 | **0.734** |
| B D | 0.0561766 | 0.5130556 | 0.824152 | 0.4222852 | -0.7679754 | 0.664284 | **0.248** |
| B G | -0.1398544 | 0.6858721 | 1.140566 | 0.4771247 | -1.28042 | 0.8364361 | **0.126** |
| C D | -0.3759033 | 0.6837002 | 0.1083512 | 0.3004921 | -0.4842545 | 0.7468044 | **0.517** |
| C E | 0.0730037 | 0.7159195 | 0.1272766 | 0.7542132 | -0.0542729 | 1.039893 | **0.958** |
| D F | 0.1023688 | 0.6771533 | 0.5382884 | 0.4566016 | -0.4359196 | 0.8167139 | **0.594** |

**Supplementary Table S8 –** Depression: Loop Inconsistency Test.

| | **Results of loop inconsistency test** | | | | | |
|---|---|---|---|---|---|---|
| Loop | IF | seIF | z_value | p_value | CI_95 | Loop_Heterog_tau2 |
| CTRL-CBT-PHYS | 0.233 | 0.433 | 0.538 | 0.591 | (0.00,1.08) | 0 |
| CTRL-MBI-BEHAVE | 0.122 | 0.577 | 0.212 | 0.832 | (0.00,1.25) | 0.205 |
| CTRL-MBI-CBT | 0.06 | 0.479 | 0.124 | 0.901 | (0.00,1.00) | 0.071 |
| CTRL-MBI-TAFI | . | . | . | . | (.,.) | 0 |

**Supplementary Table S9 –** Depression: Global Inconsistency Test.

| | | |
|---|---|---|
| chi2( 5) | = | 2.67 |
| Prob > chi2 | = | **0.7502** |

**Supplementary Table S10 –** Depression: Local Inconsistency Test.

| Side | Direct | | Indirect | | Difference | | |
|---|---|---|---|---|---|---|---|
| | Coef. | Std. Err. | Coef. | Std. Err. | Coef. | Std. Err. | P>z |
| A B | -0.1872827 | 0.231384 | -0.3549645 | 0.258023 | 0.1676819 | 0.3458172 | **0.628** |
| A C | -0.3222849 | 0.1828458 | -0.3965807 | 0.3366064 | 0.0742958 | 0.3824331 | **0.846** |
| A D | -0.275274 | 0.2109318 | -0.2198346 | 0.3464371 | -0.0554394 | 0.405599 | **0.891** |
| A E | -0.0097708 | 0.415994 | 0.1744063 | 0.4166738 | -0.1841771 | 0.5887852 | **0.754** |
| A F * | -0.1508868 | 0.2716525 | -0.8743174 | 0.7601092 | 0.7234306 | 0.8228984 | **0.379** |
| A G | . | . | . | . | . | . | **.** |
| B C | -0.0744191 | 0.3727891 | -0.0774439 | 0.2590648 | 0.0030248 | 0.4539673 | **0.995** |
| B D | 0.029513 | 0.2842783 | -0.0259222 | 0.2897927 | 0.0554352 | 0.4055998 | **0.891** |
| B F | 0.0152531 | 0.3645251 | 0.0144677 | 0.4344048 | 0.0007854 | 0.5765476 | **0.999** |
| C E | 0.498258 | 0.3819608 | 0.3140875 | 0.4480781 | 0.1841705 | 0.5887848 | **0.754** |

**Supplementary Table S11 –** Quality of Life: Loop Inconsistency Test.

| | **Results of loop inconsistency test** | | | | | |
|---|---|---|---|---|---|---|
| Loop | IF | seIF | z_value | p_value | CI_95 | Loop_Heterog_tau2 |
| CBT-CTRL-PHYS | 9.131 | 1.655 | 5.516 | 0 | (5.89,12.38) | 0.021 |
| BEHAVE-CTRL-PHYS | 4.319 | 4.854 | 0.89 | 0.374 | (0.00,13.83) | 2.422 |
| CTRL-MBI-TAFI | 3.592 | 7.592 | 0.473 | 0.636 | (0.00,18.47) | 6.093 |

**Supplementary Table S12 –** Quality of Life: Global Inconsistency Test.

| | | |
|---|---|---|
| chi2( 4) | = | 5.03 |
| Prob > chi2 | = | **0.2839** |

**Supplementary Table S13 –** Quality of Life: Local Inconsistency Test

| Side | Direct | | Indirect | | Difference | | |
|---|---|---|---|---|---|---|---|
| | Coef. | Std. Err. | Coef. | Std. Err. | Coef. | Std. Err. | P>z |
| A B * | 0.2990945 | 0.3390634 | 1.719411 | 1.177707 | -1.420316 | 1.225847 | **0.247** |
| A C | 0.0367686 | 0.2920079 | 1.328629 | 0.6060192 | -1.291861 | 0.6727048 | **0.055** |
| A D | 0.4024032 | 0.6809308 | 0.2303785 | 0.8503419 | 0.1720248 | 1.08938 | **0.875** |
| A E | 0.9813063 | 0.348636 | 0.012075 | 0.5202288 | 0.9692312 | 0.6273153 | **0.122** |

| | | | | | | | |
|---|---|---|---|---|---|---|---|
| A F | . | . | . | . | . | . | **.** |
| A G * | 0.7322039 | 0.3135809 | 0.6285961 | 1.38547 | 0.1036078 | 1.425393 | **0.942** |
| B G | 0.0067323 | 0.5787921 | 0.6083687 | 0.5593714 | -0.6016364 | 0.8038783 | **0.454** |
| C E | -0.3612988 | 0.5154581 | 0.9305946 | 0.4322521 | -1.291893 | 0.6727101 | **0.055** |
| D E | 0.4382428 | 0.7818067 | 0.2662319 | 0.7586533 | 0.1720109 | 1.089395 | **0.875** |

**Supplementary Figure S9 –** Anxiety: network forest

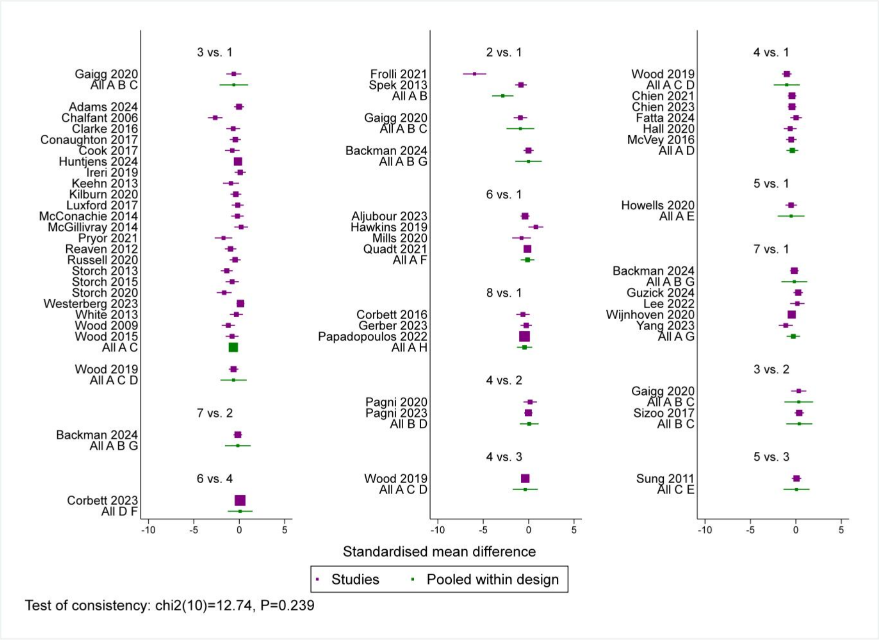

**Supplementary Table S14 –** Anxiety: league table.

| | | | | | | | |
|---|---|---|---|---|---|---|---|
| **MBI** | 0.49 (-0.10,1.07) | 0.52 (-0.12,1.15) | 0.58 (-0.55,1.72) | **0.92 (0.08,1.75)** | 0.73 (-0.05,1.51) | 0.68 (-0.30,1.66) | **1.13 (0.57,1.69)** |
| -0.49 (-1.07,0.10) | **CBT** | 0.03 (-0.50,0.56) | 0.10 (-0.90,1.10) | 0.43 (-0.27,1.13) | 0.24 (-0.43,0.91) | 0.20 (-0.66,1.05) | **0.65 (0.36,0.93)** |
| -0.52 (-1.15,0.12) | -0.03 (-0.56,0.50) | **BEHAVE** | 0.07 (-1.03,1.17) | 0.40 (-0.33,1.13) | 0.21 (-0.55,0.98) | 0.17 (-0.77,1.10) | **0.62 (0.14,1.09)** |
| -0.58 (-1.72,0.55) | -0.10 (-1.10,0.90) | -0.07 (-1.17,1.03) | **PHYS** | 0.33 (-0.86,1.52) | 0.15 (-1.02,1.32) | 0.10 (-1.18,1.38) | 0.55 (-0.45,1.55) |
| **-0.92(-1.75,-0.08)** | -0.43 (-1.13,0.27) | -0.40 (-1.13,0.33) | -0.33 (-1.52,0.86) | **SENS** | -0.19 (-1.07,0.70) | -0.23 (-1.26,0.80) | 0.21 (-0.43,0.86) |
| -0.73 (-1.51,0.05) | -0.24 (-0.91,0.43) | -0.21 (-0.98,0.55) | -0.15 (-1.32,1.02) | 0.19 (-0.70,1.07) | **TAFI** | -0.05 (-1.06,0.96) | 0.40 (-0.21,1.01) |
| -0.68 (-1.66,0.30) | -0.20 (-1.05,0.66) | -0.17 (-1.10,0.77) | -0.10 (-1.38,1.18) | 0.23 (-0.80,1.26) | 0.05 (-0.96,1.06) | **OTH** | 0.45 (-0.36,1.25) |
| **-1.13(-1.69,-0.57)** | **-0.65 (-0.93,-0.36)** | **-0.62(-1.09,-0.14)** | -0.55 (-1.55,0.45) | -0.21 (-0.86,0.43) | -0.40 (-1.01,0.21) | -0.45 (-1.25,0.36) | **CTRL** |

Notes:Results from indirect comparisons (network meta-analysis) are shown in the lower left triangle, and direct comparisons (pairwise meta-analysis) are shown in the upper right triangle. Values represent standardized mean differences (SMDs) with 95% credible intervals (CIs); values > 0 favor the column-defining treatment. Statistically significant results are shown in bold blue font.

MBI :Mindfulness;CBT :Cognitive behavioral therapy;BEHAVE :Behavioral training; PHYS :Physical activity;SENS :Sensory therapy; TAFI :Technology- and family-based intervention;OTH :Other therapies;CTRL :Control group.

**Supplementary Table S15 –** Depression: league table.

| | | | | | | |
|---|---|---|---|---|---|---|
| **MBI** | -0.08 (-0.47,0.32) | -0.00 (-0.38,0.38) | 0.34 (-0.29,0.97) | 0.02 (-0.50,0.53) | -0.14 (-0.77,0.49) | 0.26 (-0.07,0.59) |
| 0.08 (-0.32,0.47) | **CBT** | 0.08 (-0.36,0.51) | 0.42 (-0.13,0.97) | 0.09 (-0.46,0.64) | -0.07 (-0.68,0.55) | **0.33 (0.03,0.64)** |
| 0.00 (-0.38,0.38) | -0.08 (-0.51,0.36) | **BEHAVE** | 0.34 (-0.30,0.99) | 0.02 (-0.55,0.58) | -0.14 (-0.78,0.50) | 0.26 (-0.08,0.60) |
| -0.34 (-0.97,0.29) | -0.42 (-0.97,0.13) | -0.34 (-0.99,0.30) | **PHYS** | -0.33 (-1.06,0.40) | -0.49 (-1.26,0.29) | -0.08 (-0.64,0.47) |
| -0.02 (-0.53,0.50) | -0.09 (-0.64,0.46) | -0.02 (-0.58,0.55) | 0.33 (-0.40,1.06) | **TAFI** | -0.16 (-0.88,0.56) | 0.24 (-0.24,0.72) |
| 0.14 (-0.49,0.77) | 0.07 (-0.55,0.68) | 0.14 (-0.50,0.78) | 0.49 (-0.29,1.26) | 0.16 (-0.56,0.88) | **OTH** | 0.40 (-0.14,0.94) |
| **-0.26 (-0.59,0.07)** | **-0.33 (-0.64,-0.03)** | **-0.26 (-0.60,0.08)** | 0.08 (-0.47,0.64) | **-0.24 (-0.72,0.24)** | **-0.40 (-0.94,0.14)** | **CTRL** |

Notes: Results from indirect comparisons (network meta-analysis) are shown in the lower left triangle, and direct comparisons (pairwise meta-analysis) are shown in the upper right triangle. Values represent standardized mean differences (SMDs) with 95% credible intervals (CIs); values > 0 favor the column-defined intervention. Statistically significant results (CI does not cross 0) are shown in bold blue. Comparisons with CI boundaries close to 0 are considered potentially suggestive but not conclusive.

MBI: Mindfulness-based intervention; CBT: Cognitive behavioral therapy; BEHAVE: Behavioral training; PHYS: Physical activity; TAFI: Technology- and family-based intervention; OTH: Other therapies; CTRL: Control group.

**Supplementary Table S16 –** Quality of Life: league table.

| | | | | | | |
|---|---|---|---|---|---|---|
| **PHYS** | -0.66 (-6.59,5.27) | -2.82(-11.41,5.7) | -3.34 (-9.52,2.84) | -4.15(-10.27,1.98) | -5.32(-9.71,-0.94) | -4.16 (-8.88,0.56) |
| 0.66 (-5.27,6.59) | **TAFI** | -2.16(-10.44,6.1) | -2.68 (-9.78,4.42) | -3.48 (-9.13,2.16) | -4.66(-8.70,-0.62) | -3.50 (-8.88,1.88) |
| 2.82(-5.78,11.41) | 2.16 (-6.12,10.44) | **SENS** | -0.52 (-9.87,8.83) | -1.33 (-9.78,7.13) | -2.50 (-9.71,4.70) | -1.34 (-9.37,6.69) |
| 3.34 (-2.84,9.52) | 2.68 (-4.42,9.78) | 0.52 (-8.83,9.87) | **CBT** | -0.81 (-8.08,6.46) | -1.98 (-7.84,3.87) | -0.82 (-7.39,5.74) |
| 4.15(-1.98,10.27) | 3.48 (-2.16,9.13) | 1.33 (-7.13,9.78) | 0.81 (-6.46,8.08) | **MBI** | -1.18 (-5.53,3.17) | -0.01 (-5.63,5.60) |
| **5.32 (0.94,9.71)** | **4.66 (0.62,8.70)** | 2.50 (-4.70,9.71) | 1.98 (-3.87,7.84) | 1.18 (-3.17,5.53) | **BEHAVE** | 1.16 (-2.39,4.72) |
| 4.16 (-0.56,8.88) | 3.50 (-1.88,8.88) | 1.34 (-6.69,9.37) | 0.82 (-5.74,7.39) | 0.01 (-5.60,5.63) | -1.16 (-4.72,2.39) | **CTRL** |

Notes: Results from indirect comparisons (network meta-analysis) are shown in the lower left triangle, and direct comparisons (pairwise meta-analysis) are shown in the upper right triangle. Values represent standardized mean differences (SMDs) with 95% credible intervals (CIs); values > 0 favor the column-defined intervention. Statistically significant results (CI does not cross 0) are shown in bold blue. Comparisons with CI boundaries close to 0 are considered potentially suggestive but not conclusive.

MBI :Mindfulness;CBT :Cognitive behavioral therapy;BEHAVE :Behavioral training; PHYS :Physical activity;SENS :Sensory therapy; TAFI :Technology- and family-based intervention;CTRL :Control group.

**Supplementary Figure S10** – Anxiety: Forest plot of pairwise com parisons.

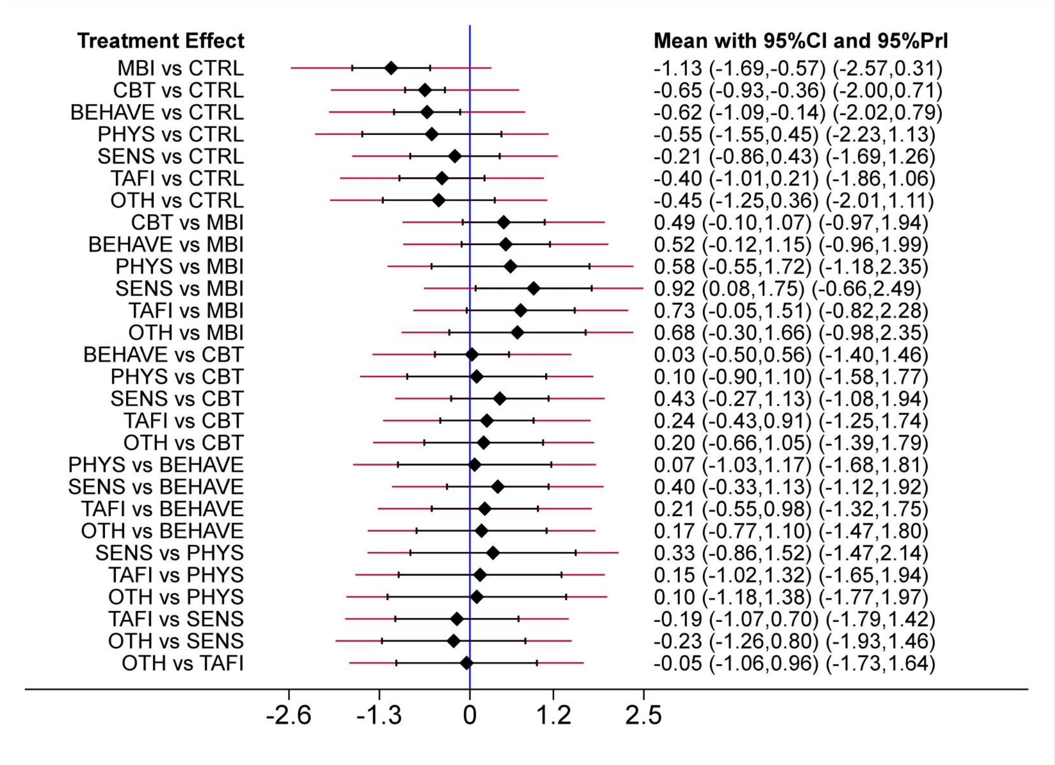


**Supplementary Figure S11** – Anxiety: cumulative probability plot

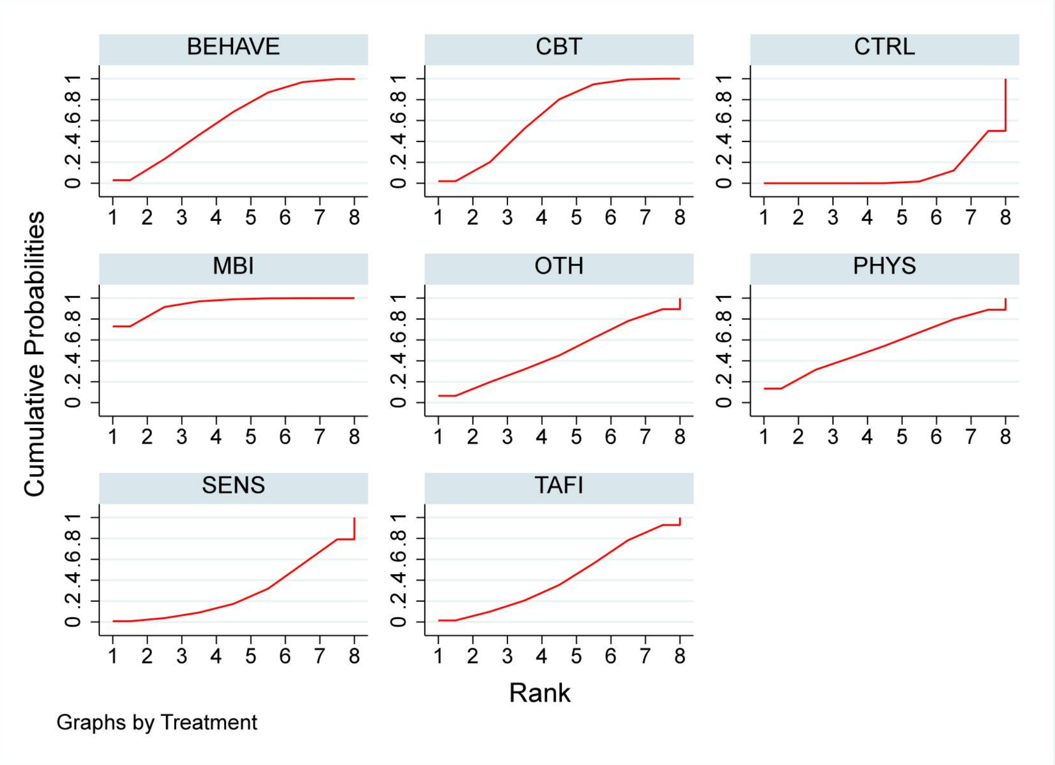

**Supplementary Figure S12** – Anxiety: Subgroup analysis by gender.

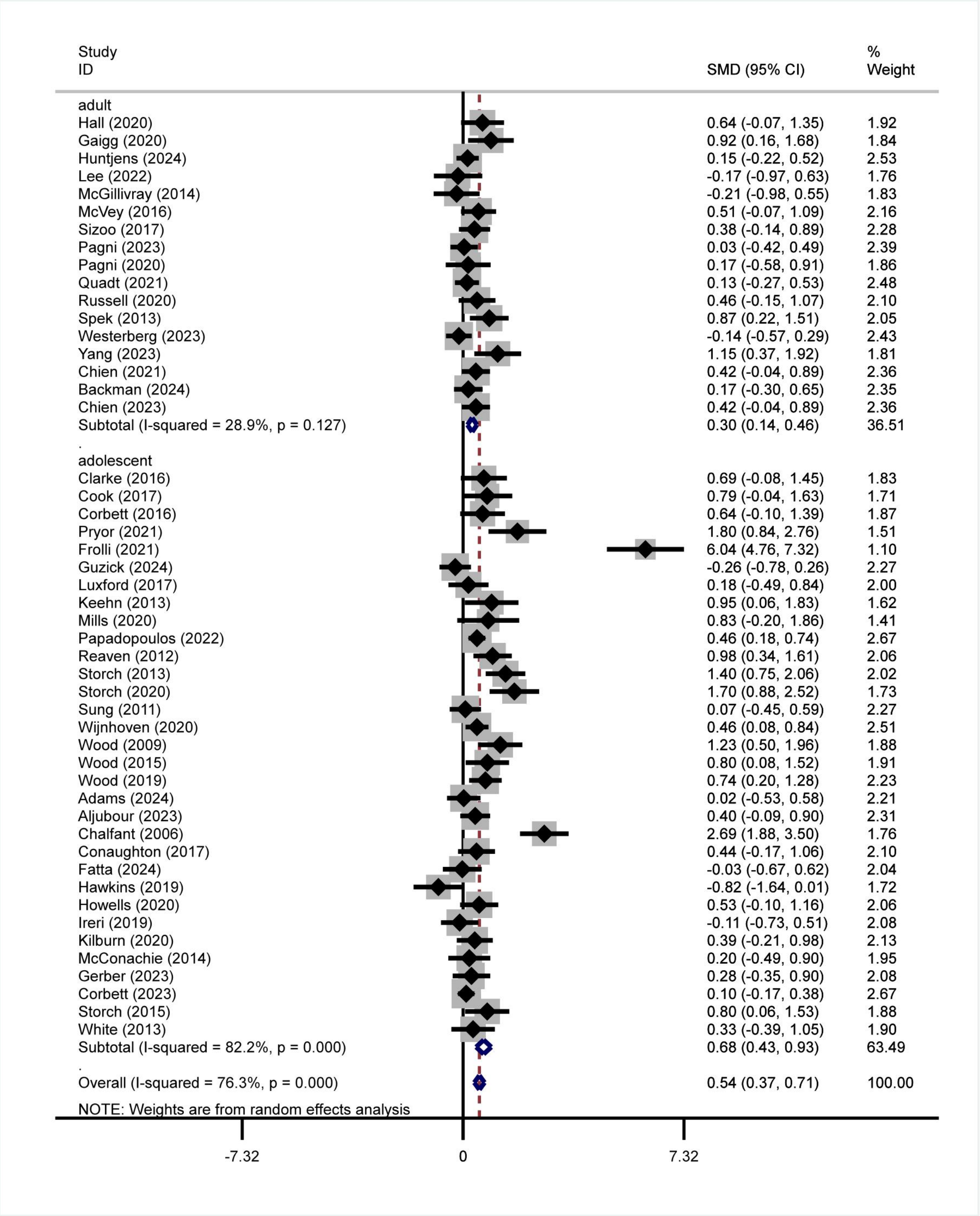

**Supplementary Figure S13** – Anxiety: Subgroup analysis by intervention type.

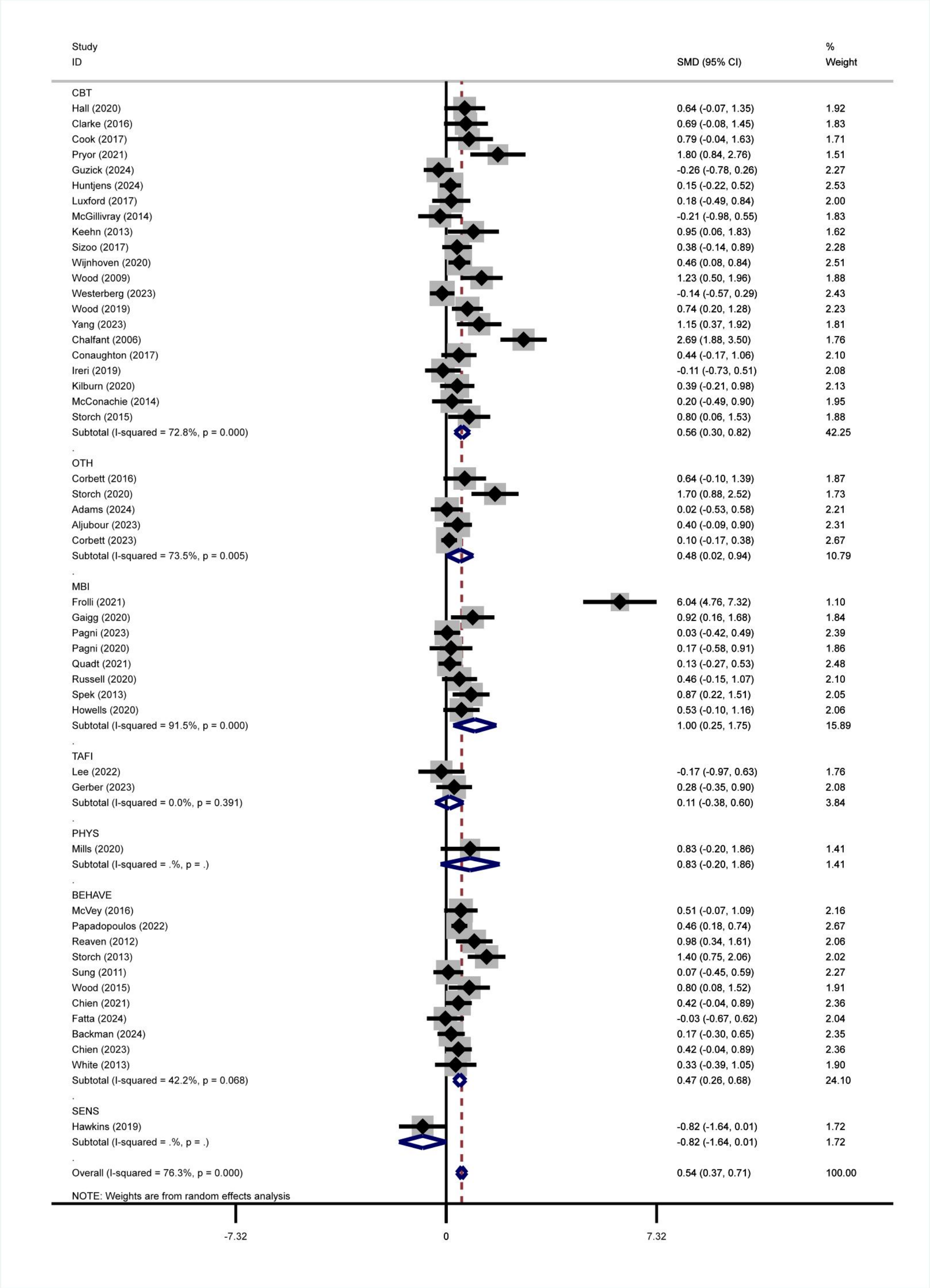

**Supplementary Figure S14** – Anxiety: Subgroup analysis by intervention duration.

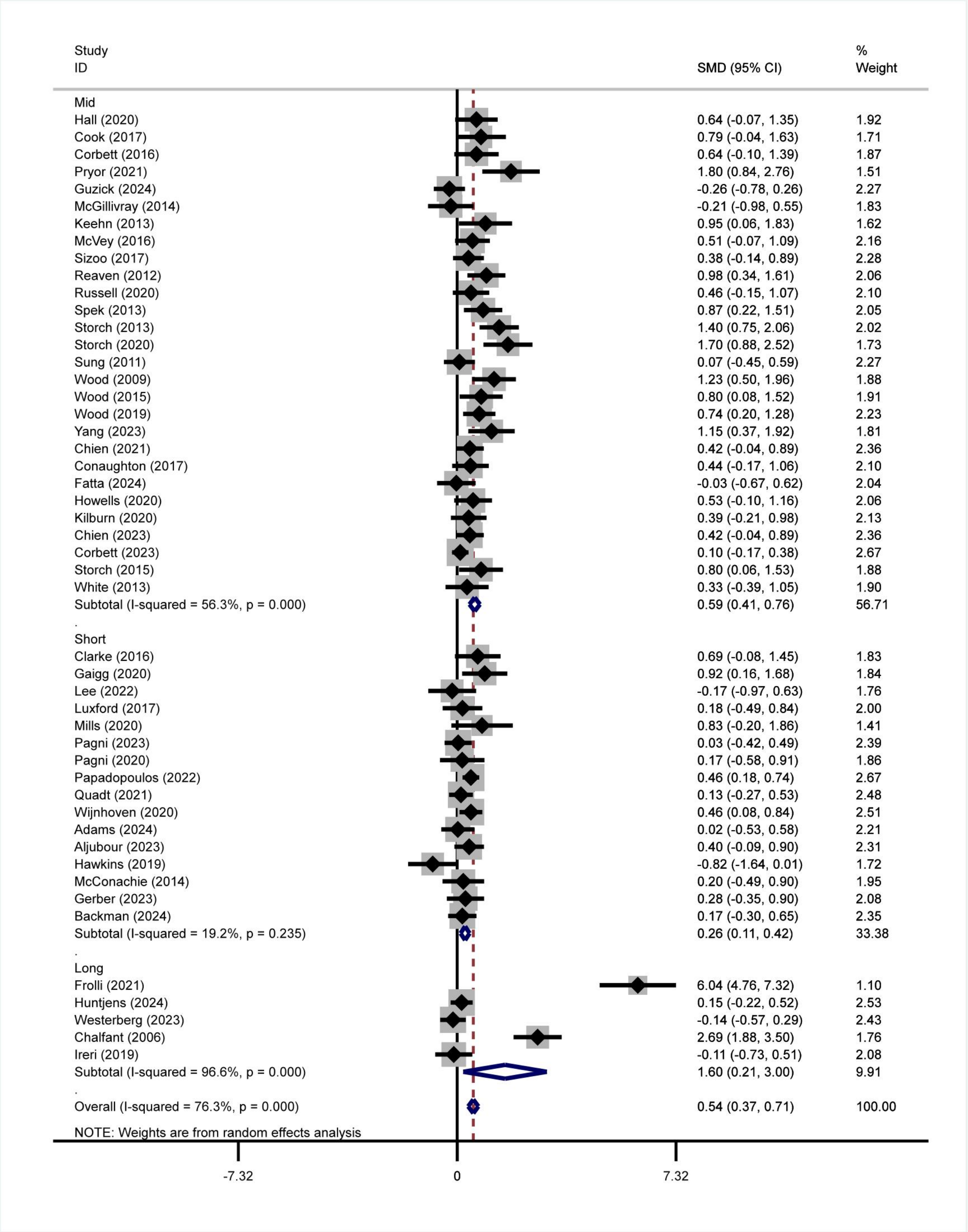

**Supplementary Figure S15 –** Depression: network evidence plot.

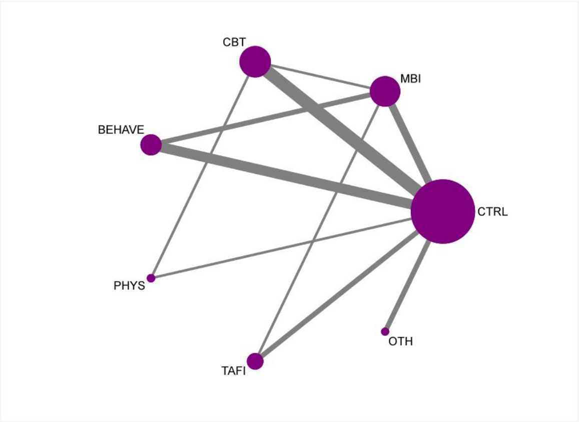


**Supplementary Figure S16** – Depression:Contribution plot of the network.

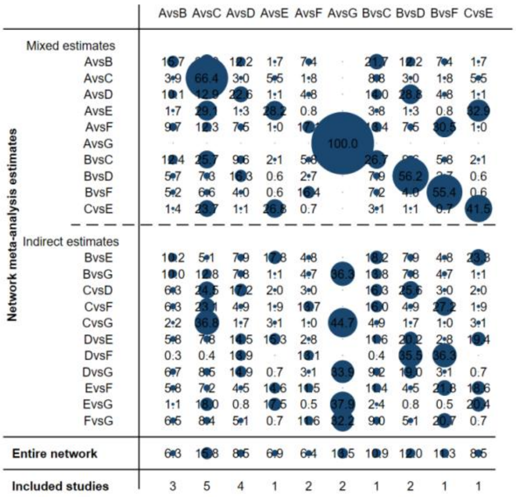


**Supplementary Figure S17 –** Depression: comparison-adjusted funnel plot.

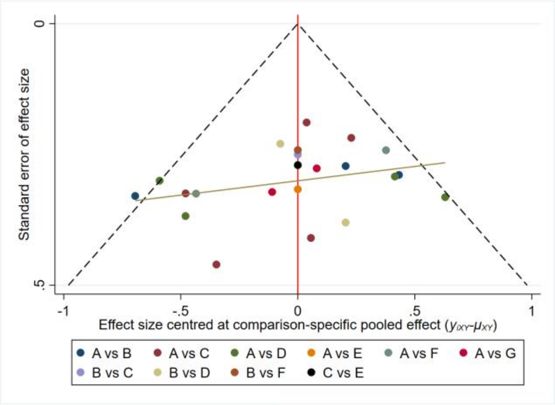

**Supplementary Figure S18 –** Depression: network forest.

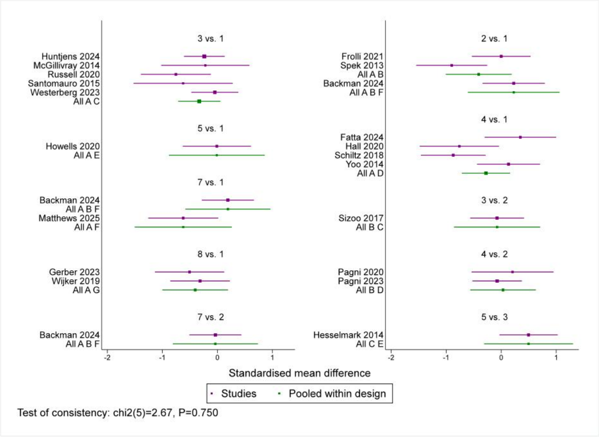


**Supplementary Figure S19** – Depression: Forest plot of pairwise c

omparisons.

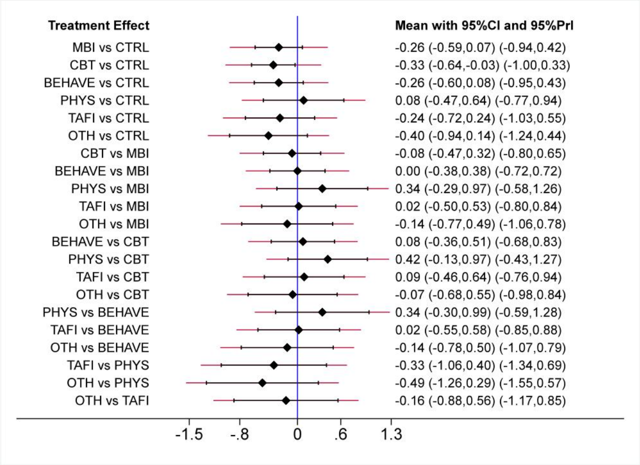


**Supplementary Figure S20 –** Depression: cumulative probability plot .

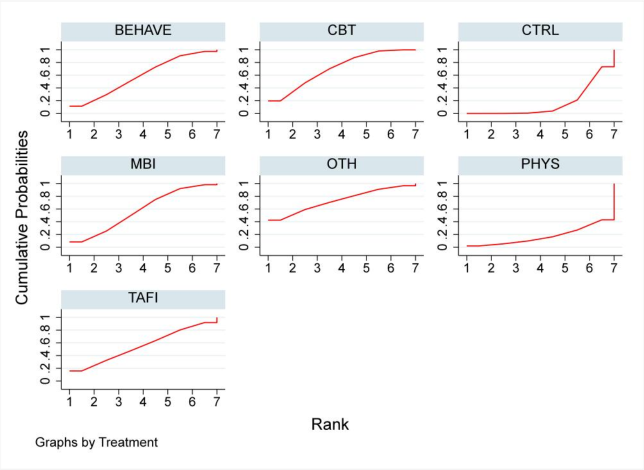

**Supplementary Figure S21** – Depression: Subgroup analysis by gender.

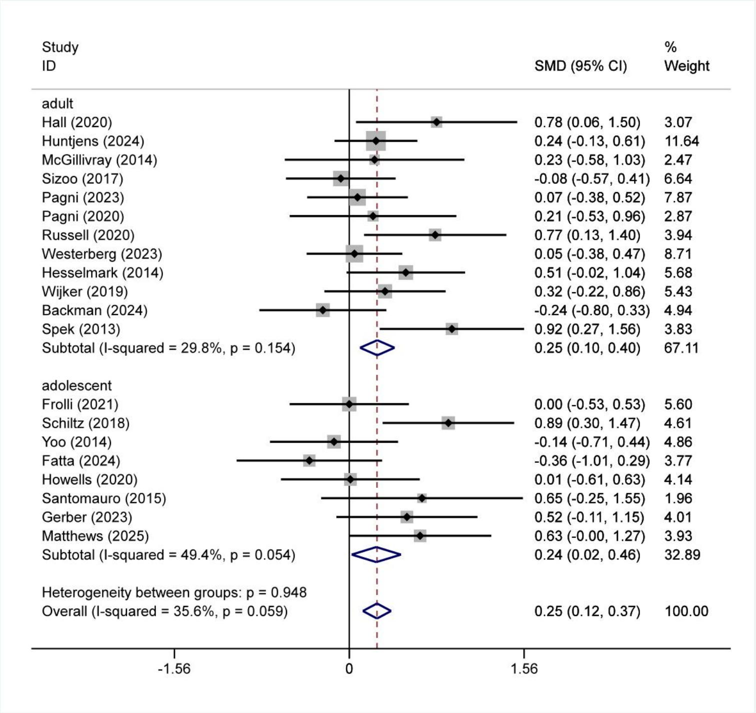


**Supplementary Figure S22** – Depression: Subgroup analysis by int ervention type.

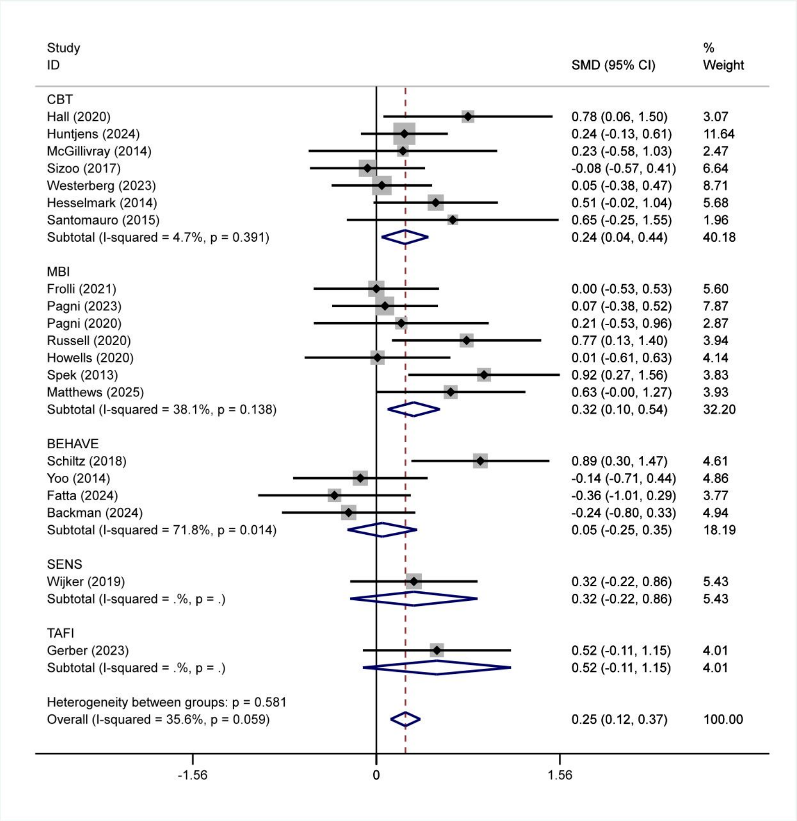

**Supplementary Figure S23** – Depression: Subgroup analysis by intervention duration.

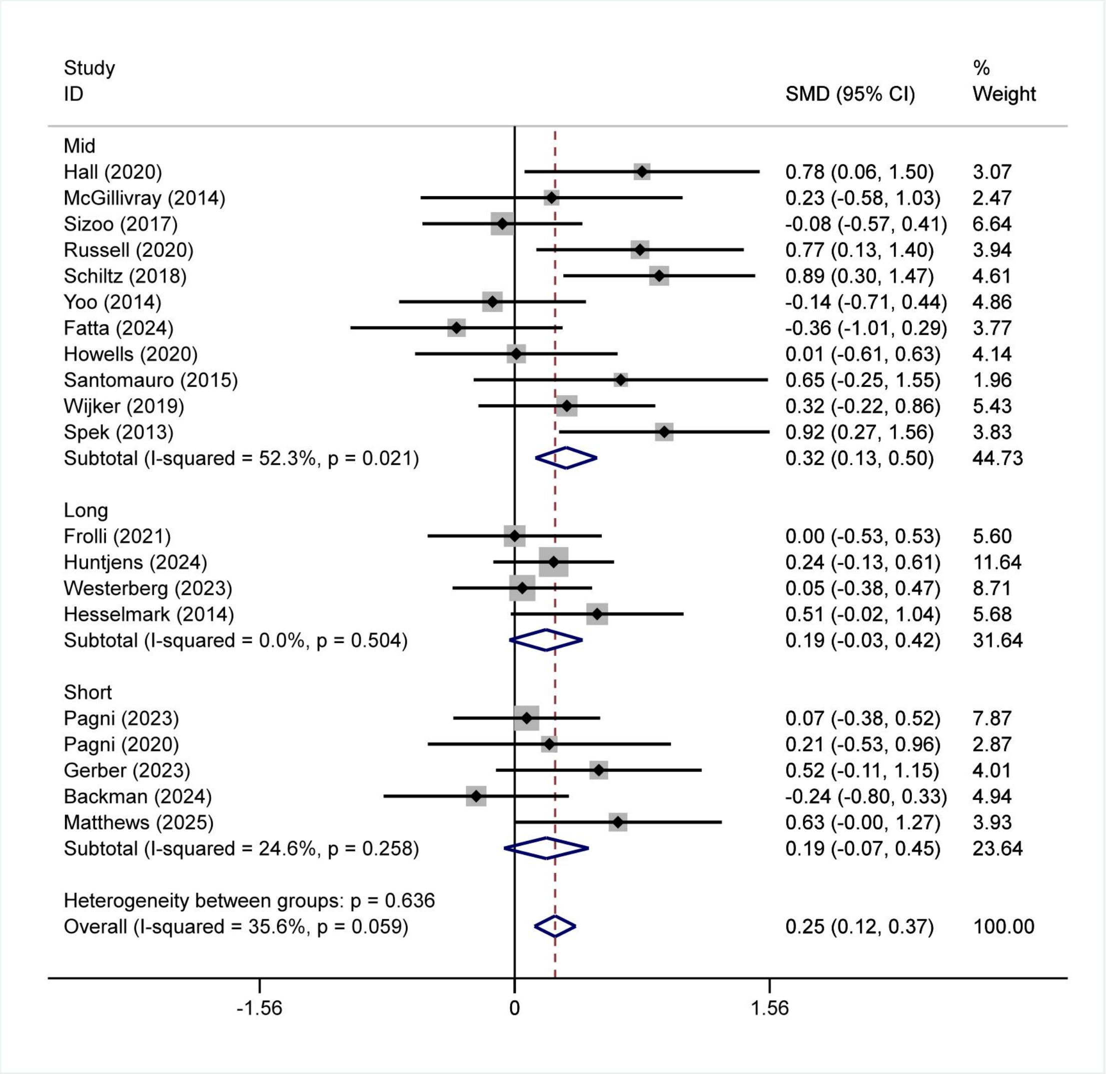

**Supplementary Figure S24 –** Quality of Life: network evidence plot.

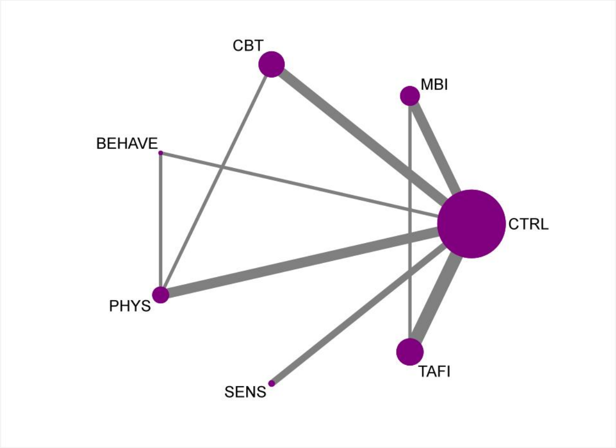


**Supplementary Figure S25** – Quality of Life:Contribution plot of the network.

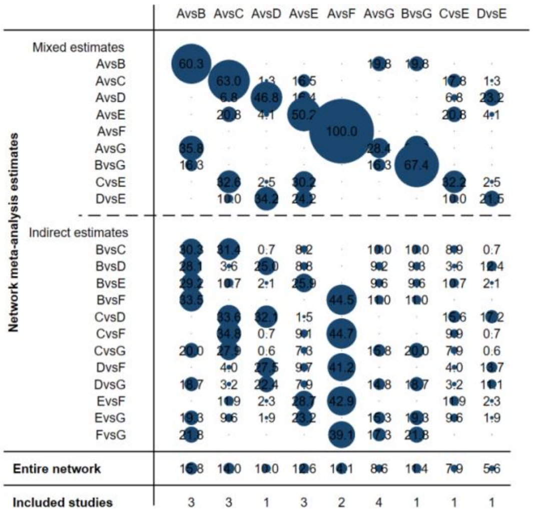


**Supplementary Figure S26 – Quality of Life: comparison-adjusted funnel plot.**

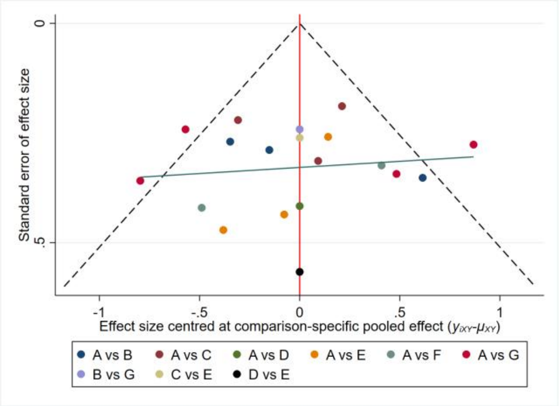

**Supplementary Figure S27 –** Quality of Life: network forest.

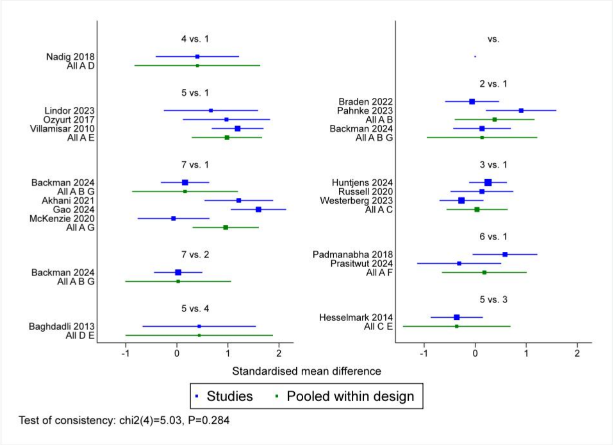


**Supplementary Figure S28** – Quality of Life: Forest plot of pairwi se comparisons.

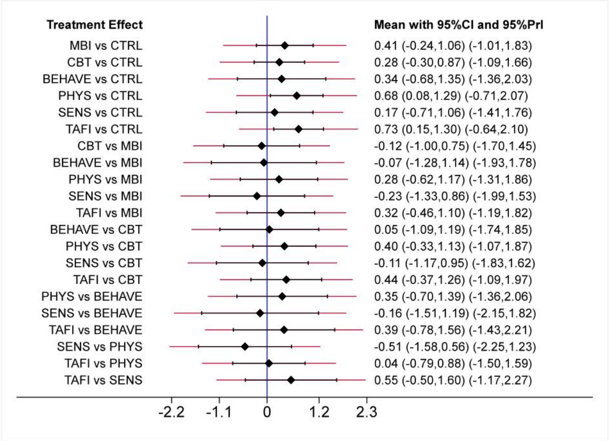


**Supplementary Figure S29 –** Quality of Life: cumulative probability plot .

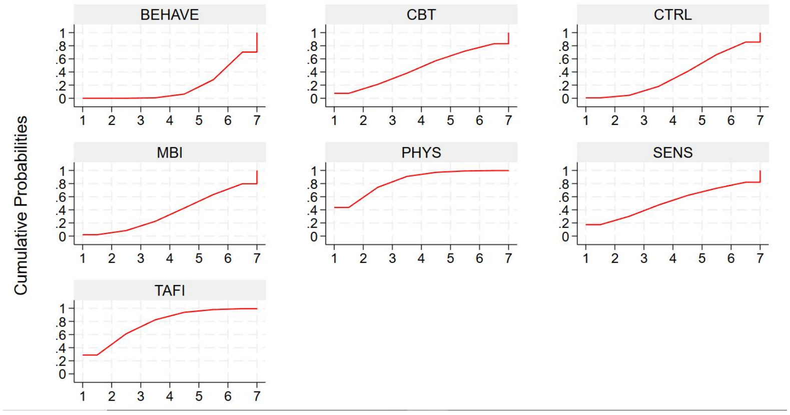

**Supplementary Figure S30** – Quality of Life: Subgroup analysis by gender.

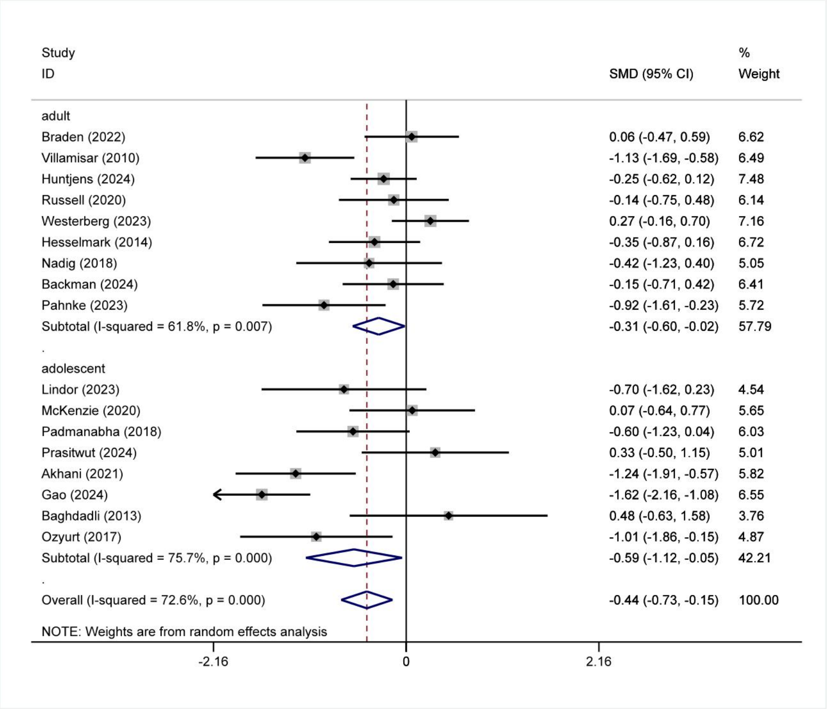


**Supplementary Figure S31** – Quality of Life: Subgroup analysis b y intervention type.

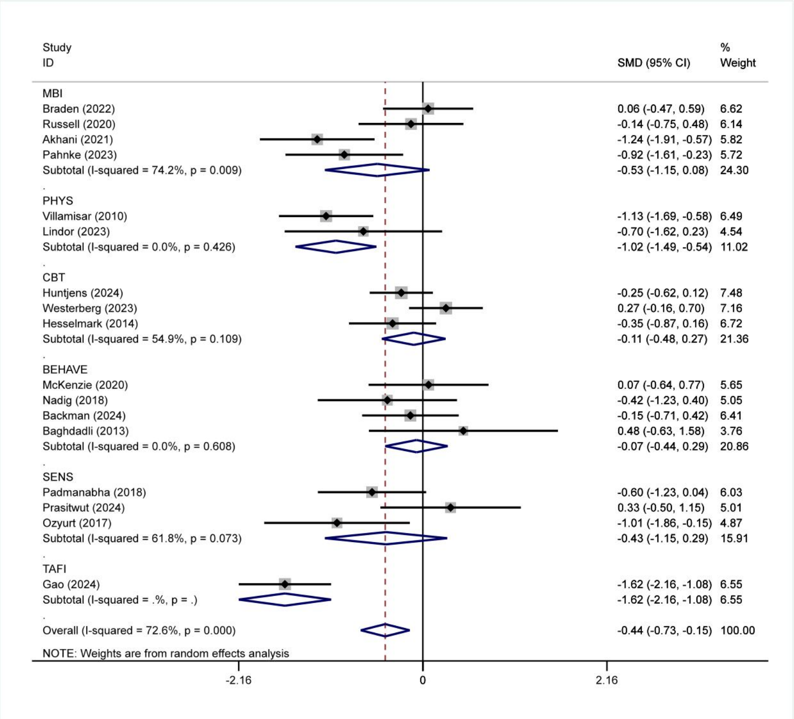

**Supplementary Figure S32** – Quality of Life: Subgroup analysis by intervention duration.

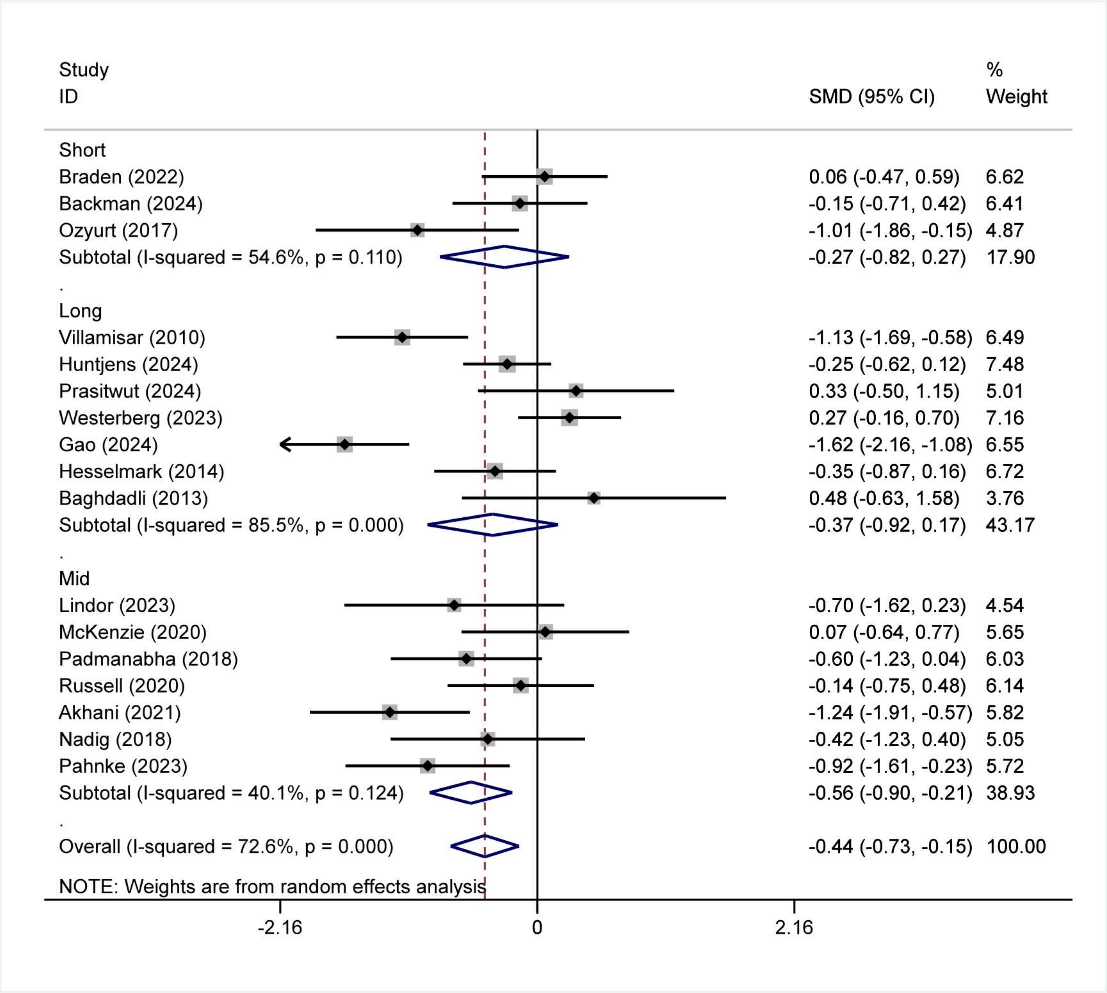

**Supplementary Figure S33 –** Anxiety: Risk of bias network for pairwise comparisons.

Notes:The colours in the circles indicate the distribution of RoB 2.0 ratings within each intervention: green = predominantly low risk of bias, yellow = moderate risk, and red = high risk.The colours of the connecting lines indicate the overall risk of bias judgment for each pairwise comparison: green = low RoB comparison, yellow = moderate RoB comparison, and red = high RoB comparison.This figure summarizes the comparative methodological quality across interventions in the anxiety outcome network.

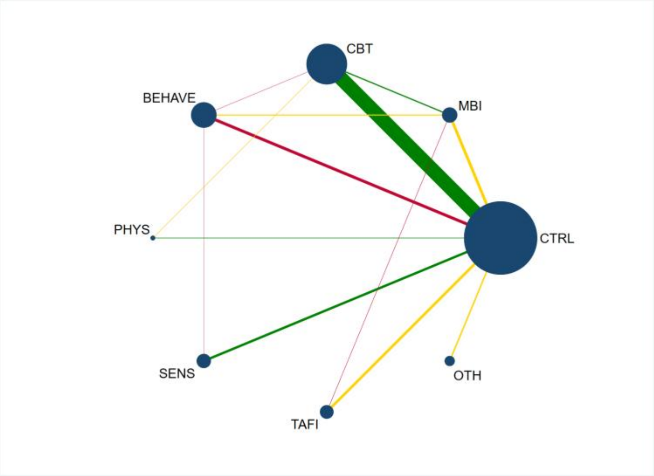


**Supplementary Figure S34 –** Anxiety: Contribution of low, moderate, and high RoB comparisons to each network estimate.

Notes:Based on the risk of bias (RoB) assessment for each comparison and the contribution matrix detailing their impact on the network estimates, the following bar chart displays the proportion of evidence contributed by comparisons at low risk (green), moderate risk (yellow), and high risk (red) of bias.Each bar represents a network meta-analysis (NMA) relative treatment effect, with study limitations in each direct comparison indicated at the beginning of the graph.

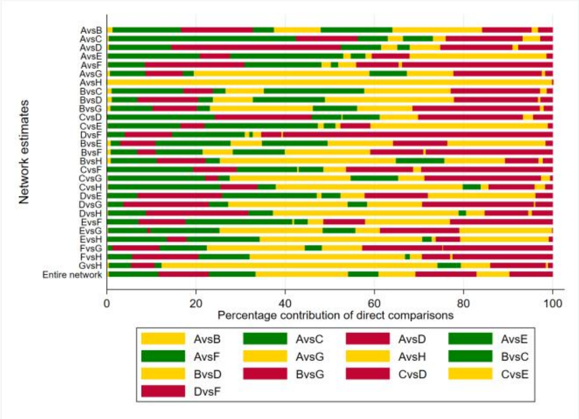

**Supplementary Figure S35** – Depression: Risk of bias network for pairwise comparisons.

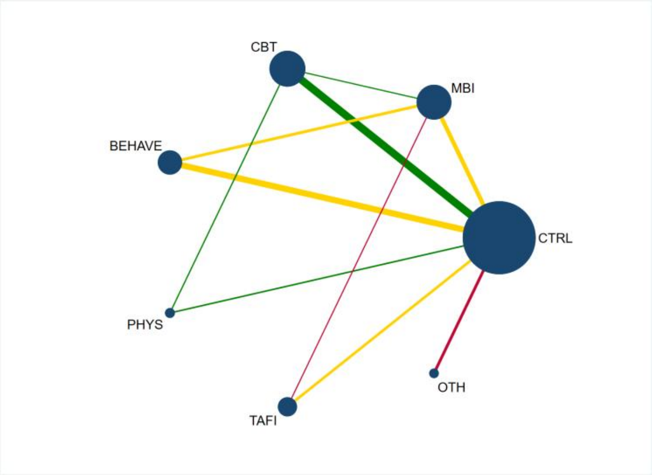


**Supplementary Figure S36** –Depression: Contribution of low, moderate, and high RoB comparisons to each network estimate.

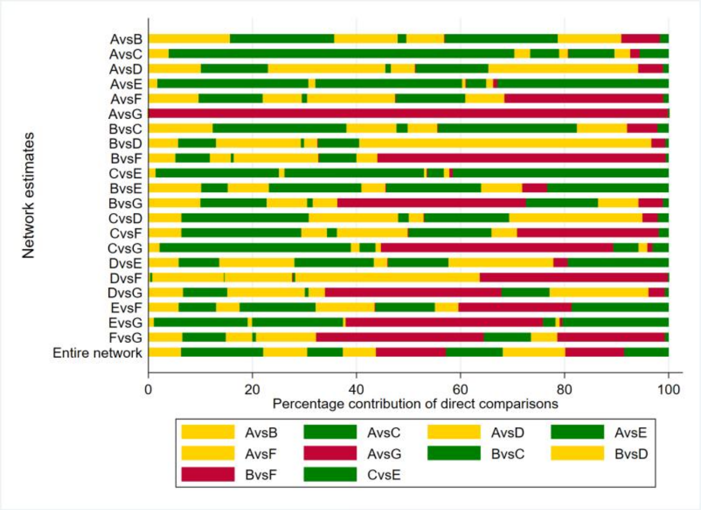

**Supplementary Figure S37** – Quality of Life: Risk of bias network for pairwise comparisons.

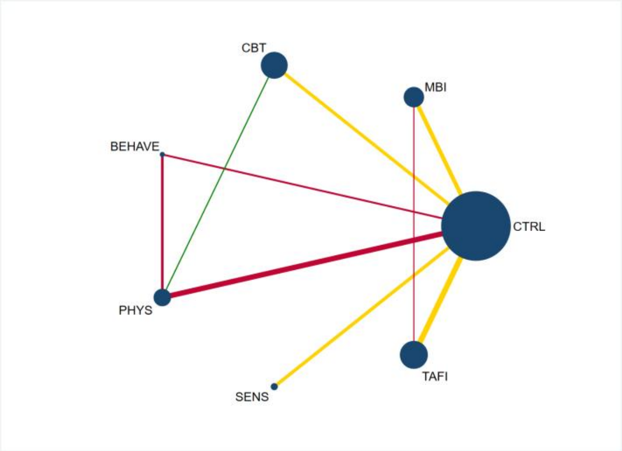


**Supplementary Figure S38** – Quality of Life: Contribution of low, moderate, and high RoB comparisons to each network estimate.

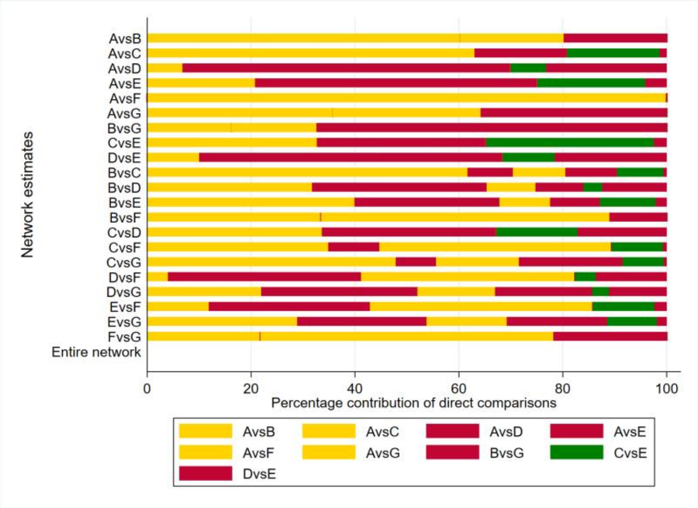


Notes:This figure displays the percentage contribution of each direct comparison to the estimated relative treatment effects in the network meta-analysis for quality of life (QoL) outcomes. Each horizontal bar represents a network estimate, indicating the proportion of evidence derived from comparisons assessed as low risk of bias (green), moderate risk of bias (yellow), and high risk of bias (red) according to the RoB 2.0 tool.The judgments about risk of bias for each direct comparison are shown at the beginning of the graph.Due to the limited number of large-scale, rigorously designed trials reporting QoL outcomes among ASD populations, a higher proportion of moderate and high risk of bias comparisons is observed in this network. This highlights the need for cautious interpretation and further high-quality research on non-pharmacological interventions for QoL improvement.

## **Supplementary Table S17** – Table of reasons for downgrading.

Notes:This table summarizes the reasons for downgrading the certainty of evidence for each pairwise comparison in the network meta-analysis, based on the GRADE framework for network meta-analysis proposed by Higgins et al. (2014)[1] .
Downgrading domains include: study limitations, imprecision, inconsistency, indirectness, and publication bias. The final GRADE rating (very low, low, moderate, high) reflects the cumulative impact of downgrading across these domains for each comparison.The majority of comparisons were downgraded due to risk of bias (study limitations) and potential publication bias, particularly for comparisons involving limited or small-scale studies.

| | | Study limitations | Imprecision | Inconsistency | Indirectness | Publication bias | GRADE |
|---|---|---|---|---|---|---|---|
| Anxiety | AvsB | Downgrade | No downgrade | No downgrade | No downgrade | Downgrade | LOW |
| | AvsC | Downgrade | No downgrade | No downgrade | No downgrade | No downgrade | MODERATE |
| | AvsD | Downgrade | No downgrade | No downgrade | No downgrade | No downgrade | MODERATE |
| | AvsE | Downgrade | No downgrade | No downgrade | No downgrade | Downgrade | LOW |
| | AvsF | Downgrade | No downgrade | No downgrade | No downgrade | Downgrade | LOW |
| | AvsG | Downgrade | No downgrade | No downgrade | No downgrade | No downgrade | MODERATE |
| | AvsH | Downgrade | No downgrade | Downgrade | No downgrade | Downgrade | VERY LOW |
| | BvsC | Downgrade | No downgrade | No downgrade | No downgrade | Downgrade | LOW |
| | BvsD | Downgrade | No downgrade | No downgrade | No downgrade | No downgrade | MODERATE |
| | BvsG | Downgrade | No downgrade | No downgrade | No downgrade | Downgrade | LOW |
| | CvsD | Downgrade | No downgrade | No downgrade | No downgrade | Downgrade | LOW |
| | CvsE | Downgrade | No downgrade | No downgrade | No downgrade | Downgrade | LOW |
| | DvsF | Downgrade | No downgrade | No downgrade | No downgrade | Downgrade | LOW |
| | A = CTRL (Control group),B = MBI (Mindfulness-Based Intervention),C = CBT (Cognitive Behavioral Therapy), D = BEHAVE (Behavioral and Functional Training),E = PHYS (Physical Activity),F = SENS (Sensory Therapy), G = TAFI (Technology- and Family-Based Intervention),H = OTH (Other Therapies). | | | | | | |
| | | Study limitations | Imprecision | Inconsistency | Indirectness | Publication bias | GRADE |
| Depression | AvsB | Downgrade | No downgrade | No downgrade | No downgrade | Downgrade | LOW |
| | AvsC | No downgrade | No downgrade | No downgrade | No downgrade | Downgrade | MODERATE |
| | AvsD | Downgrade | No downgrade | No downgrade | No downgrade | No downgrade | MODERATE |
| | AvsE | No downgrade | No downgrade | No downgrade | No downgrade | Downgrade | MODERATE |
| | AvsF | Downgrade | No downgrade | No downgrade | No downgrade | Downgrade | LOW |
| | AvsG | Downgrade | No downgrade | Downgrade | No downgrade | Downgrade | VERY LOW |
| | BvsC | No downgrade | No downgrade | No downgrade | No downgrade | Downgrade | MODERATE |
| | BvsD | Downgrade | No downgrade | No downgrade | No downgrade | Downgrade | LOW |
| | BvsF | Downgrade | No downgrade | No downgrade | No downgrade | Downgrade | LOW |
| | CvsE | Downgrade | No downgrade | No downgrade | No downgrade | Downgrade | LOW |
| | A = CTRL (Control group),B = MBI (Mindfulness-Based Intervention),C = CBT (Cognitive Behavioral Therapy), D = BEHAVE (Behavioral and Functional Training),E = PHYS (Physical Activity), F = TAFI (Technology- and Family-Based Intervention),G = OTH (Other Therapies). | | | | | | |

| | | Study limitations | Imprecision | Inconsistency | Indirectness | Publication bias | GRADE |
|---|---|---|---|---|---|---|---|
| **Quality of Life** | AvsB | Downgrade | No downgrade | No downgrade | No downgrade | Downgrade | LOW |
| | AvsC | Downgrade | No downgrade | No downgrade | No downgrade | Downgrade | LOW |
| | AvsD | Downgrade | No downgrade | No downgrade | No downgrade | Downgrade | LOW |
| | AvsE | Downgrade | No downgrade | No downgrade | No downgrade | Downgrade | LOW |
| | AvsF | Downgrade | No downgrade | Downgrade | No downgrade | Downgrade | VERY LOW |
| | AvsG | Downgrade | No downgrade | No downgrade | No downgrade | Downgrade | LOW |
| | BvsG | Downgrade | No downgrade | No downgrade | No downgrade | Downgrade | LOW |
| | CvsE | Downgrade | No downgrade | No downgrade | No downgrade | Downgrade | LOW |
| | DvsE | Downgrade | No downgrade | No downgrade | No downgrade | Downgrade | LOW |
| | A = CTRL (Control group),B = MBI (Mindfulness-Based Intervention),C = CBT (Cognitive Behavioral Therapy), D = BEHAVE (Behavioral and Functional Training),E = PHYS (Physical Activity),F = SENS (Sensory Therapy), G = TAFI (Technology- and Family-Based Intervention). | | | | | | |
